# Synthesis and AChE-Inhibitory Activity of New Benzimidazole Derivatives

**DOI:** 10.3390/molecules24050861

**Published:** 2019-02-28

**Authors:** Ulviye Acar Cevik, Begüm Nurpelin Saglik, Serkan Levent, Derya Osmaniye, Betul Kaya Cavuşoglu, Yusuf Ozkay, Zafer Asim Kaplancikli

**Affiliations:** 1 Department of Pharmaceutical Chemistry, Faculty of Pharmacy, Anadolu University, 26210 Eskişehir, Turkey; uacar@anadolu.edu.tr (U.A.C.); bnsaglik@anadolu.edu.tr (B.N.S.); serkanlevent@anadolu.edu.tr (S.L.); dosmaniye@anadolu.edu.tr (D.O.); betulkaya@anadolu.edu.tr (B.K.C.); zakaplan@anadolu.edu.tr (Z.A.K.); 2 Doping and Narcotic Compounds Analysis Laboratory, Faculty of Pharmacy, Anadolu University, 26210 Eskişehir, Turkey

**Keywords:** benzimidazole, triazole, acetylcholinesterase, butyrylcholinesterase, docking study

## Abstract

Alzheimer’s disease (AD), one of the main causes of aged dementia, is a progressive and degenerative neurological disorder characterized by loss of cognition and memory. Although the symptomatic treatment of AD, particularly acetylcholinesterase inhibitors (AChEIs) based on the ‘cholinergic hypothesis’, has been successful in clinic, at present there is no cure for this disease. In this study, we designed compounds carrying benzimidazole and triazole rings on the same chemical skeleton so as to investigate their potential acetylcholinesterase and butyrylcholinesterase activity. Furthermore, molecular modeling study was performed to determine the binding mode of the best inhibitor to the AChE. Among them, compounds **3d** and **3h**, which featured 3,4-dihydroxy substitution at the phenyl ring and 5(6)-chloro substitution at the benzimidazole ring were found to be potent inhibitors of AChE. The inhibition kinetics of the two most active derivatives **3d** and **3h** were further studied. The kinetic displayed increasing slope and increasing intercept, which is consistent with a mixed inhibition. The IC_50_ and K_i_ values of **3d** are 31.9 ± 0.1 nM and 26.2 nM, respectively. Compound **3h** exhibited IC_50_ of 29.5 ± 1.2 nM and K_i_ of 24.8 nM. The above data compared favorably with data for donepezil (21.8 ± 0.9 nM) the reference compound in our study.

## 1. Introduction

Neurodegeneration is defined as a progressive and often untreatable sequence that causes loss of specific neuron types, disregulation of neurons, distribution and central nervous system (CNS) dysfunction. Alzheimer’s disease (AD), which is one of the most important central nervous system disorders among various neurodegenerative diseases, is the fourth leading cause of death among the elderly. According to the World Alzheimer’s Report 2016, 46.8 million people worldwide were predicted by dementia in 2015 and the number of patients was predicted to triple by 2050. AD clinically includes the progressive degeneration of brain tissue that is influenced by the absence in acetylcholine (ACh) and has multifactorial pathology [1,2,3,4,5]. Accumulation of extracellular amyloid-beta (Aβ) in senile plaques, loss of cholinergic activity in certain parts of brain and intracellular neurofibrillary tangles including the hyperphosphorylated tau protein as well as neuroinflammation, responsible for neurodegenerative processes observed in AD [6,7,8].

The cholinergic hypothesis is one of the oldest, most robust and clinically confirmed among cases of mild severe dementia and Alzheimer’s hypotheses that suggest a relationship between the onset and progression of the disease. One of the major ways to increase the level of acetylcholine (ACh), which plays an important role in attention, learning, memory and motivation, is the inhibition of cholinesterase enzymes (ChEs). Cholinesterases belonging to the class of hydrolase enzyme are a widely distributed enzyme classified as acetylcholinesterase (AChE) and butyrylcholinesterase (BChE). In patients with AD, an imbalance occurs between AChE and BChE. AChE inhibitors are preferred in the treatment of AD to protect the AChE norms because AChE exhibits more hydrolytic activity than BChE. Crystal structural studies with both AChE enzyme have revealed that they have different binding sites, containing the aromatic patch (AP), oxyanionic hole, peripheral anionic site (PAS), catalyist active site (CAS), acyl site and anionic site (AS). To improve effective acetylcholinesterase inhibitors (AChEI), acetylcholinesterase activity must be inhibited in the oxidation hole region (AS) and in the peripheral anionic region (PAS), because it causes accumulation of bound AChE amyloid peptide plaques in PAS [9,10,11,12,13,14,15,16,17]. Although there are many ongoing studies for the treatment of AD, only some drugs have been accepted by the FDA, such as donepezil, rivastigmin and tacrine [18].

Bioisosterism is a special molecular modification process that is applied over a lead compound (LC) and is a medicinal chemistry strategy for the rational design of new drugs. The capability of a group of bioisosteres to prompt similar biological activity has been recognized in compounds with common physicochemical properties such as benzazoles. They provide a single point for chemical variation, with a heteroatom (N, O, S) at the 1-position and with a nitrogen at the 3-position. This property facilitated a systematic investigation [19,20,21]. When the literature studies are evaluated, it can be learned that benzazole derivatives have inhibitory activity on acetylcholinesterase enzyme [17,22,23,24,25,26,27,28,29,30]. In addition, some benzimidazole derivatives have been shown to be useful as amyloid imaging probes because of their high binding affinity to Aβ aggregates and high uptake into the brain [31]. Also, benzimidazole scaffold is the ring isoster of indanone pharmacophore of donepezil which is one of the most important AChEI [32]. Benzimidazoles with different biological activities were thought to be an appropriate starting point for AD.

Considering that there is a limited choice of medicines available for the treatment of AD, we focused on using benzimidazole as a lead compound to develop new drug candidates to combat AD. In this study, 16 new benzimidazole derivatives were synthesized and cholinesterase inhibition activity was evaluated.

## 2. Results and Discussion

### 2.1. Chemistry

**Scheme 1:**
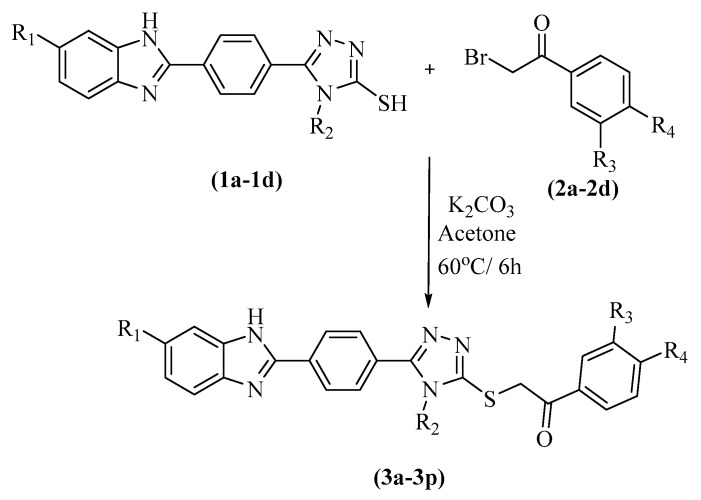
Synthesis way of the compounds **1a**–**d**, **3a**–**p**.

The syntheses of the target compounds **3a**–**p** were realized as summarized in Scheme 1. Known triazoles **1a**–**d** were synthesized according to our already published method [33,34]. The *S*-alkylation of the triazole ring of compounds **1a**–**d** with an appropriate 2-bromoacetophenone moiety **2a**–**d** using potassium carbonate as base generated the target derivatives **3a**–**p**.

### 2.2. Anticholinesterase Activity Assay

New benzimidazole-triazole derivatives were designed, synthesized, and evaluated for their potential to inhibit AChE and BChE. First, all the synthesized compounds were tested at 10^−3^ M and 10^−4^ M concentrations. Table 1 presents the acetylcholinesterase and butyrylcholinesterase inhibition of synthesized compounds and donepezil and tacrine at initial concentrations. According to activity results, the series generally displayed better inhibitor activity against AChE than BChE activity. At the 10^−4^ M concentration, compounds **3d**, **3h**, **3l** and **3p** indicated more than 50% activity. Then, the IC_50_ values of the active compounds were determined using 10^−3^–10^−9^ M concentrations against AChE along with the reference drug donepezil (Table 2). The IC_50_ value was calculated as 21.8 ± 0.9 nM for donepezil. The IC_50_ values of the compounds (5(6)chlorobenzimidazole) (**3d** and **3h**) and (5(6)fluorobenzimidazole) (**3l** and **3p**) were calculated as 31.9 ± 0.1 nM, 29.5 ± 1.2 nM and 169.4 ± 7.2 nM, 139.9 ± 5.1 nM, respectively for AChE. The compounds **3d**, **3h**, **3l** and **3p** among the series were those having the 3,4-dihydroxy substituent on the phenyl attached to benzimidazole. The strongest inhibitions against AChE were observed with **3d** and **3h** having a chloro substituent on the benzimidazole ring. The results show that replacing the methyl group attached to the triazole with an ethyl group does not create a significant activity change. The results demonstrated that the compounds **3d** and **3h** showed IC_50_ values similar activities compared to the reference drug against AChE.

### 2.3. Enzyme Kinetic Studies

**Figure 1:**
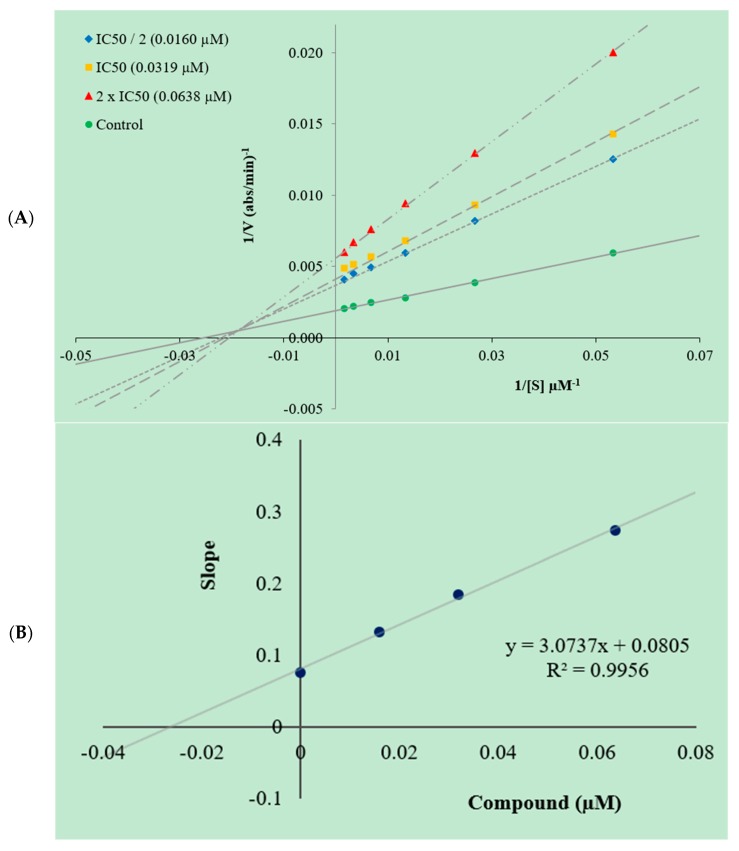
(**A**) Lineweaver-Burk plots for the inhibition of AChE by compound **3d**. [S], substrate concentration (μM); V, reaction velocity (abs/min)^−1^. (**B**) Secondary plot for calculation of steady-state inhibition constant (K_i_) of compound **3d**. Ki was calculated as 0.0262 μM.

**Figure 2:**
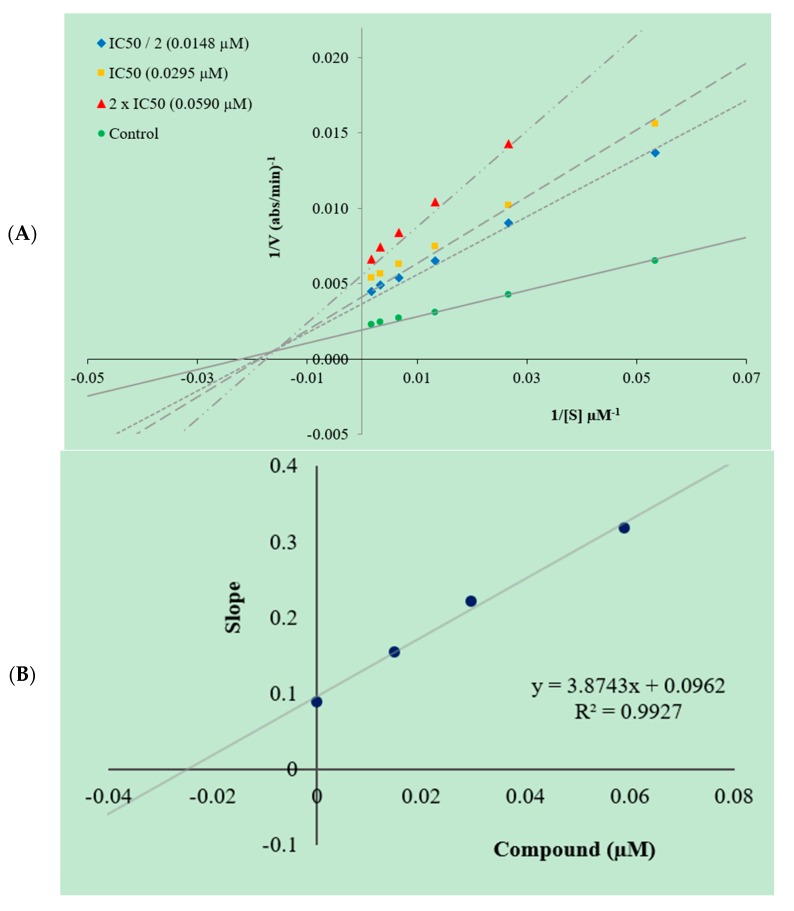
(**A**) Lineweaver-Burk plots for the inhibition of AChE by compound **3h**. [S], substrate concentration (μM); V, reaction velocity (abs/min)^−1^. (**B**) Secondary plot for calculation of steady-state inhibition constant (K_i_) of compound **3h**. K_i_ was calculated as 0.0248 μM.

Enzyme kinetic studies were carried out in order to determine the mechanism of AChE inhibition. The type of inhibition was forecast using linear Lineweaver-Burk plots. The substrate rate curves in the absence and presence of the most potent compounds **3d** and **3h** were recorded in the enzyme kinetics analysis. These compounds were prepared at concentrations of 2 × IC_50_, IC_50_ and IC_50_/2. The initial velocity measurements were recorded by using different substrate concentrations ranging from 600 µM to 18.75 µM for AChE enzyme. The secondary plots of the K_m_/V_max_ (slope) versus varying concentrations were used to calculate K_i_ (intercept on the *x*-axis) values of compounds [35]. The graphical analyses of the steady-state inhibition data for AChE enzyme are shown in Figure 1 and Figure 2, respectively.

Reversible inhibition consists of the following subtypes: mixed-type, competitive, uncompetitive, or noncompetitive. These types of inhibition can be determined with the help of Lineweaver-Burk plots. As known from Lineweaver-Burk graphics, mixed-type inhibition can be refereed if the lines do not cross *x*- or *y*-axis at the same point. The Lineweaver-Burk plots of compounds **3d** and **3h** are presented in Figure 1 and Figure 2. As can be understand from these figures, compounds **3d** and **3h** display mixed type inhibition for AChE enzyme. Namely, both compounds can bind to the free enzyme or the enzyme-substrate complex. Also, this means that tested compounds **3d** and **3h** are reversible inhibitors, so these compounds could bind to the enzyme by noncovalent interactions such as hydrophobic interactions, ionic bonds, and hydrogen bonds without forming any chemical bonds or reacting with the enzyme. It is known that these interactions occur quickly and can be easily reversed. Therefore, the enzyme and inhibitor complex is rapidly degraded immediately prior to irreversible inhibition. This feature of tested compounds allows that such these reversible inhibitors display a lower risk of side effects than irreversible inhibitors. As a result, enzyme kinetics studies have shown the biological importance of compounds **3d** and **3h** due to their mixed type forces, unlike irreversible enzyme inhibitors.

### 2.4. Molecular Docking Studies

Docking studies were performed in order to gain more insight into the binding mode of compounds **3d**, **3h**, **3l** and **3p** to AChE enzyme. Studies were carried out by using the X-ray crystal structure of *Homo sapiens* AChE (*h*AChE PDB ID:4EY7) [36] obtained from Protein Data Bank server (www.pdb.org).

**Figure 3:**
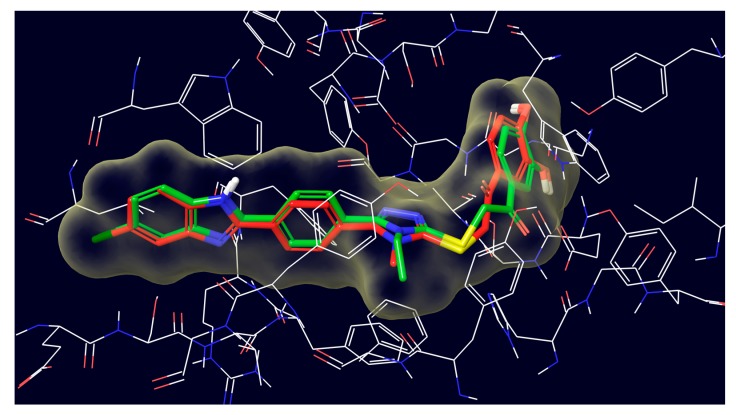
The superimposition pose of compounds **3d** and **3h** (red and green colored, respectively) in the enzyme active site (AChE PDB Code: 4EY7).

**Figure 4:**
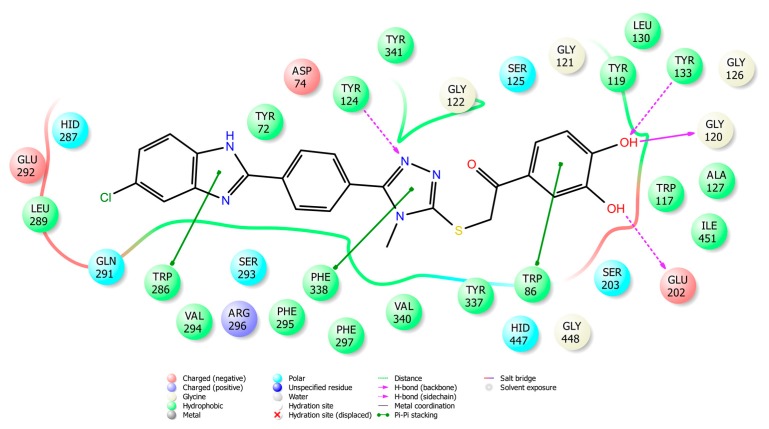
Two-dimensional interaction mode of compound **3d** in the enzyme active site of AChE.

**Figure 5:**
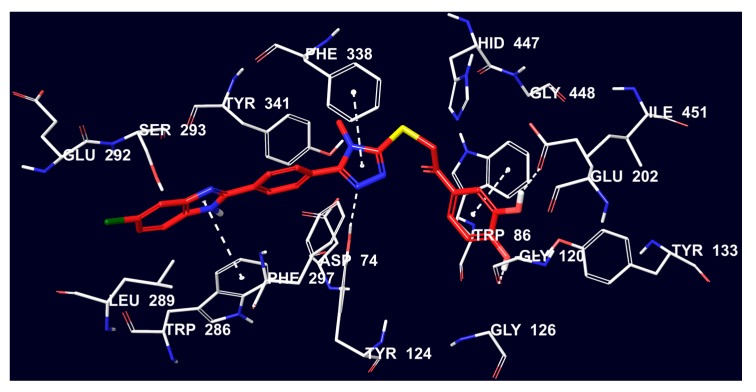
The interacting mode of compound **3d** in the active region of AChE. The inhibitor, colored with red, and the important residues, colored with white, in the active site of the enzyme are presented by tube model.

**Figure 6:**
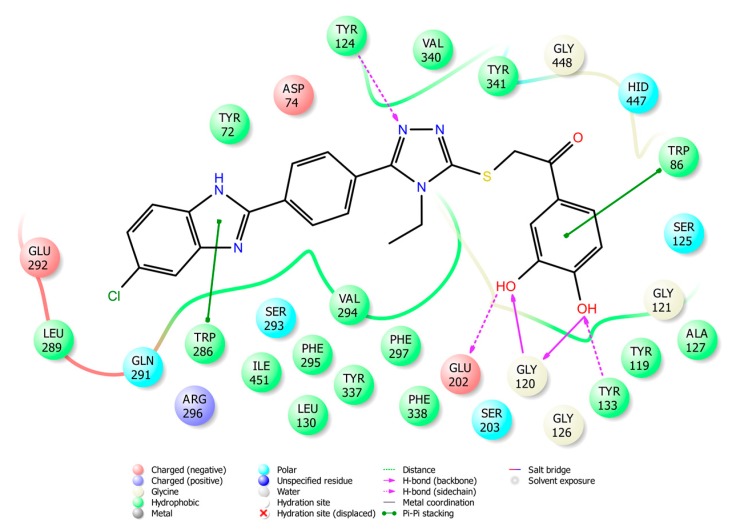
Two-dimensional interaction mode of compound **3h** in the enzyme active site of AChE.

**Figure 7:**
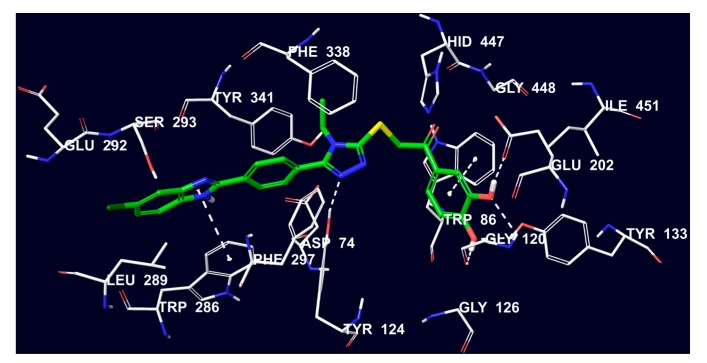
The interacting mode of compound **3h** in the active region of AChE. The inhibitor, colored with green, and the important residues, colored with white, in the active site of the enzyme are presented by tube model.

The docking poses of the compounds **3d** and **3h** are presented in Figure 3, Figure 4, Figure 5, Figure 6 and Figure 7, whereas the poses of donepezil, compounds **3l** and **3p** are presented in Supporting Information Figures S1–S5. As seen from Figure S1 it is known that donepezil could bind strongly to the enzyme active region owing to its dual binding sites. The 5,6-dimethoxyindanone moiety binds to the peripheral anionic site (PAS) of the enzyme active region by interacting with the Trp286 and Phe295 amino acid residues. The rest of the structure, the benzylpiperidine group, binds to the catalytic anionic site (CAS) by interacting with Trp86. Especially as known that the interaction with Trp286 is essential for binding to PAS, whereas interaction with Trp86 is a key point of locating into CAS. When analyzing the docking results of compounds **3d**, **3h**, **3l** and **3p** (Supporting Information Figures S2–S5, Figure 3, Figure 4, Figure 5, Figure 6 and Figure 7), it can be seen that these compounds settle into the CAS and PAS regions of enzyme active site in a similar position as donepezil. Lipophilic parts of the compounds consist of fluoro/chloro-benzimidazole ring, while 1-(3,4-dihydroxyphenyl)-2-((4-methyl/ethyl-5-substitued-4*H*-1,2,4-triazol-3-yl)thio)ethan-1-ones are constituted by a polar basic center. The docking poses indicate that the PAS region of AChE interacts with lipophilic groups in the structure by interacting with Trp286, whereas the polar and basic groups bind to the CAS region of the enzyme by interacting with the Trp86 amino acid residue.

Two and three-dimensional binding modes of compounds **3d** and **3h** are given in Figure 4, Figure 5, Figure 6 and Figure 7. According to the docking poses, these compounds have six common interactions. The 5(6)-chloro-benzimidazole ring interacts with the phenyl of the Trp286 indole by a π–π interaction. The nitrogen atom of triazole forms a hydrogen bond with the hydroxy of Tyr124. The 3,4-dihydroxyphenyl ring creates a π–π interaction with the indole phenyl of Trp86. This dihydroxy substituent is also essential for polar interactions. The hydroxy group at the 4 position of the phenyl ring forms two hydrogen bonds with the carbonyl of Gly120 by acting as a hydrogen donor and the hydroxy of Tyr133 by acting as a hydrogen acceptor. The other hydroxy moiety, at the 3 position of the phenyl, creates another hydrogen bond with the carbonyl of Glu202.

The main difference between compounds **3d** and **3h** that they carry methyl and ethyl moieties, respectively, at the 4 position of the triazole ring. This suggests that the additional interaction of these compounds is caused by small differences in the conformational direction. Compound **3d** has a π-π interaction between the triazole ring and the phenyl of Phe338. On the other hand, the hydroxy group at the 3 position of the phenyl ring forms an additional hydrogen bond with the carbonyl of Gly120 for compound **3h**. Also, it is seen that the change of methyl at the 4 position of the triazole ring to an ethyl group does not cause a significant difference in the enzyme inhibition profile of these compounds. Furthermore, the substituents on the phenyl ring at the end of the structure are responsible for binding to the enzyme active site strongly and thus producing differences in terms of enzyme inhibition potency. It can be suggested that the presence of groups capable of hydrogen bonding at the 3 and 4 positions of the phenyl ring allow the compounds to be placed in stronger and more convenient positions. For compounds **3d** and **3h**, the hydroxy moiety at the 3 and 4 positions provide hydrogen bonds and therefore this situation enables them to show a more compatible binding potential than other compounds in the series. All these interactions show that compounds **3d** and **3h** could bind to AChE enzyme active region in a similar and proper conformation by similar interactions. Besides, it can be explained with the help of docking studies that these compounds display similar inhibition profiles on AChE enzyme with IC_50_ values of 31.9 ± 1.0 nM and 29.5 ± 1.2 nM, respectively.

The structurally fundamental difference of compounds **3l** and **3p** from compounds **3d** and **3h** is that the chlorine substituent on the benzimidazole ring is replaced by a fluorine atom. Thus, it may be expected that compounds **3l** and **3p** exhibit similar binding properties such as compounds **3d** and **3h** and demonstrate similar activity on AChE enzyme. However, according to the enzyme inhibition results, compounds **3l** and **3p** are the most potent derivatives after compounds **3d** and **3h** in the series. Compound **3l** shows an AChE enzyme inhibition profile with an IC_50_ value of 169.4 ± 7.2 nM, however, compound **3p** has an IC_50_ value of 139.9 ± 5.1 nM on AChE enzyme. The docking results help explain this difference. The poses of compounds **3l** and **3p** are presented in Supporting Information Figures S2–S5.

By comparing Figure 4 and Figure 5 and Supporting Information Figures S2 and S3, it can be seen that compound **3l** shows the same five interactions as compound **3d**, but compound **3l** does not have the interaction between the triazole ring and the phenyl of Phe338. Also, the hydrogen bond between the hydroxy moiety at the 4 position of the phenyl ring anthe d hydroxy of Tyr133 could not be seen in the compound **3l**.

The interaction differences observed between compounds **3d** and **3l** are also observed between compounds **3h** and **3p** (Figure 6 and Figure 7 and Supporting Information Figures S4 and S5). For compound **3p**, the nitrogen atom of the triazole ring cannot form a hydrogen bond with the Tyr124 amino acid residue. Moreover, the hydroxy group at the 4 position of the phenyl ring does not form a hydrogen bond with hydroxy of Tyr133. All these losses of interactions mentioned above may explain why compounds **3l** and **3p** exhibit a lower inhibition profile than compounds **3d** and **3f**. Besides, it can be suggested that the presence of a fluorine atom instead of a chlorine on the benzimidazole ring cause distinctness in terms of conformational directions because the chlorine atom has a larger atomic volume in comparison with a fluorine atom. These conformational differences caused the interaction losses observed in compounds **3l** and **3p**.

It can be understood by looking a the enzyme inhibition assay that none of the synthesized compounds display significant activity against BChE enzyme. In order to illuminate this situation *in silico*, docking studies were performed for compound **3h** by using the X-ray crystal structure of *Homo sapiens* BChE (*h*BChE PDB ID:4BDS) obtained from the Protein Data Bank server (www.pdb.org). The docking pose of compound **3h** on BChE enzyme is presented in Supporting Information Figure S6. As can be seen from this figure, compound **3h** could not bind to the BChE enzyme active region properly. This compound shows two interactions related only to the phenyl ring at the end of the structure. Namely, it is unable to display a general interaction profile with its whole chemical structure. The phenyl ring interacts with the imidazole of Hid438. Also, a hydrogen bond is observed between the hydroxy group at the 4 position of the phenyl ring and the carbonyl of Glu197. Consequently, it can be seen that compound **3h**, selected as the example for the docking study on BChE enzyme, could not settle in the BChE enzyme active site in a strong and proper position as in AChE enzyme. The reason for the low BChe activity of the synthesized compounds can be explained as the inability of the general chemical skeleton to locate into the active site.

## 3. Materials and Methods

### 3.1. Chemistry

All chemicals used in the study were purchased either from Sigma-Aldrich (St. Louis, MO, USA) or Merck KGaA (Darmstadt, Germany), and used without further chemical or biological purification. Microwave syntheses were realized by using a Monowave 300 high-performance microwave reactor (Anton-Paar, Graz, Austria). Melting points of synthesized compounds were determined by using an automatic melting point determination system (MP90 series, Mettler-Toledo, Ohio, OH, USA) and were uncorrected. ^1^H-NMR and ^13^C-NMR spectra were recorded in DMSO-*d_6_* by a Bruker digital FT-NMR spectrometer (Bruker Bioscience, MA, USA) at 300 MHz and 75 MHz, respectively. High resolution mass spectrometric studies were performed using an LCMS-IT-TOF system (Shimadzu, Kyoto, Japan). Chemical purities of the compounds were checked by classical TLC applications performed on silica gel 60 F254 (Merck KGaA); LCMS-IT-TOF chromatograms were also used for the same purpose.

#### 3.1.1. 5(6)-Chloro/fluoro-2-((4-methylcarboxylate)phenyl)-1*H*-benzimidazole

In a microwave synthesis reactor vial (Anton-Paar, Graz, Austria, 30 mL), equipped with a magnetic stirrer, methyl-4-formyl benzoate (2.4 g, 0.015 mol) in DMF (10 mL) and sodium bisulfite (2.85 g, 0.015 mol) were heated under conditions of 240 °C and 10 bar for 5 min. The mixture was cooled, 5-chloro/fluoro-1,2-phenylenediamine (0.015 mol) was added into the vial and then reaction mixture was kept under same reaction conditions in the microwave reactor for 5 min. The mixture was cooled and poured into iced-water. The obtained solid was filtered, washed with water, dried and recrystallized from ethanol [33,34].

#### 3.1.2. 4-(5(6)-Chloro/fluoro-1*H*-benzimidazol-2-yl)benzohydrazide 

5(6)-Chloro/fluoro-2-((4-methylcarboxy)phenyl)-1*H*-benzimidazole (0.01 mol) and excess of hydrazine hydrate (5 mL) in ethanol (15 mL) was heated under the conditions of 150 °C and 10 bar for 10 min in the microwave synthesis reactor (Anton-Paar, Graz, Austria). The mixture was cooled and poured into iced-water. The obtained solid was filtered, washed with water, dried and recrystallized from ethanol [33,34].

#### 3.1.3. *N*-methyl/ethyl-2-[4-(5(6)-chloro/fluoro-1*H*-benzimidazol-2-yl)benzoyl]hydrazine-1-carbothioamide 

4-(5(6)-Chloro/fluoro-1*H*-benzimidazol-2-yl)benzohydrazide (0.01 mol) and methyl isothiocyanate or ethyl isothiocyanate (0.012 mol) in ethanol were refluxed for 2 h. The precipitated product was filtered, washed with ethanol and dried [34].

#### 3.1.4. 4-Methyl/Ethyl-5[4-5(6)-chloro/fluoro-1*H*-benzimidazol-2-yl)phenyl)-4*H*-1,2,4-triazole-3-thiol (**1a**–**d**)

*N*-methyl/ethyl-2[4-(5(6)-chloro/fluoro-1*H*-benzimidazol-2-yl)benzoyl]hydrazine-1-carbothio-amide (0.001 mol) in ethanol was refluxed under stirring for 2 h in the presence of NaOH (0.012 mol). After completion of reaction, the solution was acidified with HCl 37%, the precipitate was filtered, washed with water, dried and then recrystallized from ethanol [34].

#### 3.1.5. 2-(4-(4-Methyl/ethyl-5-(2-(substitutedphenyl)-2-oxo-ethylthio)-4*H*-1,2,4-triazol-3yl)-phenyl)-5(6)-chloro/fluoro-1*H*-benzimidazoles **3a**–**p**

A solution of **1a** or **1b** (0.001 mol) in acetone (10 mL), an appropriate substituted 2-bromoacetophenone derivative (0.001 mol) and potassium carbonate (0.138 g, 0.001 mol) were refluxed at 40 °C for 12 h. The solvent was evaporated, residue was washed with water, dried and recrystallized from ethanol. The mass spectra of the compounds (3**a**–**p**) are available in the Supplementary Materials.

*2-(4-(4-Methyl-5-(2-(4-cyanophenyl)-2-oxo-ethylthio)-4H-1,2,4-triazol-3yl)-phenyl)-5(6)-chloro-1H-benz-imidazole* (**3a**). Yield: 84%. M.p. 269.5–271.8 °C. ^1^H-NMR: δ = 3.72 (3H, s, –CH_3_), 4.98 (2H, s, –CH_2_–), 7.25 (1H, t, *J* = 8.5 Hz, benzimidazole C–H), 7.57 (1H, d, *J* = 8.5 Hz, benzimidazole C-H), 7.72 (1H, br.s., benzimidazole C–H), 7.91 (2H, d, *J* = 8.5 Hz, 4-cyanophenyl C–H), 8.05 (2H, d, *J* = 8.4 Hz, 1,4-disubstituted benzene C–H), 8.18 (2H, d, *J* = 8.5 Hz, 4-cyanophenyl C–H), 8.33 (2H, d, *J* = 8.4 Hz, 1,4-disubstituted benzene C–H), 13.29 (1H, s, benzimidazole –NH). ^13^C-NMR: δ (ppm): 32.50, 41.19, 111.67, 113.30, 116.02, 118.55, 118.90, 120.78, 122.78, 123.45, 127.41, 128.77, 129.25, 129.53, 131.33, 133.29, 139.01, 145.19, 150.88, 155.29, 193.45. [M + H]^+^ calcd for C_25_H_17_ClN_6_OS: 485.0930; found: 485.0946.

*2-(4-(4-Methyl-5-(2-(4-bromophenyl)-2-oxo-ethylthio)-4H-1,2,4-triazol-3yl)-phenyl)-5(6)-chloro-1H-benz-imidazole* (**3b**). Yield: 82%. M.p. 279.1–281.4 °C. ^1^H-NMR: δ = 3.72 (3H, s, –CH_3_), 4.93 (2H, s, –CH_2_–), 7.25 (1H, d, *J* = 8.1 Hz, benzimidazole C–H), 7.61–7.75 (2H, m, benzimidazole C–H), 7.77 (2H, d, *J* = 8.5 Hz, 4-bromophenyl C–H), 7.91 (2H, d, *J* = 8.5 Hz, 1,4-disubstituted benzene C–H), 7.97 (2H, d, *J* = 8.6 Hz, 4-bromophenyl C–H), 8.33 (2H, d, *J* = 8.5 Hz, 1,4-disubstituted benzene C–H), 13.27 (1H, s, benzimidazole –NH). ^13^C-NMR: δ = 32.51, 41.11, 111.64, 113.30, 118.90, 120.80, 122.92, 123.14, 126.81, 127.41, 128.39, 128.78, 129, 130.91, 131.33, 132.35, 134.76, 151.01, 155.25, 193.20. [M + H]^+^ calcd for C_24_H_17_BrClN_5_OS: 538.0060; found: 538.0098.

*2-(4-(4-Methyl-5-(2-(4-methylphenyl)-2-oxo-ethylthio)-4H-1,2,4-triazol-3yl)-phenyl)-5(6)-chloro-1H-benz-imidazole* (**3c**). Yield: 79%. M.p. 254.9–256.3 °C. ^1^H-NMR: δ = 2.38 (3H, s, CH_3_), 3.71 (3H, s, –CH_3_), 4.91 (2H, s, –CH_2_–), 7.25 (1H, dd, *J* = 8.6–2.0 Hz, benzimidazole C–H), 7.34-7.37 (2H, m, Ar–C–H), 7.62-7.70 (2H, m, Ar–C–H), 7.89–7.94 (4H, m, Ar–C–H), 8.33 (2H, d, *J* = 8.4 Hz, 1,4-disubstituted benzene C–H), 13.32 (1H, s, benzimidazole –NH). ^13^C-NMR: δ = 21.67, 32.48, 41.27, 106.76, 117.24, 123.14, 127.41, 127.81, 128.81, 129.03, 129.25, 129.82, 130.80, 131.30, 133.21, 133.70 144.77, 151.16, 152.23, 155.21, 193.35. [M + H]^+^ calcd for C_25_H_20_ClN_5_OS: 474.1148; found: 474.1150.

*2-(4-(4-Methyl-5-(2-(3,4-dihydroxyphenyl)-2-oxo-ethylthio)-4H-1,2,4-triazol-3yl)-phenyl)-5(6)-chloro-1H-benzimidazole* (**3d**). Yield: 76%. M.p. 261.2–262.8 °C. ^1^H-NMR: δ = 3.71 (3H, s, –CH_3_), 4.80 (2H, s, –CH_2_), 6.81 (1H, d, *J* = 8.0 Hz, dihydroxyphenyl C–H), 7.25 (1H, dd, *J* = 8.6–2.0 Hz, benzimidazole C–H), 7.38-7.45 (2H, m, Ar–C–H), 7.6–7.73 (2H, m, Ar–CH), 7.91 (2H, d, *J* = 8.4 Hz, 1,4-disubstituted benzene C–H), 8.33 (2H, d, *J* = 8.4 Hz, 1,4-disubstituted benzene C–H), 13.26 (1H, s, benzimidazole -NH). ^13^C–NMR: δ = 32.47, 41.09, 114.67, 115.26, 115.62, 122.70, 123.13, 127.16, 127.41, 128.47, 128.85, 129.26, 130.77, 131.30, 138.96, 146.15, 151.41, 152.2, 152.79, 155.18, 191.65. [M + H]^+^ calcd for C_24_H_18_ClN_5_O_3_S: 492.0877; found: 492.0892.

*2-(4-(4-Ethyl-5-(2-(4-cyanophenyl)-2-oxo-ethylthio)-4H-1,2,4-triazol-3yl)-phenyl)-5(6)-chloro-1H-benz-imidazole* (**3e**). Yield: 81%. M.p. 258.7–259.9 °C. ^1^H-NMR: δ = 1.28 (3H, t, *J* = 7.2, –CH_3_), 4.12 (2H, q, *J* = 7.2 Hz, –CH_2_), 5.03 (2H, s, –CH_2_), 7.24 (1H, dd, *J* = 8.6–1.9 Hz, benzimidazole C–H),7.62-7.68 (2H, m, benzimidazole C–H), 7.85 (2H, d, *J* = 8.4 Hz, 4-cyanophenyl C–H), 8.04 (2H, d, *J* = 8.3 Hz, 1,4-disubstituted benzene C–H), 8.19 (2H, d, *J* = 8.4 Hz, 4-cyanophenyl C–H), 8.33 (2H, d, *J* = 8.3 Hz, 1,4-disubstituted benzene C–H), 13.27 (1H, s, benzimidazole –NH). ^13^C-NMR: δ = 15.51, 36.23, 41.15, 116.04, 118.55, 119.28, 123.15, 127.55, 128.90, 129.29, 129.51, 129.83, 131.46, 132.93, 133.29, 139.03, 144.94, 150.39, 152.19, 154.78, 193.31. [M + H]^+^ calcd for C_26_H_19_ClN_6_OS: 499.1092; found: 499.1102.

*2-(4-(4-Ethyl-5-(2-(4-bromophenyl)-2-oxo-ethylthio)-4H-1,2,4-triazol-3yl)-phenyl)-5(6)-chloro-1H-benz-imidazole* (**3f**). Yield: 80%. M.p. 249.3–251.4 °C. ^1^H-NMR: δ = 1.28 (3H, t, *J* = 7.20, –CH_3_), 4.12 (2H, q, *J* = 7.2 Hz, –CH_2_), 4.99 (2H, s, –CH_2_–), 7.26 (1H, dd, *J* = 8.6–2.0 Hz, benzimidazole C-H), 7.63–7.69 (2H, m, benzimidazole C–H), 7.79 (2H, d, *J* = 8.6 Hz, 4-bromophenyl), 7.85 (2H, d, *J* = 8.5 Hz, 1,4-disubstituted benzene C–H), 7.98 (2H, d, *J* = 8.6 Hz, 4-bromophenyl), 8.33 (2H, d, *J* = 8.5 Hz, 1,4-disubstituted benzene C–H). ^13^C-NMR: δ = 15.52, 34.01, 41.06, 116.52, 120.40, 123.20, 127.23, 127.56, 128.39, 128.98, 129.33, 130.92, 131.40, 131.89, 132.38, 134.82, 139.48, 150.50, 152.19, 154.73, 193.10. [M + H]^+^ calcd for C_25_H_19_BrClN_5_OS: 552.0249; found: 552.0255.

*2-(4-(4-Ethyl-5-(2-(4-methylphenyl)-2-oxo-ethylthio)-4H-1,2,4-triazol-3yl)-phenyl)-5(6)-chloro-1H-benz-imidazole* (**3g**). Yield: 74%. M.p. 247.9–249.6 °C. ^1^H-NMR: δ = 1.28 (3H, t, *J* = 7.2, –CH_3_), 2.43 (3H, s, –CH_3_), 4.11 (2H, q, *J* = 7.2 Hz, –CH_2_), 4.98 (2H, s, –CH_2_–), 7.26 (1H, d, *J* = 8.4 Hz, benzimidazole C–H), 7.37 (2H, d, *J* = 8.2, 4-methylphenyl C–H), 7.60–7.75 (2H, m, benzimidazole C–H), 7.85 (2H, d, *J* = 8.4 Hz, 1,4-disubstituted benzene C–H), 7.95 (2H, d, *J* = 8.2 Hz, 4-methylphenyl C–H), 8.33 (2H, d, *J* = 8.4 Hz, 1,4-disubstituted benzene C–H), 13.28 (1H, s, benzimidazole –NH). ^13^C-NMR: δ = 15.53, 21.68, 33.58, 41.22, 111.68, 113.31, 118.98, 123.48, 127.54, 128.43, 129.03, 129.32, 129.85, 130.59, 131.43, 132.59, 133.27, 144.79, 150.42, 150.66, 154.69, 193.21. [M + H]^+^ calcd for C_26_H_22_ClN_5_OS: 488.1300; found: 488.1306.

*2-(4-(4-Ethyl-5-(2-(3,4-dihydroxyphenyl)-2-oxo-ethylthio)-4H-1,2,4-triazol-3yl)-phenyl)-5(6)-chloro-1H-benzimidazole* (**3h**). Yield: 83%. M.p. 262.3–264.1 °C. ^1^H-NMR: δ = 1.27 (3H, t, *J* = 7.2 Hz, –CH_3_), 4.11 (2H, q, *J* = 7.2 Hz, –CH_2_), 4.88 (2H, s, –CH_2_–), 6.86 (1H, d, *J* = 8.3 Hz, dihydroxyphenyl C–H), 7.25 (1H, dd, *J* = 8.5–1.9 Hz, benzimidazole C–H), 7.40–7.47 (2H, m, dihydroxyphenyl C–H), 7.63–7.69 (2H, m, benzimidazole C–H), 7.85 (2H, d, *J* = 8.3 Hz, 1,4-disubstituted benzene C–H), 8.34 (2H, d, *J* = 8.3 Hz, 1,4-disubstituted benzene C–H). ^13^C-NMR: δ = 15.53, 36.24, 41.08, 111.36, 115.66, 118.96, 120.97, 122.48, 123.17, 125.42, 127.55, 128.29, 129.03, 129.31, 131.43, 133.31, 133.57, 145.86, 150.85, 151.93, 152.23, 154.66, 191.71. [M + H]^+^ calcd for C_25_H_20_ClN_5_O_3_S: 506.1038; found: 506.1048.

*2-(4-(4-Methyl-5-(2-(4-cyanophenyl)-2-oxo-ethylthio)-4H-1,2,4-triazol-3yl)-phenyl)-5(6)-fluoro-1H-benz-imidazole* (**3i**). Yield: 76%. M.p. 260.9–262.7 °C. ^1^H-NMR: δ = 3.72 (3H, s, –CH_3_), 4.98 (2H, s, –CH_2_), 7.08–7.11 (1H, m, benzimidazole C–H), 7.34–7.73 (2H, m, benzimidazole C–H), 7.90 (2H, d, *J* = 8.5 Hz, 4-cyanophenyl C–H), 8.05 (2H, d, *J* = 8.5 Hz, 1,4-disubstituted benzene C–H), 8.19 (2H, d, *J* = 8.5 Hz, 4-cyanophenyl C–H), 8.32 (2H, d, *J* = 8.5 Hz, 1,4-disubstituted benzene C–H), 13.21 (1H, s, benzimidazole –NH). ^13^C-NMR: δ = 32.51, 41.17, 98.31 (d, *J* = 27.3 Hz), 104.95 (d, *J* = 23.3 Hz), 112.66 (d, *J* = 10.9 Hz), 120.44, 127.25 (d, *J* = 8.1 Hz), 128.51, 129.04, 129.25, 131.49, 132.25, 133.31, 135.74, 140.97, 144.75, 150.85, 152.82 (d, *J* = 2.8 Hz), 155.31, 157.95 (d, *J* = 233.5 Hz), 193.47. HRMS (*m*/*z*): [M + H]^+^ calcd for C_25_H_17_FN_6_OS: 469.1227; found: 469.1241.

*2-(4-(4-Methyl-5-(2-(4-bromophenyl)-2-oxo-ethylthio)-4H-1,2,4-triazol-3yl)-phenyl)-5(6)-fluoro-1H-benz-imidazole* (**3j**). Yield: 73%. M.p. 295.8–298.1 °C. ^1^H-NMR: δ = 3.71 (3H, s, –CH_3_), 4.93 (2H, s, –CH_2_–), 7.10 (1H, s, benzimidazole C–H), 7.37–7.56 (2H, m, benzimidazole C–H), 7.79 (2H, d, *J* = 8.2 Hz, 4-bromo-phenyl C–H), 7.90 (2H, d, *J* = 8.0 Hz, 1,4-disubstituted benzene C–H), 7.97 (2H, d, *J* = 8.2 Hz, 4-bromophenyl C–H), 8.32 (2H, d, *J* = 8.0 Hz, 1,4-disubstituted benzene C–H), 13.21 (1H, s, benzimidazole –NH). ^13^C-NMR: δ = 32.51, 41.09, 98.45 (d, *J* = 26.8 Hz), 104.82 (d, *J* = 24.3 Hz), 113.42 (d, *J* = 11.4 Hz), 121.52, 127.23 (d, *J* = 8.9 Hz), 128.93, 129.27, 130.94, 132.37, 134.5, 137.4, 140.39, 150.25, 152.73 (d, *J* = 2.6 Hz), 153.21, 155.70, 161.03 (d, *J* = 228.6 Hz), 193.56. HRMS (*m*/*z*): [M + H]^+^ calcd for C_24_H_17_BrFN_5_OS: 522.0374; found: 522.0394.

*2-(4-(4-Methyl-5-(2-(4-methylphenyl)-2-oxo-ethylthio)-4H-1,2,4-triazol-3yl)-phenyl)-5(6)-fluoro-1H-benz-imidazole* (**3k**). Yield: 71%. M.p. 284.3–286.9 °C. ^1^H-NMR: δ = 2.39 (3H, s, CH_3_), 3.71 (3H, s, –CH_3_), 4.92 (2H, s, –CH_2_–), 7.07–7.14 (1H, m, benzimidazole C–H), 7.36 (2H, d, *J* = 8.1 Hz, 4-methylphenyl C–H), 7.42-7.46 (1H, m, Ar–C–H), 7.62–7.66 (1H, m, Ar–CH C–H), 7.89–7.95 (4H, Ar–C–H), 8.33 (2H, d, *J* = 8.4 Hz, 1,4-disubstituted benzene C–H). ^13^C-NMR: δ = 21.67, 32.48, 41.25, 99.46 (d, *J* = 25.3 Hz), 101.78, 104.85 (d, *J* = 21.3 Hz), 111.29 (d, *J*= 10.2 Hz), 116.37, 120.44, 127.52 (d, *J* = 9.6 Hz), 128.78, 129.05, 129.28, 129.83, 131.23, 133.22, 144.78, 151.18 (d, *J* = 2.9 Hz), 155.20, 159.37 (d, *J* = 236.8 Hz), 193.36. HRMS (*m*/*z*): [M + H]^+^ calcd for C_25_H_20_FN_5_OS: 458.1437; found: 458.1445.

*2-(4-(4-Methyl-5-(2-(3,4-dihydroxyphenyl)-2-oxo-ethylthio)-4H-1,2,4-triazol-3yl)-phenyl)-5(6)-fluoro-1H-benzimidazole* (**3l**). Yield: 77%. M.p. 277.8–279.2 °C. ^1^H-NMR: δ = 3.71 (3H, s, –CH_3_), 4.82 (2H, s, –CH_2_–), 6.85 (1H, d, *J* = 8.2 Hz, dihydroxyphenyl C–H), 7.09 (1H, br.s., Ar–C–H), 7.39–7.56 (4H, m, Ar–C–H), 7.90 (2H, d, *J* = 8.2 Hz, 1,4-disubstituted benzene C–H), 8.32 (2H, d, *J* = 8.2 Hz, 1,4-disubstituted benzene C–H), 13.22 (1H, s, denzimidazole –NH). ^13^C-NMR: δ = 32.46, 41.07, 98.33 (d, *J* = 28.9 Hz), 104.99 (d, *J* = 30.7 Hz), 110.81, 111.49 (d, *J* = 11.4 Hz), 112.75, 115.64, 120.41, 122.51, 127.38 (d, *J* = 9.4 Hz), 128.72, 129.26, 131.48, 132.21, 145.83, 150.75, 151.59 (d, *J* = 2.6 Hz), 153.62, 155.21, 162.78 (d, *J* = 236.8 Hz), 191.88. HRMS (*m*/*z*): [M + H]^+^ calcd for C_24_H_18_FN_5_O_3_S: 476.1176; found: 476.1187.

*2-(4-(4-Ethyl-5-(2-(4-cyanophenyl)-2-oxo-ethylthio)-4H-1,2,4-triazol-3yl)-phenyl)-5(6)-fluoro-1H-benz-imidazole* (**3m**). Yield: 70%. M.p. 225.4–227.1 °C. ^1^H-NMR: δ = 1.27 (3H, t, *J* = 7.2 Hz,–CH_3_), 4.11 (2H, q, *J* = 7.2 Hz, –CH_2_), 5.03 (2H, s, –CH_2_–), 7.10 (1H, t, *J* = 8.5 Hz, benzimidazole C–H), 7.43 (1H, br. s, benzimidazole C–H), 7.62 (1H, br.s., benzimidazole C–H), 7.83 (2H, d, *J* = 8.4 Hz, 4-cyanophenyl C–H), 8.05 (2H, d, *J* = 8.4, 1,4-disubstituted benzene C–H), 8.19 (2H, d, *J* = 8.4, 4-cyanophenyl C–H), 8.31 (2H, d, *J* = 8.4 Hz, 1,4-disubstituted benzene C–H), 13.24 (1H, s, benzimidazole –NH). ^13^C-NMR: δ = 15.49, 40.30, 41.08, 98.19 (d, *J* = 25.2 Hz), 104.57 (d, *J* = 31.1 Hz), 111.55 (d, *J* = 11.3 Hz), 116.02, 118.56, 127.40, 128.71, 129.42 (d, *J* = 14.9 Hz), 131.60, 133.31, 134.81, 139.03, 142.18, 150.39, 152.18, 152.75 (d, *J*= 2.8 Hz), 154.81, 162.24 (d, *J* = 228.4 Hz), 193.33. HRMS (*m*/*z*): [M + H]^+^ calcd for C_26_H_19_N_5_FN_6_OS: 483.1386; found: 483.1398.

*2-(4-(4-Ethyl-5-(2-(4-bromophenyl)-2-oxo-ethylthio)-4H-1,2,4-triazol-3yl)-phenyl)-5(6)-fluoro-1H-benz-imidazole* (**3n**). Yield: 73%. M.p. 265.8–267.9 °C. ^1^H-NMR: δ = 1.28 (3H, t, *J* = 7.2 Hz, –CH3), 4.11 (2H, q, *J* = 7.2 Hz, –CH_2_), 4.99 (2H, s, –CH_2_–), 7.06–7.13 (1H, m, benzimidazole C–H), 7.43 (1H, d, *J* = 8.5 Hz, benzimidazole C–H), 7.61-7.66 (1H, m, benzimidazole C–H), 7.79 (2H, d, *J* = 8.5 Hz, 4-bromophenyl C–H), 7.84 (2H, d, *J* = 8.3 Hz, 1,4-disubstituted benzene C–H), 7.98 (2H, d, *J* = 8.5 Hz, 4-bromophenyl C–H), 8.32 (2H, d, *J* = 8.3 Hz, 1,4-disubstituted benzene C–H), 13.20 (1H, s, benzimidazole –NH). ^13^C-NMR: δ = 15.53, 40.80, 48.13, 99.45 (d, *J* = 24.8 Hz), 105.64 (d, *J* = 22.4 Hz), 111.25 (d, *J* = 11.2 Hz), 124.32, 127.40 (d, *J* = 9.2 Hz), 128.39, 128.80, 129.31, 130.92, 132.38, 134.81, 139.13, 150.47, 151.60 (d, *J* = 2.4 Hz), 152.15, 154.76, 159.28 (d, *J* = 234.1 Hz), 193.09. HRMS (*m*/*z*): [M + H]^+^ calcd for C_25_H_19_BrFN_5_OS: 536.0537; found: 536.0550.

*2-(4-(4-Ethyl-5-(2-(4-methylphenyl)-2-oxo-ethylthio)-4H-1,2,4-triazol-3yl)-phenyl)-5(6)-fluoro-1H-benz-imidazole* (**3o**). Yield: 82%. M.p. 260.6–262.2 °C. ^1^H-NMR: δ = 1.28 (3H, t, *J* = 7.1 Hz,–CH_3_), 2.39 (3H, s, CH_3_), 4.10 (2H, q, *J* = 7.1 Hz,–CH_2_), 4.98 (2H, s, –CH_2_–), 7.10 (1H, t, *J* = 8.5 Hz, benzimidazole C–H), 7.37 (2H, d, *J* = 7.7 Hz, 4-methylphenyl C–H), 7.42 (1H, br.s, benzimidazole C–H), 7.63 (1H, br.s, benzimidazole C–H), 7.84 (2H, d, *J* = 8.1 Hz, 1,4-disubstituted benzene C–H), 7.95 (2H, d, *J* = 7.8 Hz, 4-methylphenyl C–H), 8.32 (2H, d, *J* = 8.1 Hz, 1,4-disubstituted benzene C–H), 13.23 (1H, s, benzimidazole –NH). ^13^C-NMR: δ = 15.53, 21.68, 36.28, 41.21, 99.63 (d, *J* = 27.6 Hz), 104.32 (d, *J* = 24.8 Hz), 111.52 (d, *J* = 11.6 Hz), 123.46, 127.39 (d, *J* = 10.2 Hz), 128.82, 129.03, 129.30, 129.84, 131.62, 133.26, 144.79, 150.64 (d, *J* = 2.4 Hz), 152.72, 154.72, 159.24 (d, *J* = 234.7 Hz), 193.21. HRMS (*m*/*z*): [M + H]^+^ calcd for C_26_H_22_FN_5_OS: 472.1602; found: 472.1604.

*2-(4-(4-Ethyl-5-(2-(3,4-dihydroxyphenyl)-2-oxo-ethylthio)-4H-1,2,4-triazol-3yl)-phenyl)-5(6)-fluoro-1H-benzimidazole* (**3p**). Yield: 81%. M.p. 243.9–245.4 °C. ^1^H-NMR: δ = 1.26 (3H, t, *J* = 7.2 Hz,–CH_3_), 4.10 (2H, q, *J* = 7.2 Hz,–CH_2_), 4.77 (2H, s, –CH_2_), 6.48 (1H, d, *J* = 8.4 Hz, dihydroxyphenyl C–H), 7.04–7.11 (1H, m., benzimidazole C–H), 7.20 (1H, d, *J* = 2.3 Hz, dihydroxyphenyl C–H), 7.34 (1H, dd, *J* = 8.4–2.3 Hz, dihydroxyphenyl C–H), 7.42 (1H, dd, *J* = 9.5–2.4 Hz, benzimidazole C–H), 7.60–7.64 (1H, m, Ar–C–H), 7.83 (2H, d, *J* = 8.4 Hz, 1,4-disubstituted benzene C–H), 8.35 (2H, d, *J* = 8.4 Hz, 1,4-disubstituted benzene C–H). ^13^C-NMR: δ = 15.56, 36.04, 41.16, 101.79 (d, *J* = 30.8 Hz), 110.90 (d, *J* = 24.8 Hz), 111.84, 116.50 (d, *J* = 12.3 Hz), 121.39, 122.55, 124.21, 128.86, 129.35 (d, *J* = 8.9 Hz), 130.61, 131.75, 134.81, 139.16, 147.72, 151.16 (d, *J* = 3.9 Hz), 152.40, 154.65, 159.21 (d, *J* = 241.6 Hz), 160.87, 189.78. HRMS (*m*/*z*): [M + H]^+^ calcd for C_25_H_20_FN_5_O_3_S: 490.1328; found: 490.1344.

### 3.2. Anticholinesterase Activity Assay

The inhibitory activities of the compounds against AChE and BChE were determined in 96-well plates by modified Ellman’s method as defined in our previous studies [17,37,38,39]. Donepezil and tacrine were used as reference drugs. A robotic system, Biotek Precision XS (Winooski, VT, USA), was used for all pipetting processes in the enzyme inhibition assay. Measurements of the percentage inhibition were carried out at 412 nm by using a BioTek-Synergy H1 microplate reader (Winooski, VT, USA). Inhibition potencies of synthesized compounds and IC_50_ of selected derivatives were calculated as reported previously [17,37,38,39].

### 3.3. Kinetic Studies of Enzyme Inhibition

Enzyme kinetic studies were performed for compounds **3d** and **3h** in order to determine the inhibition type on AChE. Enzyme kinetic assay was carried out using Ellman’s method according to the previous studies reported by our research group [17,37,38,39].

### 3.4. Molecular Docking Studies

In order to determine the binding modes of compounds **3d** and **3h** on AChE enzyme active site, docking studies were performed by using in silico procedure. X-ray crystal structure of AChE (PDB ID: 4EY7) [34] was retrieved from the Protein Data Bank server (www.pdb.org). The docking procedure was applied as described in our previous works [17,37,38,39].

## 4. Conclusions

AChE and BuChE are enzymes which play an important role in memory and cognition. They catalyse the hydrolysis of acetylcholine causing a loss of communication between nerve cells. This leads to a loss of brain function and causes AD. Pharmaceutical research has thus been focusing on cholinesterase inhibitors as treatments for cognitive disorders. This study aimed to design and synthesized benzimidazole-triazole derivatives and tested their activity against AChE and BuChE. Compounds **3d** and **3h** showed good AChE inhibition, but poor selectivity for BChE. In order to clarify the type of inhibition and their in silico properties, enzyme kinetics and binding studies were carried out for these compounds. These compounds were assessed as reversible and mixed-type AChE enzyme inhibitors. Also, it was shown that compounds **3d** and **3h** could bind to the AChE enzyme active site in a similar manner and proper positions. Consequently, this study presents important results for developing new agents for neurodegenerative diseases such as Alzheimer’s diseases.

## Figures and Tables

**Table:** 

Compounds	R_1_	R_2_	R_3_	R_4_	Yield (%)
**1a**	–Cl	–CH_3_	-	-	81
**1b**	–Cl	–C_2_H_5_	-	-	78
**1d**	–F	–CH_3_	-	-	77
**1c**	–F	–C_2_H_5_	-	-	75
**3a**	–Cl	–CH_3_	–H	–CN	84
**3b**	–Cl	–CH_3_	–H	–Br	82
**3c**	–Cl	–CH_3_	–H	–CH_3_	79
**3d**	–Cl	–CH_3_	–OH	–OH	76
**3e**	–Cl	–C_2_H_5_	–H	–CN	81
**3f**	–Cl	–C_2_H_5_	–H	–Br	80
**3g**	–Cl	–C_2_H_5_	–H	–CH_3_	74
**3h**	–Cl	–C_2_H_5_	–OH	–OH	83
**3i**	–F	–CH_3_	–H	–CN	76
**3j**	–F	–CH_3_	–H	–Br	73
**3k**	–F	–CH_3_	–H	–CH_3_	71
**3l**	–F	–CH_3_	–OH	–OH	77
**3m**	–F	–C_2_H_5_	–H	–CN	70
**3n**	–F	–C_2_H_5_	–H	–Br	73
**3o**	–F	–C_2_H_5_	–H	–CH_3_	82
**3p**	–F	–C_2_H_5_	–OH	–OH	81

**Table 1:** % Inhibition of all compounds **5a**–**p** against AChE and BChE enzymes.

Compounds	% Inhibition			
	AChE		BChE	
	10^−3^ M	10^−4^ M	10^−3^ M	10^−4^ M
**3a**	68.18 ± 1.05	47.26 ± 0.62	40.28 ± 0.98	25.13 ± 0.56
**3b**	44.20 ± 0.87	26.18 ± 0.63	28.15 ± 0.97	18.42 ± 0.88
**3c**	39.18 ± 0.71	17.89 ± 0.54	26.11 ± 0.74	20.21 ± 0.61
**3d**	98.55 ± 1.17	93.99 ± 1.10	51.19 ± 0.99	38.12 ± 0.41
**3e**	77.29 ± 1.06	41.22 ± 0.84	38.22 ± 0.63	22.41 ± 0.47
**3f**	47.22 ± 0.67	24.08 ± 0.48	29.15 ± 0.59	17.88 ± 0.40
**3g**	37.20 ± 0.68	18.33 ± 0.38	31.89 ± 0.99	24.75 ± 0.95
**3h**	98.25 ± 1.12	94.55 ± 1.08	61.26 ± 0.90	35.20 ± 0.42
**3i**	61.22 ± 1.02	38.28 ± 0.67	43.51 ± 0.78	18.49 ± 0.50
**3j**	48.51 ± 0.88	18.39 ± 0.60	29.64 ± 0.87	21.44 ± 0.70
**3k**	42.29 ± 0.71	27.88 ± 0.67	36.17 ± 0.93	21.16 ± 0.97
**3l**	94.58 ± 1.74	83.20 ± 1.13	55.43 ± 0.47	29.88 ± 0.38
**3m**	59.03 ± 0.88	39.26 ± 0.46	48.11 ± 0.38	20.66 ± 0.45
**3n**	49.27 ± 0.55	30.21 ± 0.49	27.10 ± 0.53	18.77 ± 0.42
**3o**	28.06 ± 0.33	19.22 ± 0.30	25.99 ± 0.38	20.08 ± 0.46
**3p**	87.26 ± 1.17	81.25 ± 1.88	53.19 ± 0.46	31.44 ± 0.38
**Donepezil**	99.42 ± 1.85	97.26 ± 1.24	-	-
**Tacrine**	-	-	98.52 ± 1.66	95.50 ± 1.74

**Table 2:** IC_50_ values of selected compounds (**3d**, **3h**, **3l** and **3p**) and donepezil, as a reference drug, on AChE enzyme.

Compounds	% Inhibition of AChE					AChE IC_50_ (nM)
	10^−5^ M	10^−6^ M	10^−7^ M	10^−8^ M	10^−9^ M	
**3d**	87.26 ± 1.28	78.85 ± 1.19	67.18 ± 0.92	45.32 ± 0.82	15.75 ± 0.48	31.9 ± 0.1
**3h**	90.23 ± 1.03	81.25 ± 1.08	65.32 ± 0.97	44.28 ± 0.71	17.20 ± 0.58	29.5 ± 1.2
**3l**	73.18 ± 1.17	68.20 ± 0.97	48.11 ± 0.61	32.29 ± 0.58	18.77 ± 0.37	169.4 ± 7.2
**3p**	77.48 ± 1.98	71.62 ± 1.08	47.83 ± 0.97	39.33 ± 0.67	15.47 ± 0.64	139.9 ± 5.1
**Donepezil**	94.11 ± 1.71	90.17 ± 1.02	75.65 ± 1.15	35.74 ± 0.58	17.89 ± 0.42	21.8 ± 0.9

## Supplementary Materials

The following are available online at https://www.mdpi.com/1420-3049/24/5/861/s1.
