# Supplementary material for: Synthesis and AChE-Inhibitory Activity of New Benzimidazole Derivatives
Source: Molecules. 2019 Feb 28;24(5):861. doi: 10.3390/molecules24050861 (PMC6429502; doi:10.3390/molecules24050861)
Supplement: Supplementary file 1 [file molecules-24-00861-s001.pdf]

SUPPORTING INFORMATION

SYNTHESIS AND AChE INHIBITORY ACTIVITY OF NEW BENZIMIDAZOLE DERIVATIVES

Ulviye ACAR ÇEVİK[1,2], Begüm Nurpelin SAĞLIK [1,2], Serkan LEVENT [1,2], Derya OSMANİYE [1,2], Betül KAYA ÇAVUŞOĞLU [1], Yusuf ÖZKAY* [1,2], Zafer Asım KAPLANCIKLI [1]

[1] Department of Pharmaceutical Chemistry, Faculty of Pharmacy, Anadolu Universty, Eskişehir, Turkey
[2] Doping and Narcotic Compounds Analysis Laboratory, Faculty of Pharmacy, Anadolu Universty, Eskişehir, Turkey
* Correspondence: yozkay@anadolu.edu.tr; Tel.: +90-222-335-0580/3603

Academic Editor: name
Received: date; Accepted: date; Published: date

* Corresponding author.

E-mail address:

Tel: +90-222-3350580/ *Fax*: +90-222-3350750.

Address: Anadolu University, Faculty of Pharmacy, Department of Pharmaceutical Chemistry, 26470, Eskişehir, Turkey.

2-(4-(4-Methyl-5-(2-(4-cyanophenyl)-2-oxo-ethylthio)-4H-1,2,4-triazol-3yl)-phenyl)-5(6)-chloro-1H-benzoimidazole (3a)

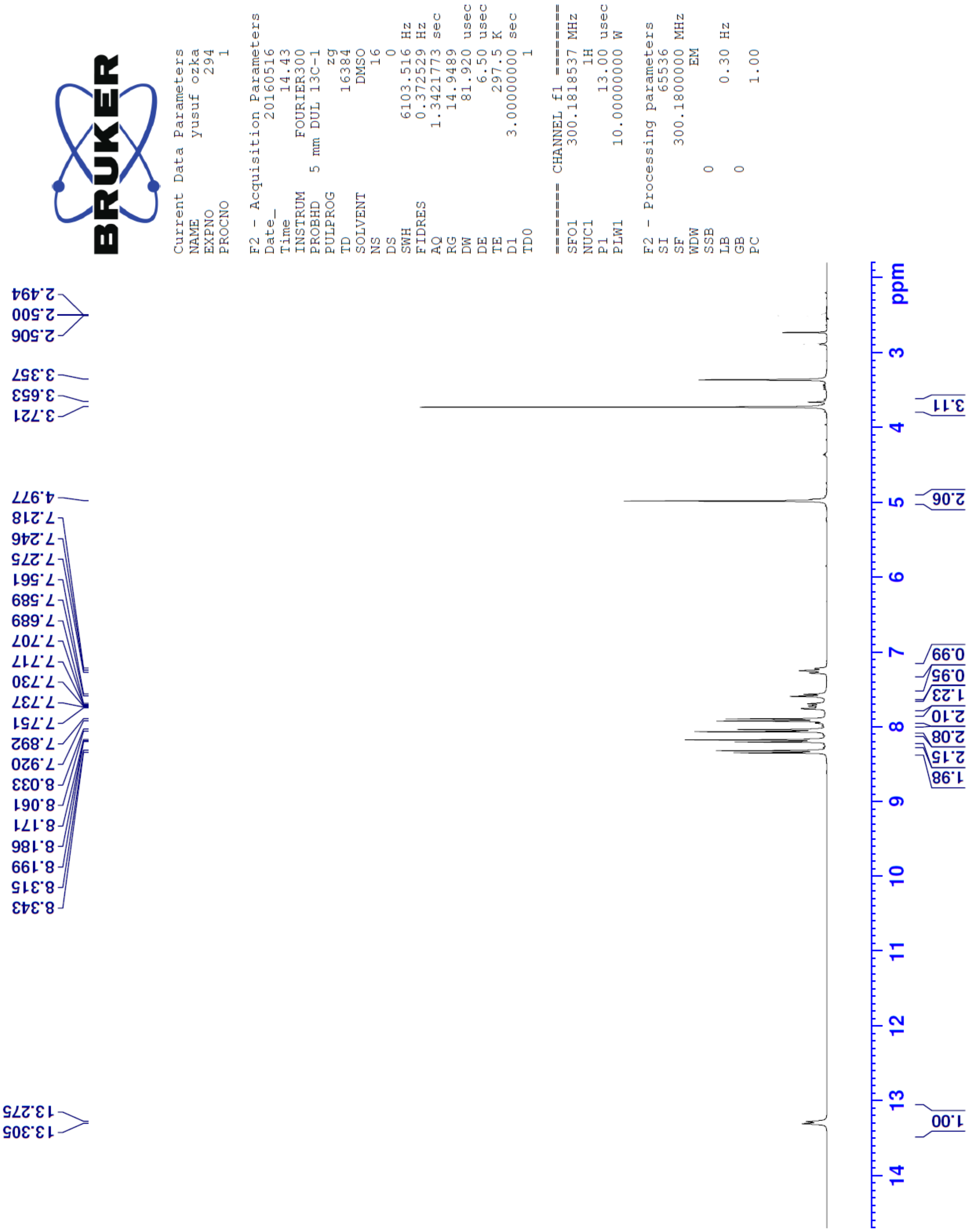

13C, TU-22, DMSO, 298K

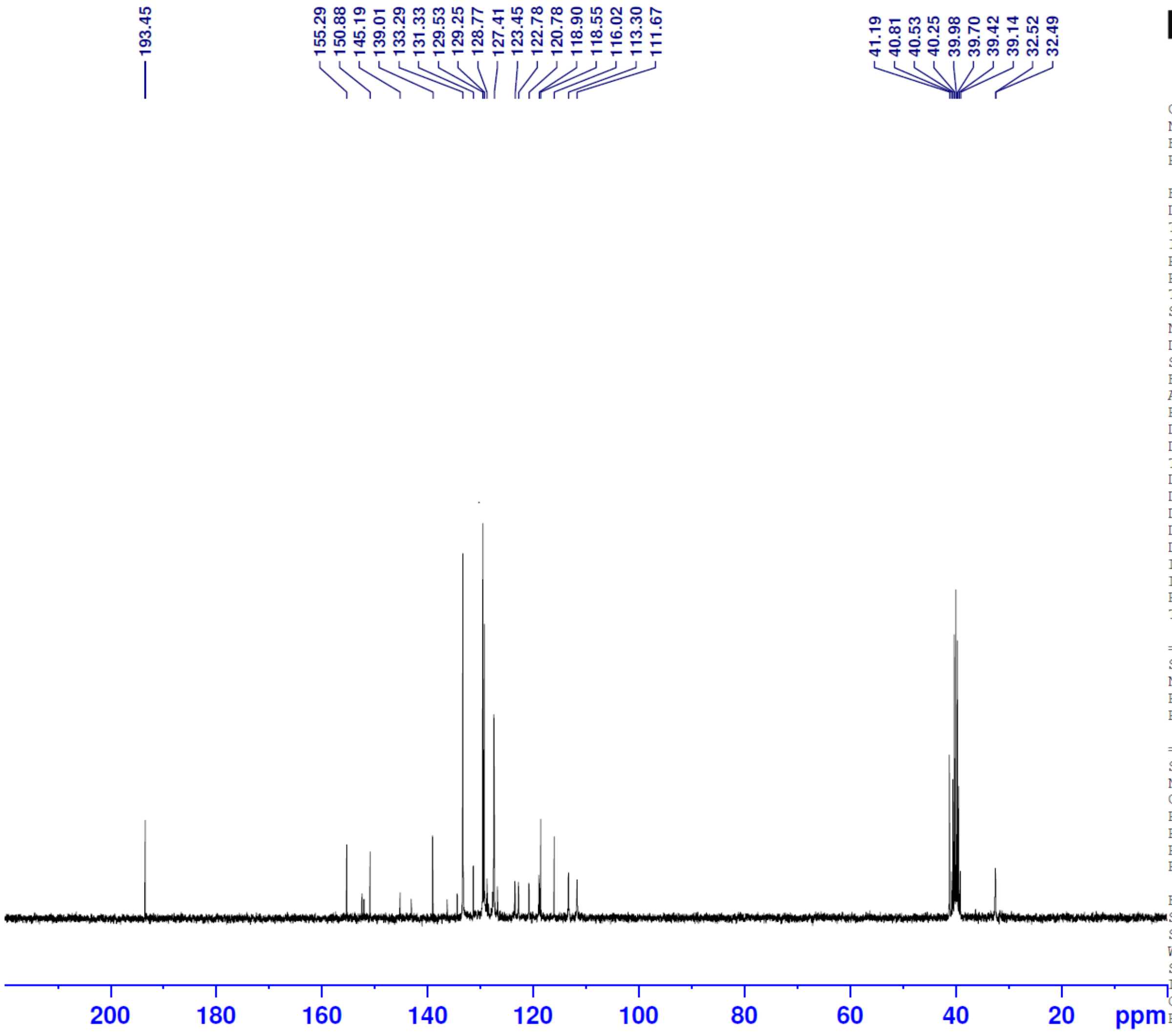

BRUKER

```
Current Data Parameters
NAME             yusuf ozka
EXPNO                   295
PROCNO                    1

F2 - Acquisition Parameters
Date_              20160516
Time                  14.45
INSTRUM          FOURIER300
PROBHD    5 mm DUL 13C-1
PULPROG                zgpg
TD                    32768
SOLVENT                DMSO
NS                     2048
DS                        4
SWH               24414.063 Hz
FIDRES             0.745058 Hz
AQ                0.6710886 sec
RG                  501.187
DW                   20.480 usec
DE                     6.50 usec
TE                    297.9 K
D1               1.00000000 sec
D11              0.03000000 sec
D31              0.00001500 sec
D32              0.89999998 sec
D40              0.00093990 sec
L4                       23
L5                       26
P32                   90.00 usec
TD0                       1

======== CHANNEL f1 ========
SFO1            75.4878687 MHz
NUC1                    13C
P1                    15.00 usec
PLW1            15.00000000 W

======== CHANNEL f2 ========
SFO2           300.1812007 MHz
NUC2                     1H
CPDPRG[2            waltz16
PCPD2                 90.00 usec
PLW2            10.00000000 W
PLW12            0.20863999 W
PLW13            0.10495000 W

F2 - Processing parameters
SI                    32768
SF              75.4803210 MHz
WDW                      EM
SSB       0
LB                     1.00 Hz
GB        0
PC                     1.40
```

Data File: C:\LabSolutions\Data\Analiz\uac\TU-22_34.lcd

Elmt	Val.	Min	Max	Elmt	Val.	Min	Max	Elmt	Val.	Min	Max	Elmt	Val.	Min	Max	Use Adduct
H	1	9	40	O	2	1	2	S	2	0	2	Ru	2	0	0	H
C	4	11	35	F	1	0	0	Cl	1	0	1	I	3	0	0	
N	3	0	6	P	3	0	0	Br	1	0	1					

Error Margin (ppm): 5
HC Ratio: unlimited
Max Isotopes: 3
MSn Iso RI (%): 10.00

DBE Range: 17.0 - 20.0
Apply N Rule: yes
Isotope RI (%): 1.00
MSn Logic Mode: AND

Electron Ions: both
Use MSn Info: no
Isotope Res: 10000
Max Results: 500

Event#: 1 MS(E+) Ret. Time : 6.853 -> 6.987 Scan# : 1029 -> 1049

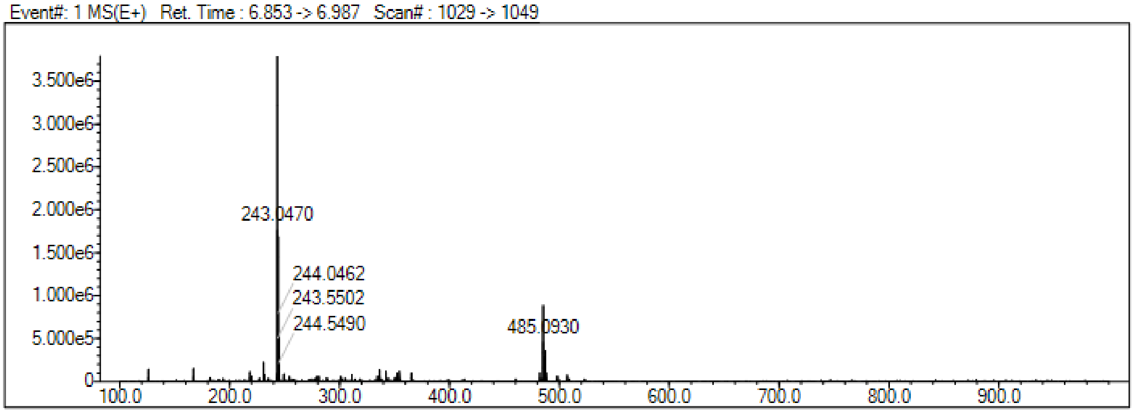

Measured region for 485.0930 m/z

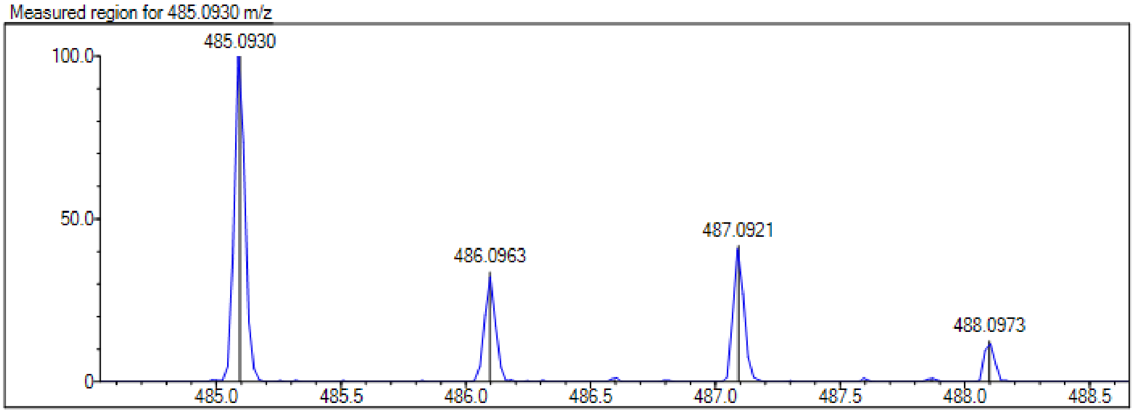

C25 H17 N6 O S Cl [M+H]+ : Predicted region for 485.0946 m/z

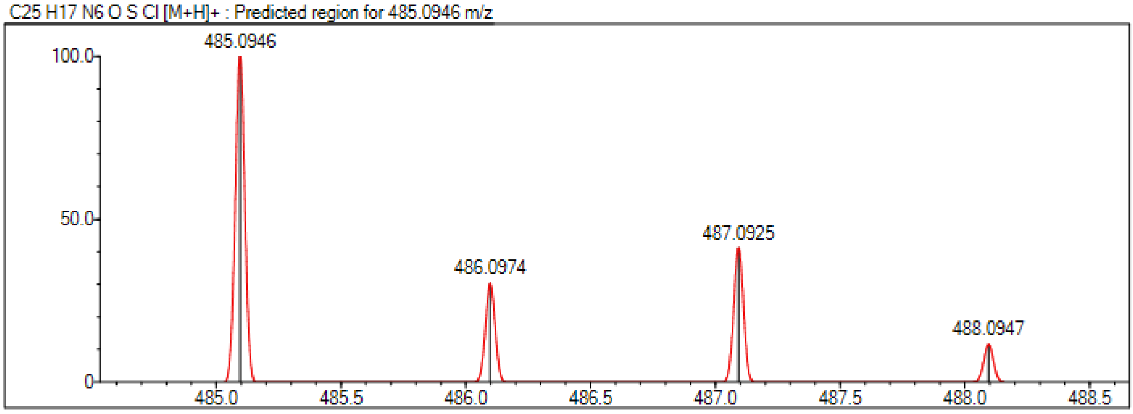

Rank	Score	Formula (M)	Ion	Meas. m/z	Pred. m/z	Df. (mDa)	Df. (ppm)	Iso	DBE
1	72.38	C25 H17 N6 O S Cl	[M+H]+	485.0930	485.0946	-1.6	-3.30	76.80	20.0

2-(4-(4-Methyl-5-(2-(4-bromophenyl)-2-oxo-ethylthio)-4H-1,2,4-triazol-3yl)-phenyl)-5(6)-chloro-1H-benzimidazole (3b)

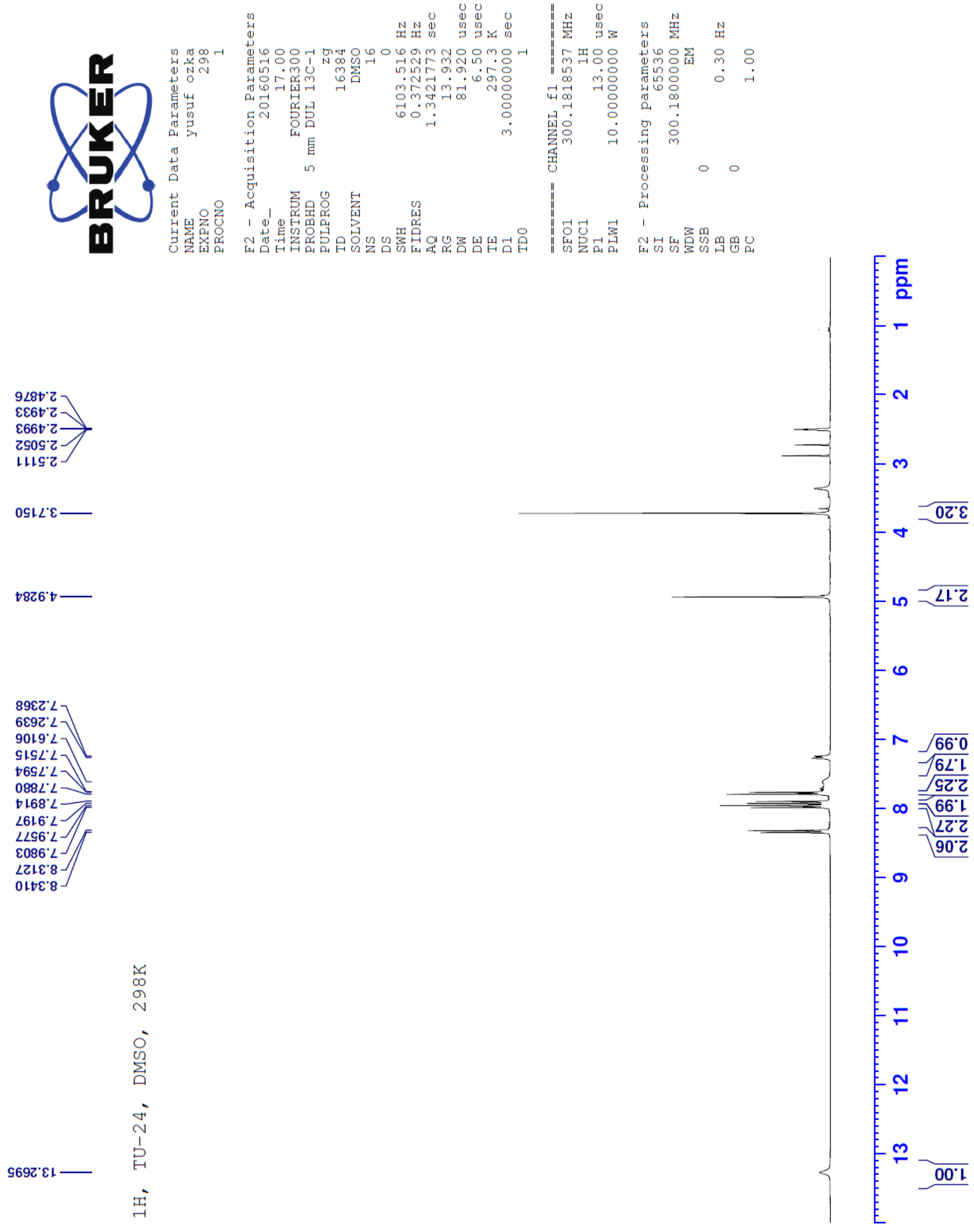

13C, TU-24, DMSO, 298K

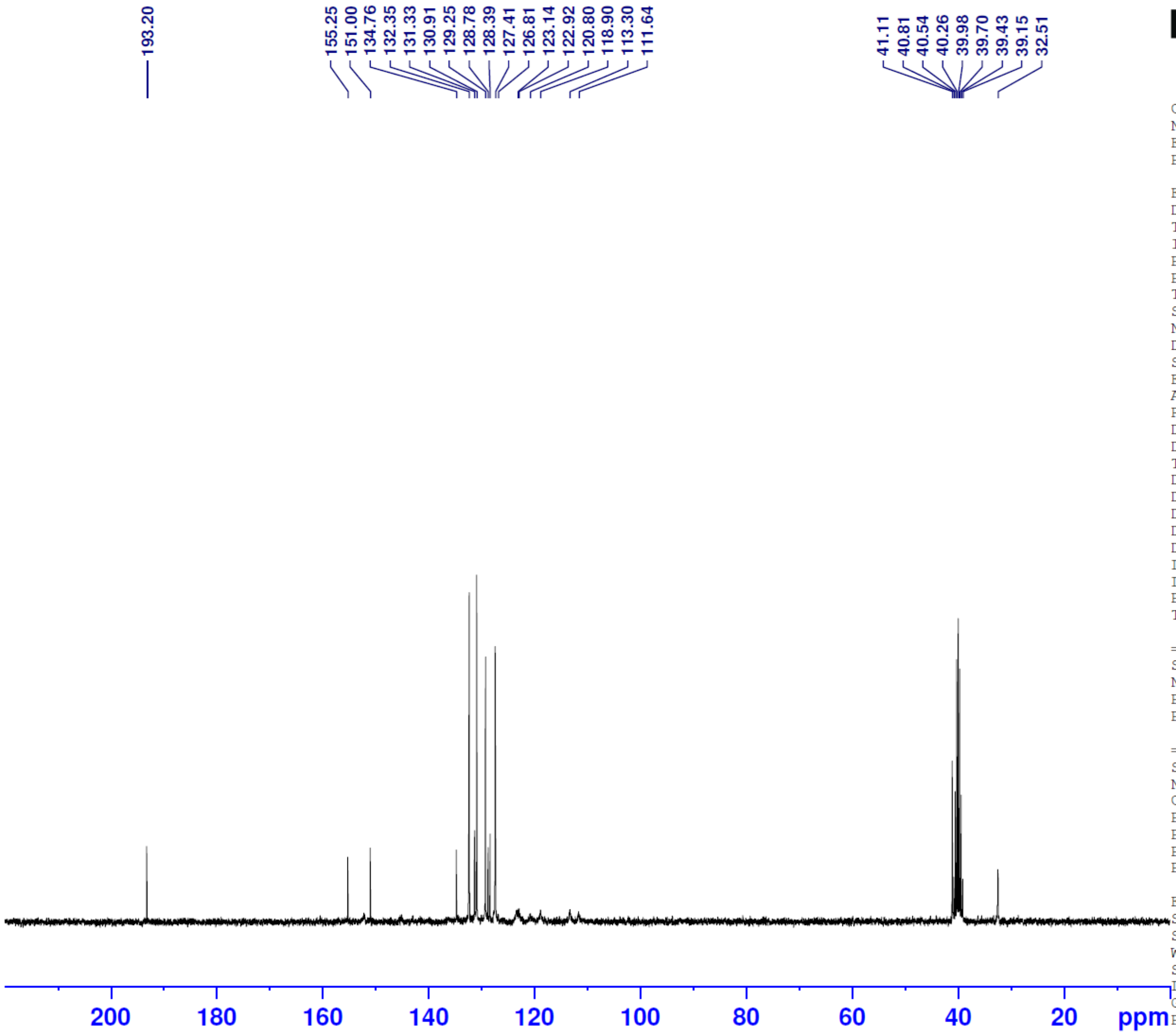


```
Current Data Parameters
NAME            yusuf ozka
EXPNO                  299
PROCNO                   1

F2 - Acquisition Parameters
Date_             20160516
Time                 17.01
INSTRUM        FOURIER300
PROBHD    5 mm DUL 13C-1
PULPROG               zgpg
TD                   32768
SOLVENT               DMSO
NS                    2048
DS                       4
SWH              24414.063 Hz
FIDRES            0.745058 Hz
AQ               0.6710886 sec
RG                 501.187
DW                  20.480 usec
DE                    6.50 usec
TE                   298.0 K
D1              1.00000000 sec
D11             0.03000000 sec
D31             0.00001500 sec
D32             0.89999998 sec
D40             0.00093990 sec
L4                      23
L5                      26
P32                  90.00 usec
TD0                      1

======== CHANNEL f1 ========
SFO1            75.4878687 MHz
NUC1                   13C
P1                   15.00 usec
PLW1           15.00000000 W

======== CHANNEL f2 ========
SFO2           300.1812007 MHz
NUC2                    1H
CPDPRG[2           waltz16
PCPD2                90.00 usec
PLW2           10.00000000 W
PLW12           0.20863999 W
PLW13           0.10495000 W

F2 - Processing parameters
SI                   32768
SF              75.4803210 MHz
WDW                     EM
SSB      0
LB                    1.00 Hz
GB       0
PC                    1.40
```

Data File: C:\LabSolutions\Data\Analiz\uac\TU-24_36.lcd

Elmt	Val.	Min	Max	Elmt	Val.	Min	Max	Elmt	Val.	Min	Max	Elmt	Val.	Min	Max	Use Adduct
H	1	9	40	O	2	1	2	S	2	0	2	Ru	2	0	0	H
C	4	11	35	F	1	0	0	Cl	1	0	1	I	3	0	0	
N	3	0	6	P	3	0	0	Br	1	0	1					

Error Margin (ppm): 10
HC Ratio: unlimited
Max Isotopes: 3
MSn Iso RI (%): 10.00

DBE Range: 17.0 - 20.0
Apply N Rule: yes
Isotope RI (%): 1.00
MSn Logic Mode: AND

Electron Ions: both
Use MSn Info: no
Isotope Res: 10000
Max Results: 500

Event#: 1 MS(E+) Ret. Time : 7.227 -> 7.453 - 8.160 -> 10.844 Scan# : 1085 -> 1119 - 1225 -> 1627

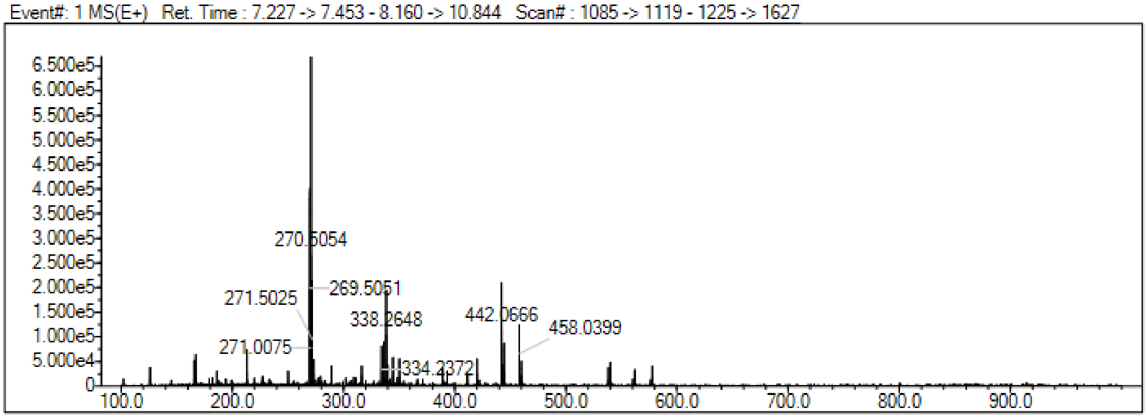

Measured region for 538.0060 m/z

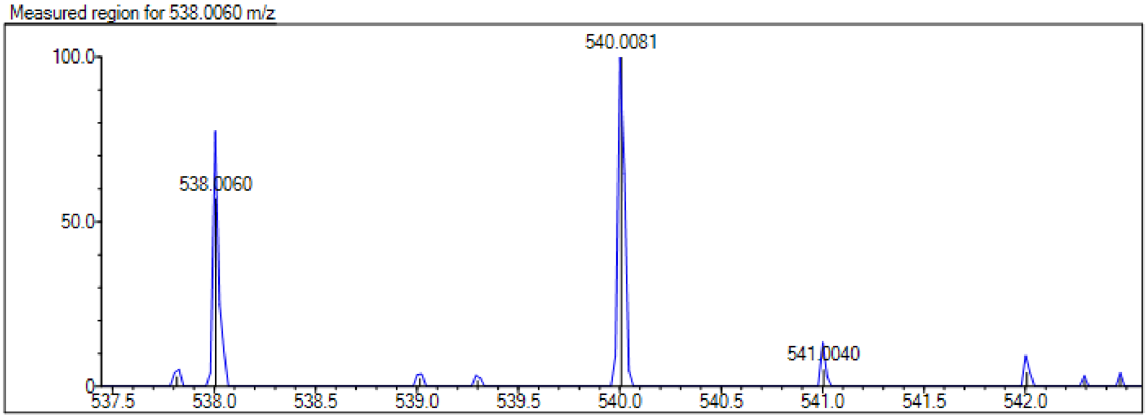

C24 H17 N5 O S Cl Br [M+H]+ : Predicted region for 538.0098 m/z

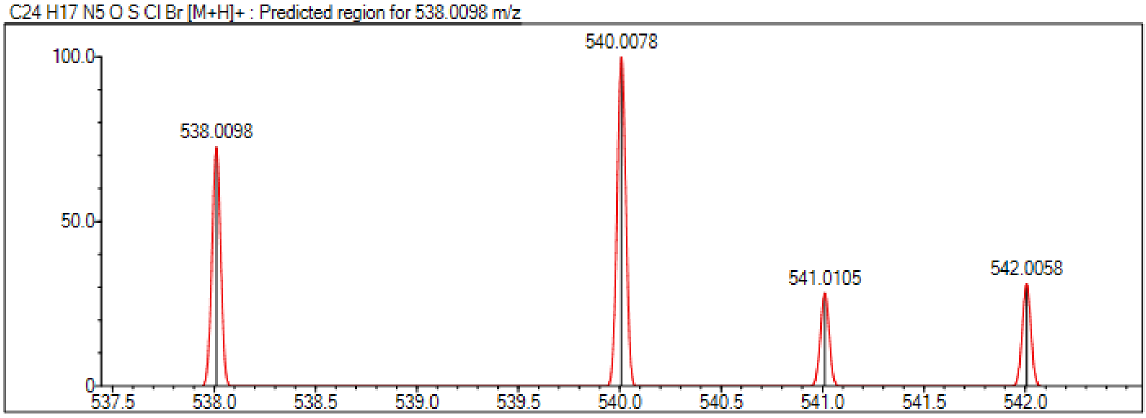

Rank	Score	Formula (M)	Ion	Meas. m/z	Pred. m/z	Df. (mDa)	Df. (ppm)	Iso	DBE
1	22.67	C24 H17 N5 O S Cl Br	[M+H]+	538.0060	538.0098	-3.8	-7.06	32.66	18.0

2-(4-(4-Methyl-5-(2-(4-methylphenyl)-2-oxo-ethylthio)-4H-1,2,4-triazol-3yl)-phenyl)-5(6)-chloro-1H-benzimidazole (3c)

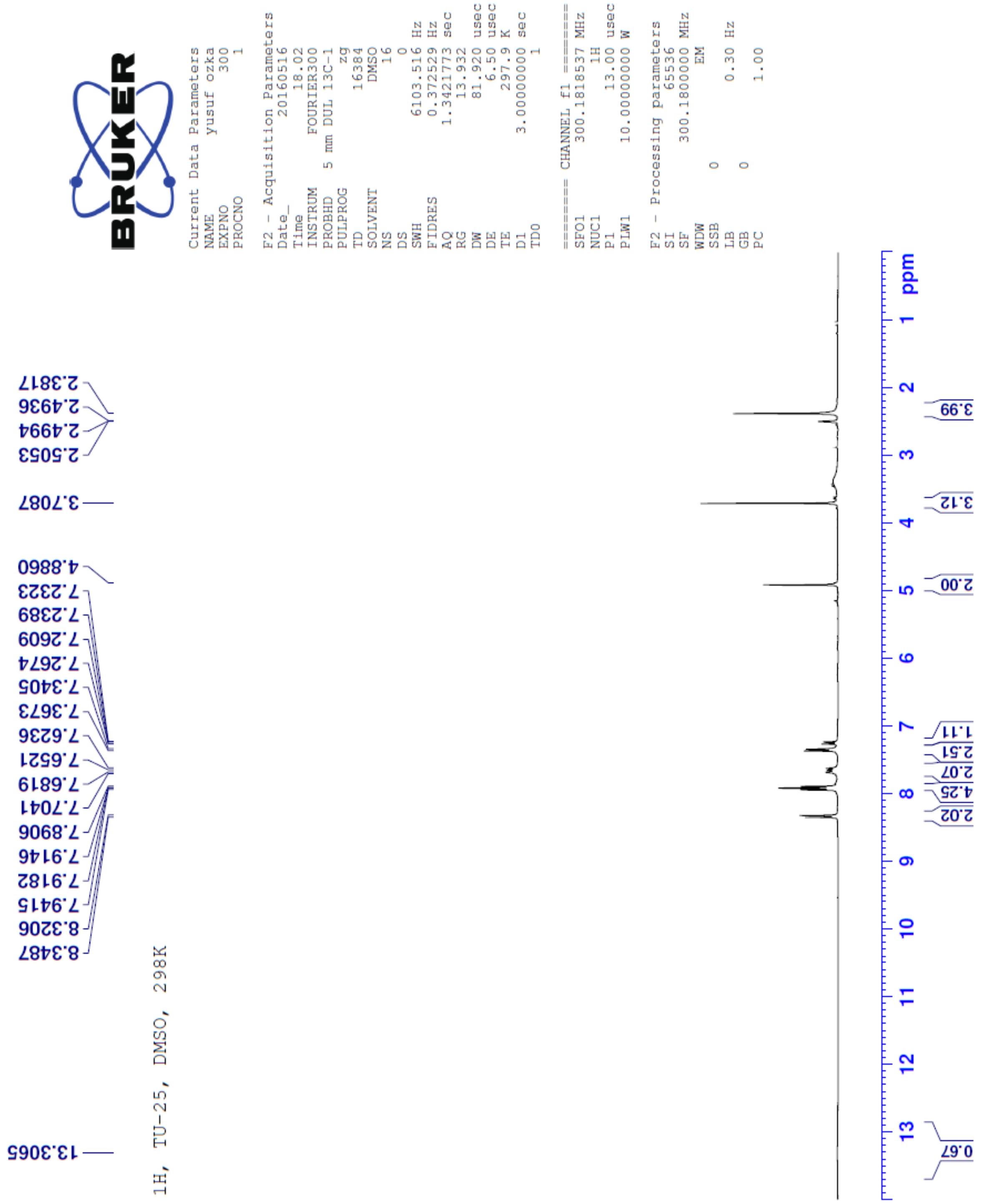

13C, TU-25, DMSO, 298K

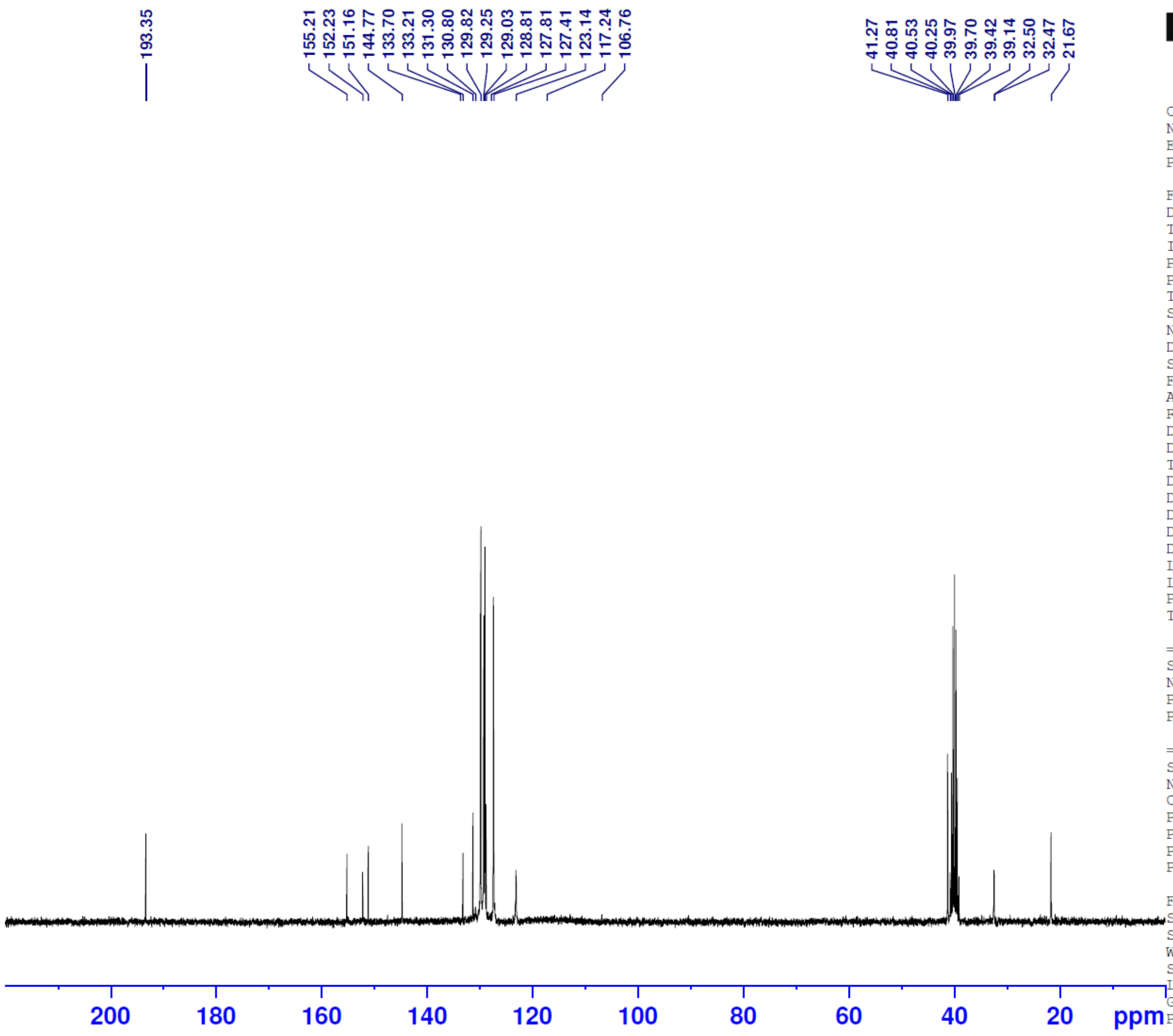


```
Current Data Parameters
NAME             yusuf ozka
EXPNO                   301
PROCNO                    1

F2 - Acquisition Parameters
Date_              20160516
Time                  18.04
INSTRUM          FOURIER300
PROBHD      5 mm DUL 13C-1
PULPROG                zgpg
TD                    32768
SOLVENT                DMSO
NS                     2048
DS                        4
SWH               24414.063 Hz
FIDRES             0.745058 Hz
AQ                0.6710886 sec
RG                  501.187
DW                   20.480 usec
DE                     6.50 usec
TE                    297.9 K
D1               1.00000000 sec
D11              0.03000000 sec
D31              0.00001500 sec
D32              0.89999998 sec
D40              0.00093990 sec
L4                       23
L5                       26
P32                   90.00 usec
TD0                       1

======== CHANNEL f1 ========
SFO1            75.4878687 MHz
NUC1                   13C
P1                   15.00 usec
PLW1           15.00000000 W

======== CHANNEL f2 ========
SFO2           300.1812007 MHz
NUC2                    1H
CPDPRG[2           waltz16
PCPD2                90.00 usec
PLW2           10.00000000 W
PLW12           0.20863999 W
PLW13           0.10495000 W

F2 - Processing parameters
SI                   32768
SF              75.4803210 MHz
WDW                     EM
SSB      0
LB                    1.00 Hz
GB       0
PC                    1.40
```

Data File: C:\LabSolutions\Data\Analiz\uac\TU-25_37.lcd

Elmt	Val.	Min	Max	Elmt	Val.	Min	Max	Elmt	Val.	Min	Max	Elmt	Val.	Min	Max	Use Adduct
H	1	9	40	O	2	1	2	S	2	0	2	Ru	2	0	0	H
C	4	11	35	F	1	0	0	Cl	1	0	1	I	3	0	0	
N	3	0	6	P	3	0	0	Br	1	0	1					

Error Margin (ppm):	10	DBE Range:	17.0 - 20.0	Electron Ions:	both
HC Ratio:	unlimited	Apply N Rule:	yes	Use MSn Info:	no
Max Isotopes:	3	Isotope RI (%):	1.00	Isotope Res:	10000
MSn Iso RI (%):	10.00	MSn Logic Mode:	AND	Max Results:	500

Event#: 1 MS(E+) Ret. Time : 7.040 -> 7.400 - 7.933 -> 10.272 Scan# : 1057 -> 1111 - 1191 -> 1541

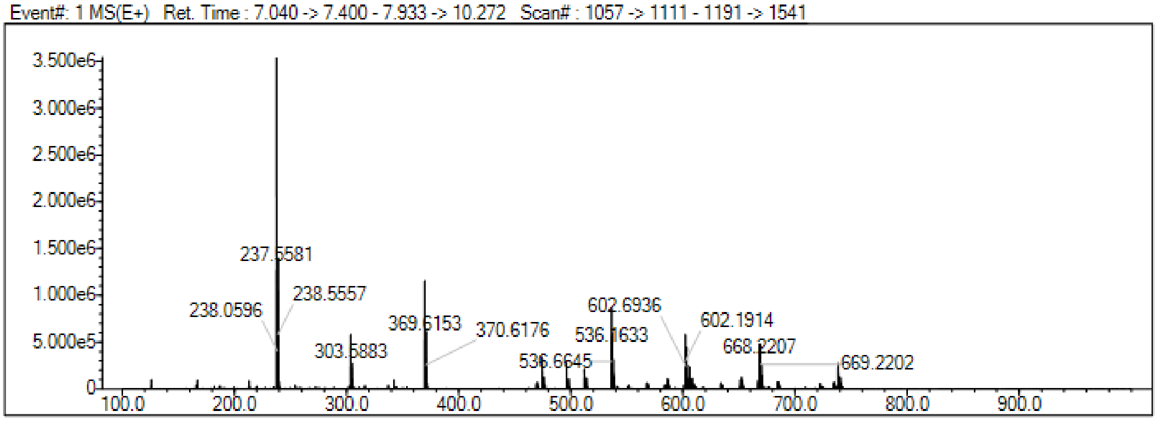

Measured region for 474.1148 m/z

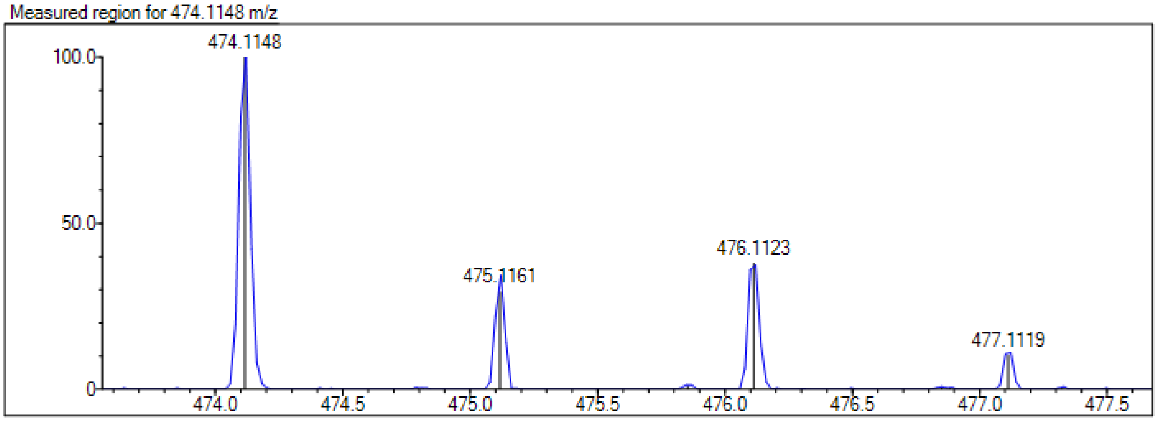

C25 H20 N5 O S Cl [M+H]+ : Predicted region for 474.1150 m/z

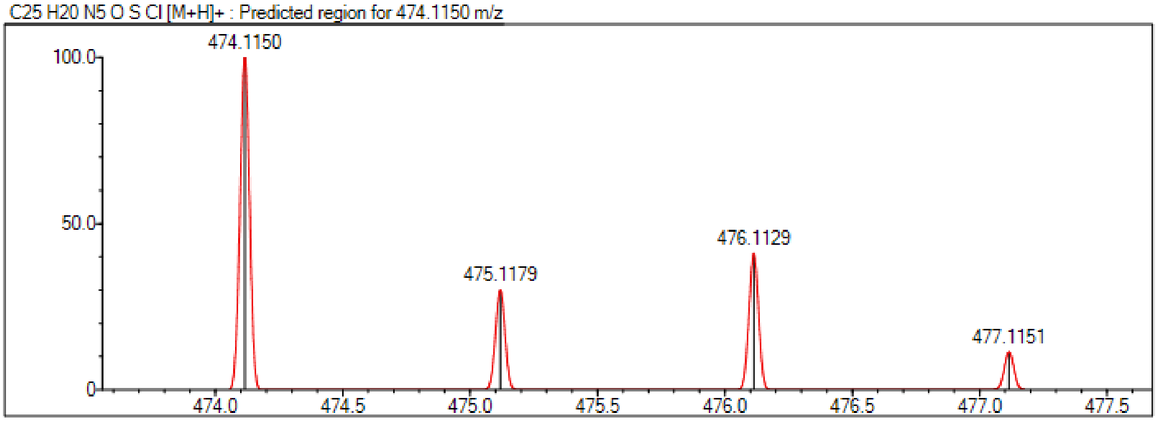

Rank	Score	Formula (M)	Ion	Meas. m/z	Pred. m/z	Df. (mDa)	Df. (ppm)	Iso	DBE
1	88.86	C25 H20 N5 O S Cl	[M+H]+	474.1148	474.1150	-0.2	-0.42	88.86	18.0

2-(4-(4-Methyl-5-(2-(3,4-dihydroxyphenyl)-2-oxo-ethylthio)-4H-1,2,4-triazol-3yl)-phenyl)-5(6)-chloro-1H- benzimidazole (3d)

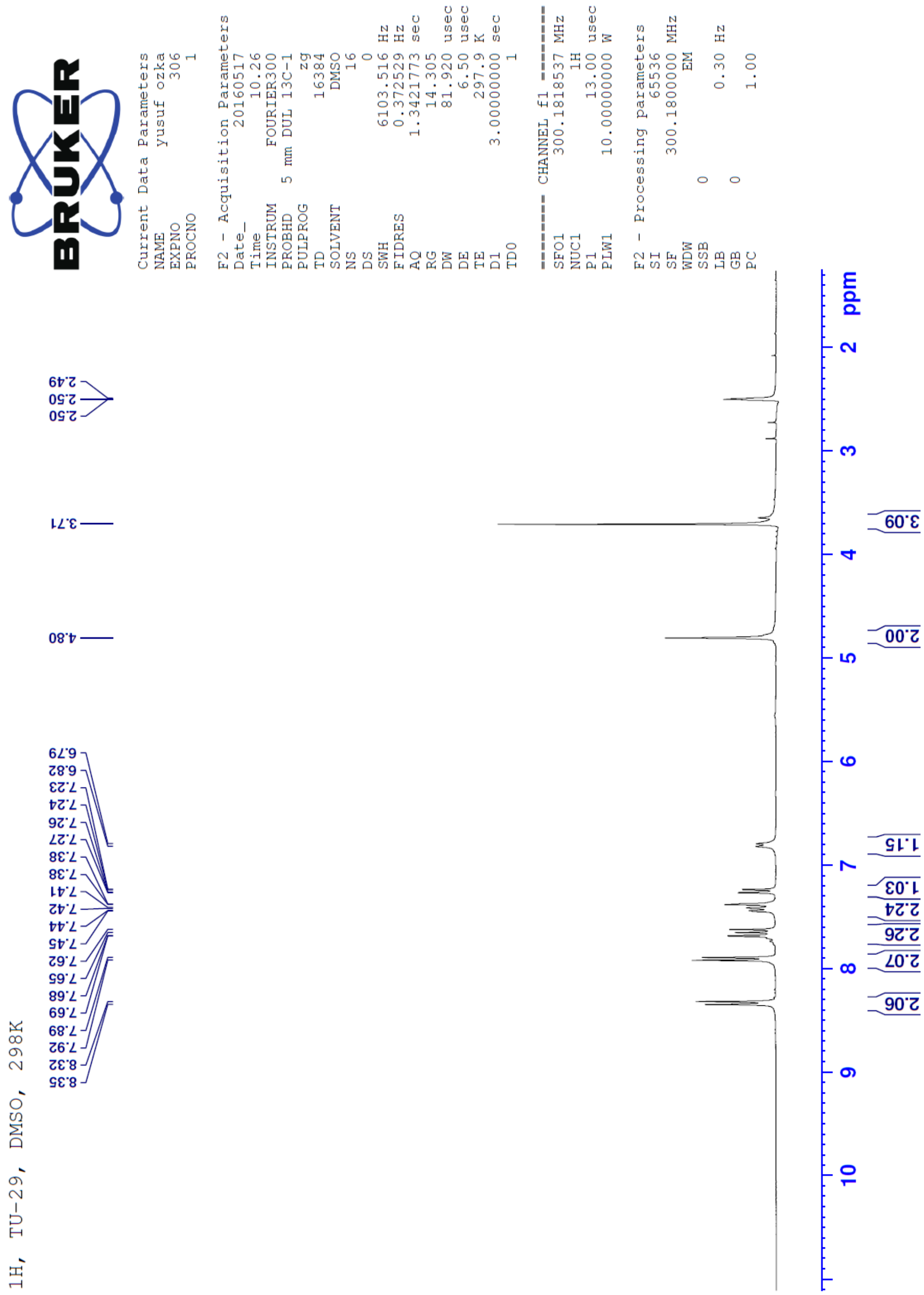

13C, TU-29, DMSO, 298K

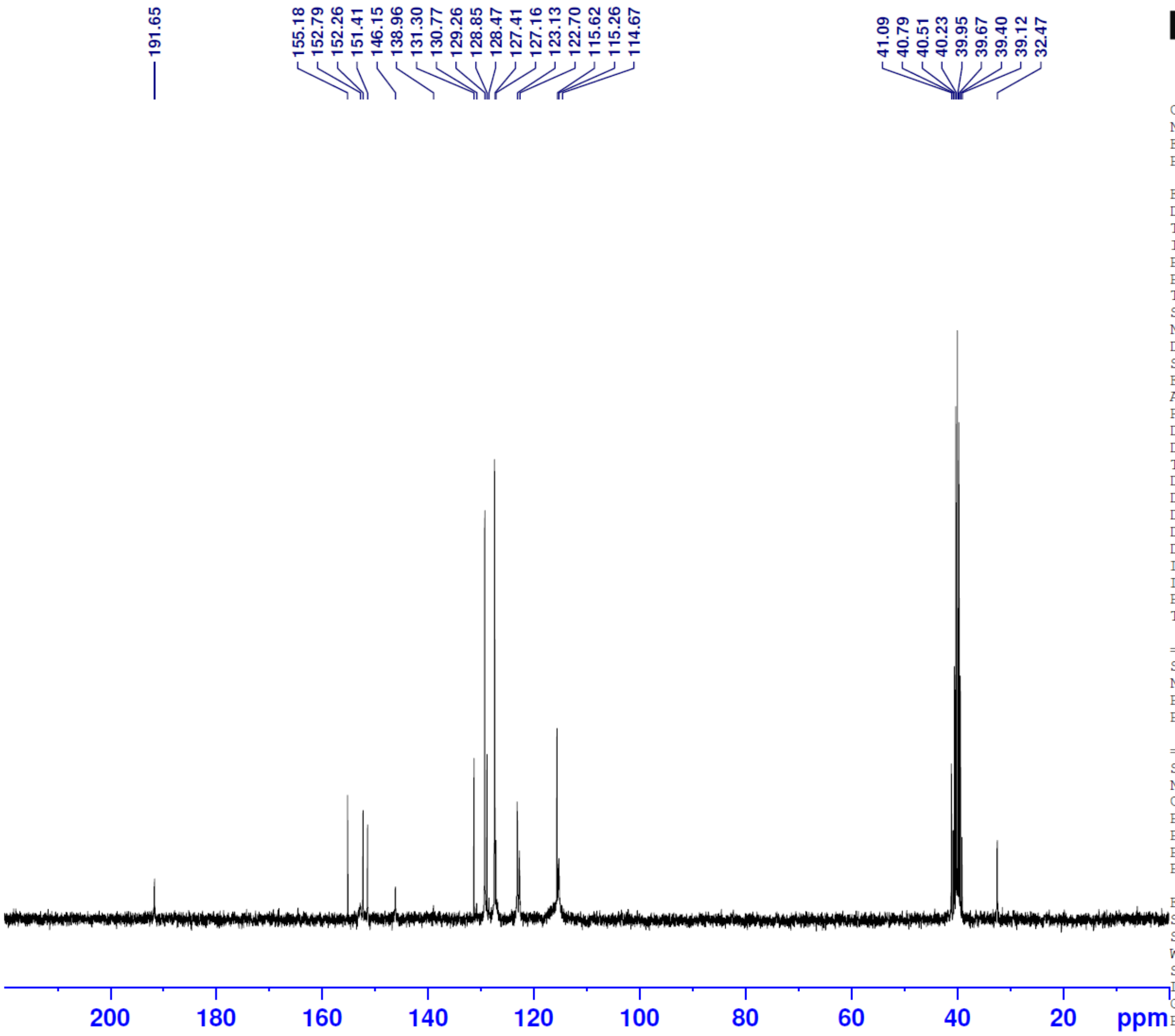


```
Current Data Parameters
NAME            yusuf ozka
EXPNO                  307
PROCNO                   1

F2 - Acquisition Parameters
Date_             20160517
Time                 10.28
INSTRUM         FOURIER300
PROBHD     5 mm DUL 13C-1
PULPROG               zgpg
TD                   32768
SOLVENT               DMSO
NS                    2048
DS                       4
SWH              24414.063 Hz
FIDRES            0.745058 Hz
AQ               0.6710886 sec
RG                 501.187
DW                  20.480 usec
DE                    6.50 usec
TE                   298.0 K
D1              1.00000000 sec
D11             0.03000000 sec
D31             0.00001500 sec
D32             0.89999998 sec
D40             0.00093990 sec
L4                      23
L5                      26
P32                  90.00 usec
TD0                      1

======== CHANNEL f1 ========
SFO1            75.4878687 MHz
NUC1                   13C
P1                   15.00 usec
PLW1           15.00000000 W

======== CHANNEL f2 ========
SFO2           300.1812007 MHz
NUC2                    1H
CPDPRG[2           waltz16
PCPD2                90.00 usec
PLW2           10.00000000 W
PLW12           0.20863999 W
PLW13           0.10495000 W

F2 - Processing parameters
SI                   32768
SF              75.4803210 MHz
WDW                     EM
SSB      0
LB                    1.00 Hz
GB       0
PC                    1.40
```

Data File: C:\LabSolutions\Data\Analiz\uac\TU-29_42.lcd

Elmt	Val.	Min	Max	Elmt	Val.	Min	Max	Elmt	Val.	Min	Max	Elmt	Val.	Min	Max	Use Adduct
H	1	9	40	O	2	3	3	S	2	0	2	Ru	2	0	0	H
C	4	11	35	F	1	0	0	Cl	1	0	1	I	3	0	0	
N	3	0	6	P	3	0	0	Br	1	0	1					

Error Margin (ppm): 10
HC Ratio: unlimited
Max Isotopes: 3
MSn Iso RI (%): 10.00

DBE Range: 17.0 - 20.0
Apply N Rule: yes
Isotope RI (%): 1.00
MSn Logic Mode: AND

Electron Ions: both
Use MSn Info: no
Isotope Res: 10000
Max Results: 500

Event#: 1 MS(E+) Ret. Time : 6.160 -> 6.787 - 7.627 -> 8.973 Scan# : 925 -> 1019 - 1145 -> 1347

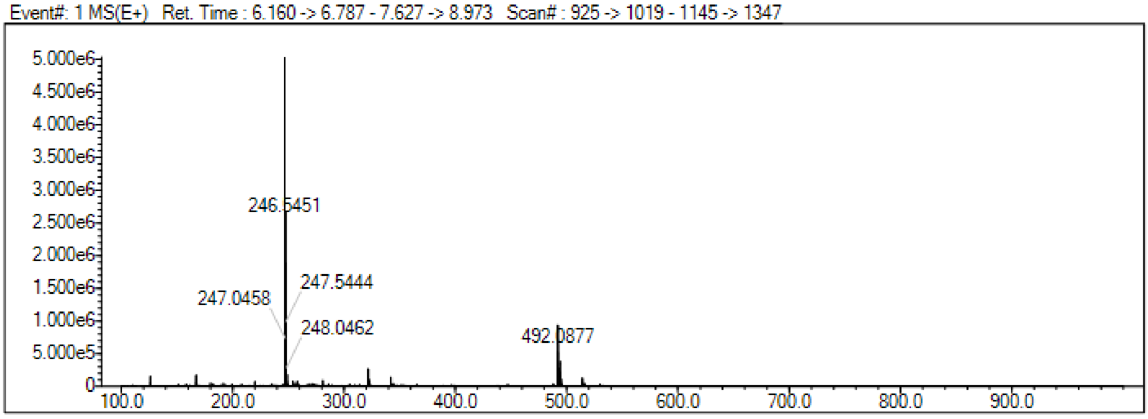

Measured region for 492.0877 m/z

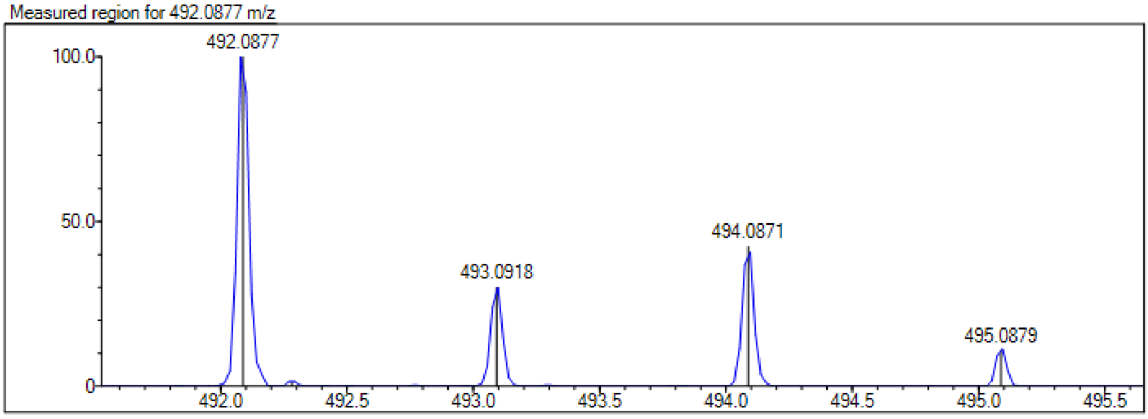

C24 H18 N5 O3 S Cl [M+H]+ : Predicted region for 492.0892 m/z

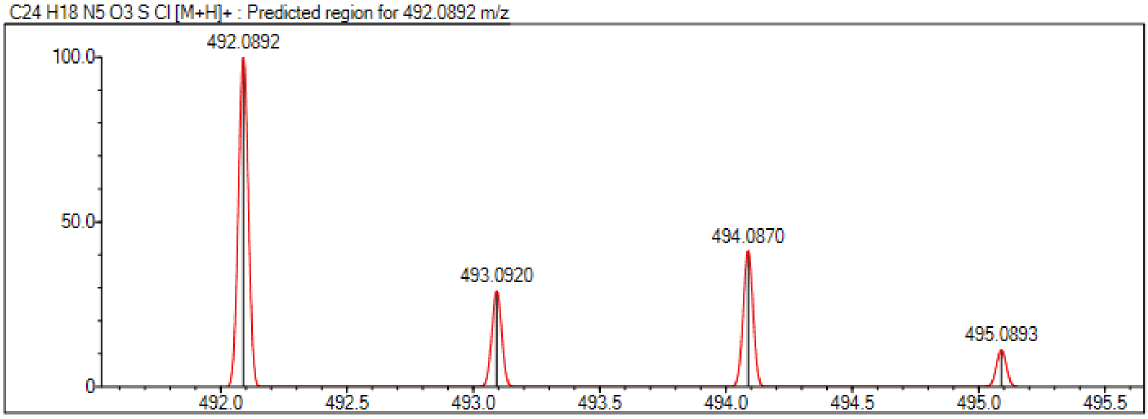

Rank	Score	Formula (M)	Ion	Meas. m/z	Pred. m/z	Df. (mDa)	Df. (ppm)	Iso	DBE
1	88.95	C24 H18 N5 O3 S Cl	[M+H]+	492.0877	492.0892	-1.5	-3.05	93.75	18.0

2-(4-(4-Ethyl-5-(2-(4-cyanophenyl)-2-oxo-ethylthio)-4H-1,2,4-triazol-3yl)-phenyl)-5(6)-chloro-1H-benzoimidazole (3e)

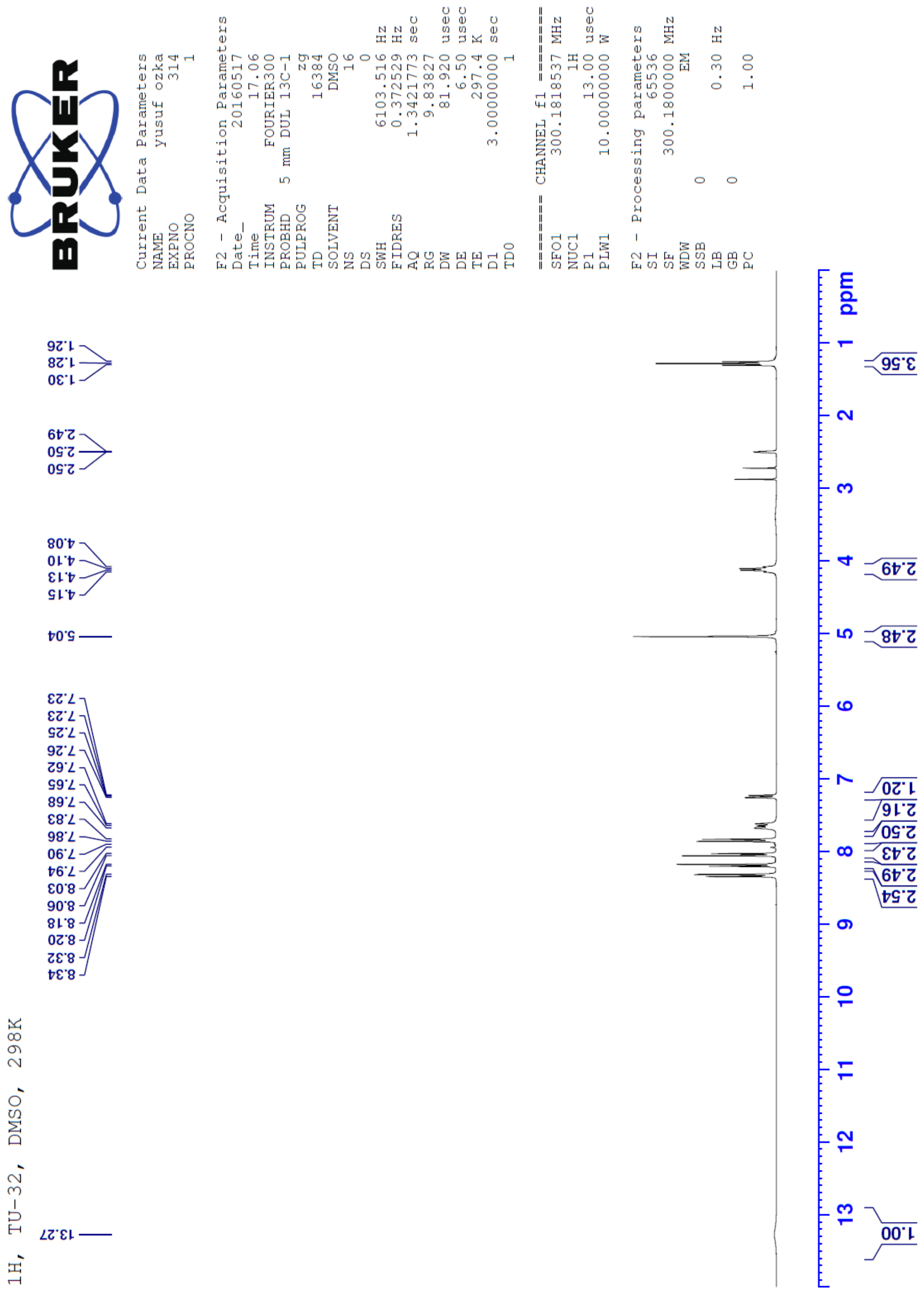

13C, TU-32, DMSO, 298K

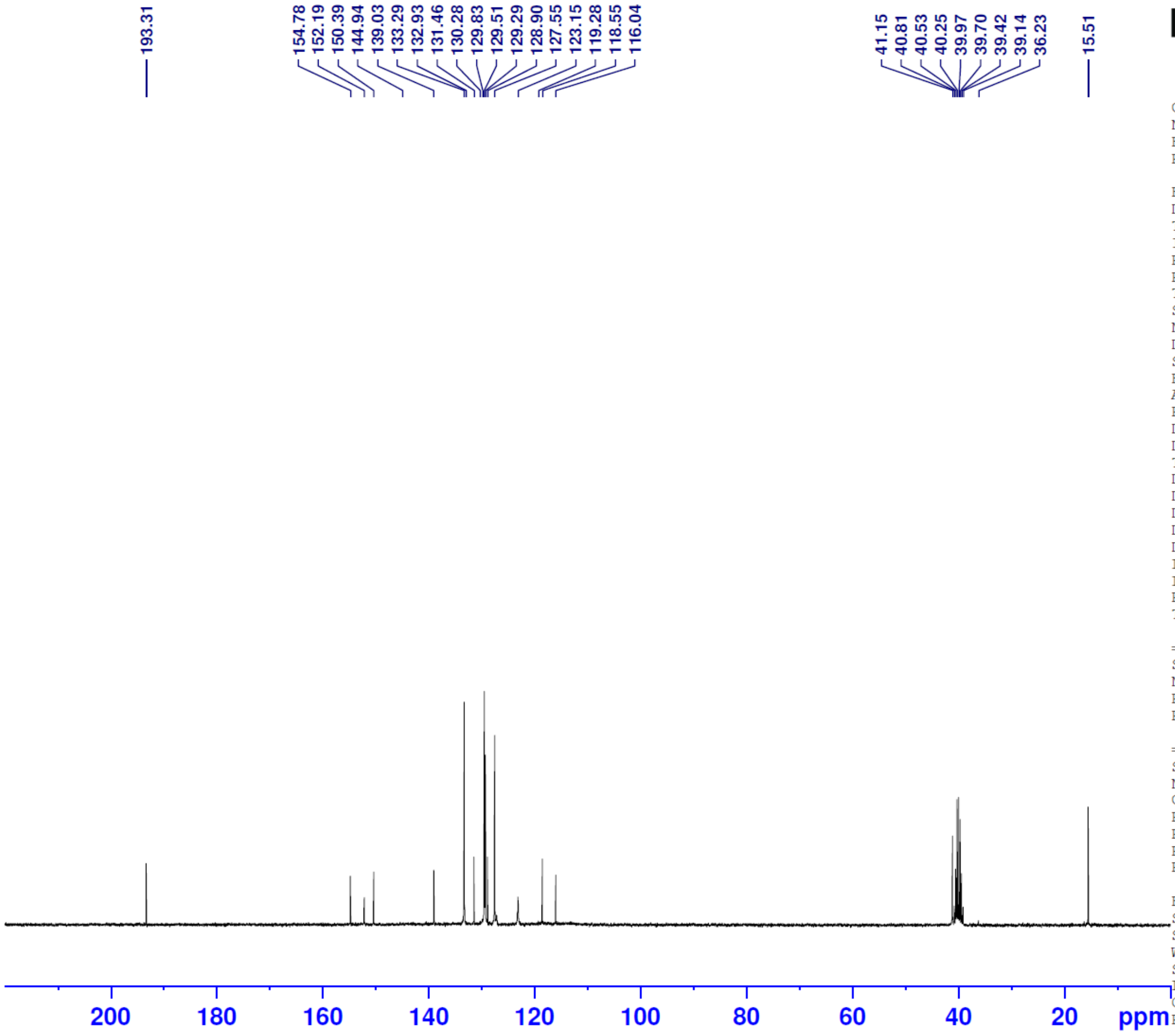


```
Current Data Parameters
NAME          yusuf ozka
EXPNO                315
PROCNO                 1

F2 - Acquisition Parameters
Date_           20160517
Time               17.08
INSTRUM      FOURIER300
PROBHD   5 mm DUL 13C-1
PULPROG             zgpg
TD                 32768
SOLVENT             DMSO
NS                  2048
DS                     4
SWH            24414.063 Hz
FIDRES          0.745058 Hz
AQ             0.6710886 sec
RG               501.187
DW                20.480 usec
DE                  6.50 usec
TE                 297.9 K
D1            1.00000000 sec
D11           0.03000000 sec
D31           0.00001500 sec
D32           0.89999998 sec
D40           0.00093990 sec
L4                    23
L5                    26
P32                90.00 usec
TD0                    1

======== CHANNEL f1 ========
SFO1          75.4878687 MHz
NUC1                 13C
P1                 15.00 usec
PLW1         15.00000000 W

======== CHANNEL f2 ========
SFO2         300.1812007 MHz
NUC2                  1H
CPDPRG[2         waltz16
PCPD2              90.00 usec
PLW2         10.00000000 W
PLW12         0.20863999 W
PLW13         0.10495000 W

F2 - Processing parameters
SI                 32768
SF            75.4803210 MHz
WDW                   EM
SSB      0
LB                  1.00 Hz
GB       0
PC                  1.40
```

Data File: C:\LabSolutions\Data\Analiz\uac\TU-32_46.lcd

Elmt	Val.	Min	Max	Elmt	Val.	Min	Max	Elmt	Val.	Min	Max	Elmt	Val.	Min	Max	Use Adduct
H	1	9	40	O	2	1	1	S	2	0	2	Ru	2	0	0	H
C	4	11	26	F	1	0	0	Cl	1	0	1	I	3	0	0	
N	3	0	6	P	3	0	0	Br	1	0	1					

Error Margin (ppm):	5	DBE Range:	17.0 - 20.0	Electron Ions:	both
HC Ratio:	unlimited	Apply N Rule:	yes	Use MSn Info:	no
Max Isotopes:	3	Isotope RI (%):	1.00	Isotope Res:	10000
MSn Iso RI (%):	10.00	MSn Logic Mode:	AND	Max Results:	500

Event#: 1 MS(E+) Ret. Time : 7.027 -> 7.347 - 8.240 -> 9.284 Scan# : 1055 -> 1103 - 1237 -> 1393

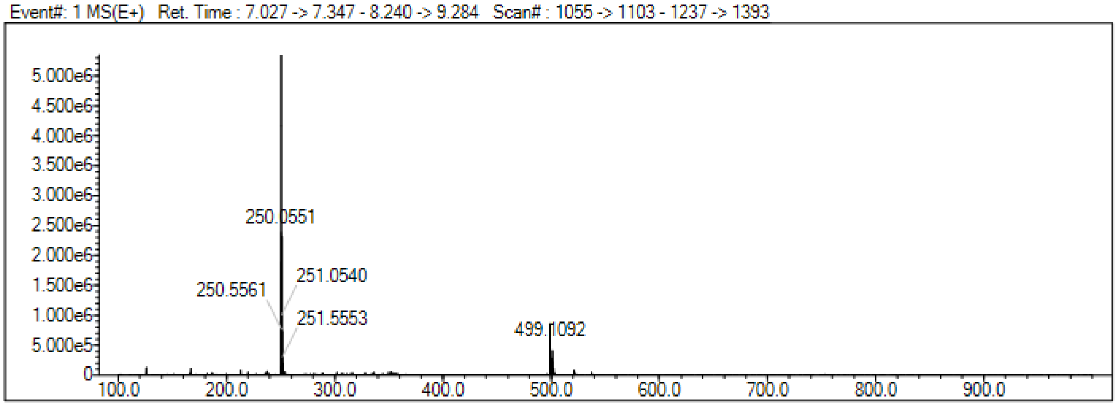

Measured region for 499.1092 m/z

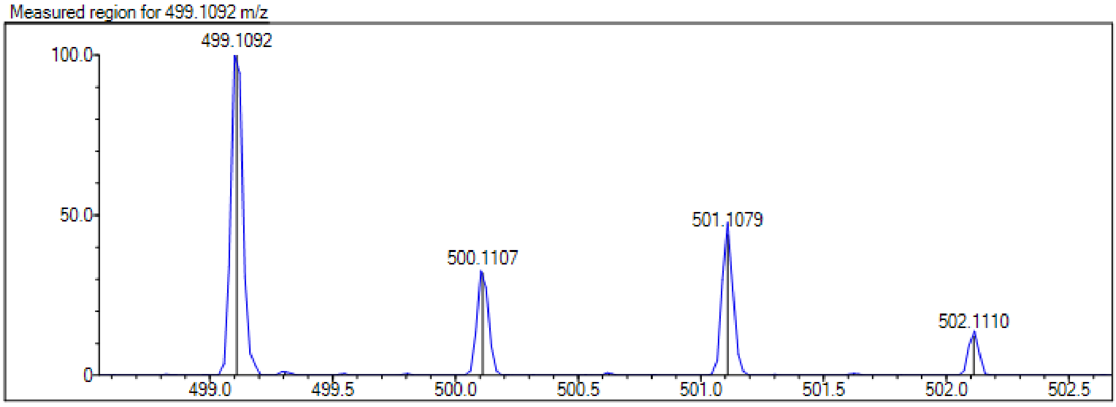

C26 H19 N6 O S Cl [M+H]+ : Predicted region for 499.1102 m/z

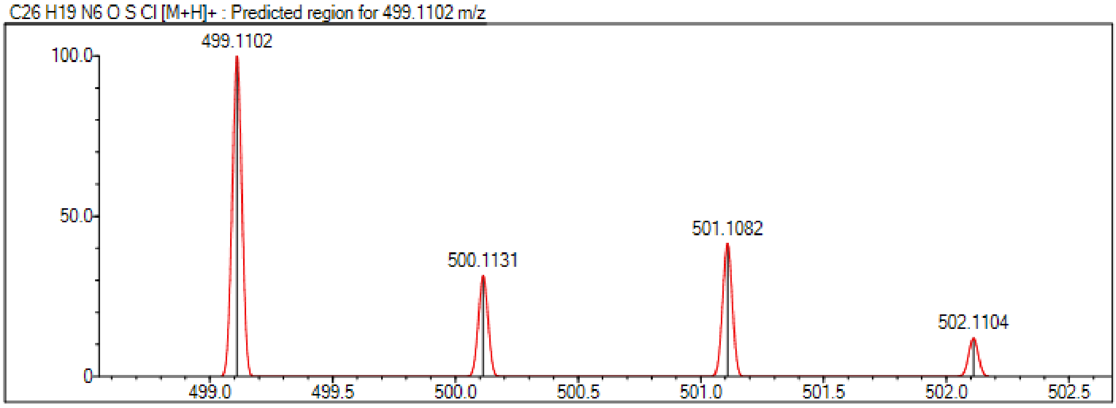

Rank	Score	Formula (M)	Ion	Meas. m/z	Pred. m/z	Df. (mDa)	Df. (ppm)	Iso	DBE
1	86.52	C26 H19 N6 O S Cl	[M+H]+	499.1092	499.1102	-1.0	-2.00	88.74	20.0

2-(4-(4-Ethyl-5-(2-(4-bromophenyl)-2-oxo-ethylthio)-4H-1,2,4-triazol-3yl)-phenyl)-5(6)-chloro-1H-benzimidazole (3f)

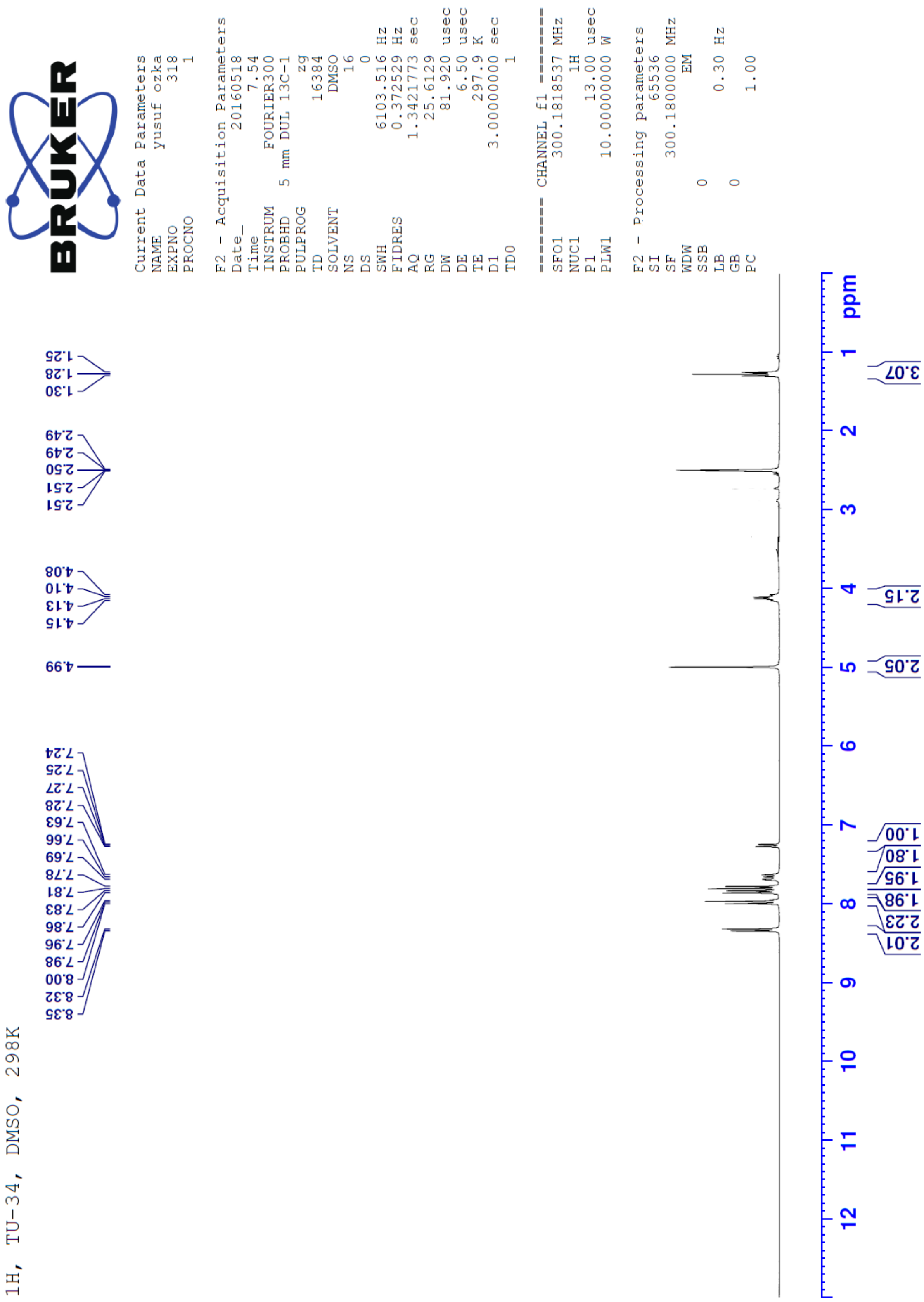

13C, TU-34, DMSO, 298K

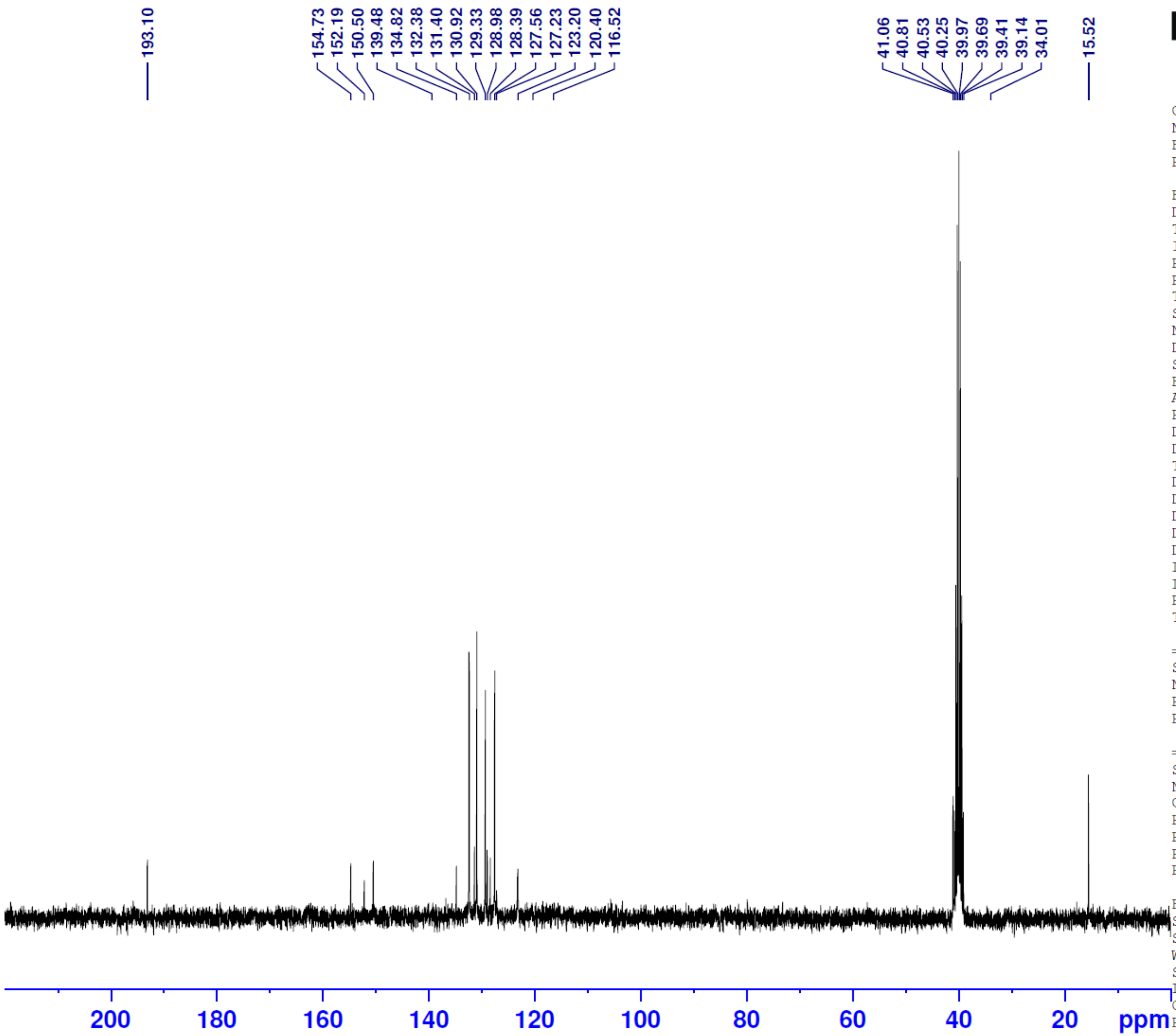

BRUKER

```
Current Data Parameters
NAME          yusuf ozka
EXPNO                319
PROCNO                 1

F2 - Acquisition Parameters
Date_           20160518
Time                7.56
INSTRUM       FOURIER300
PROBHD   5 mm DUL 13C-1
PULPROG             zgpg
TD                 32768
SOLVENT             DMSO
NS                  2048
DS                     4
SWH            24414.063 Hz
FIDRES          0.745058 Hz
AQ             0.6710886 sec
RG               501.187
DW                20.480 usec
DE                  6.50 usec
TE                 298.0 K
D1            1.00000000 sec
D11           0.03000000 sec
D31           0.00001500 sec
D32           0.89999998 sec
D40           0.00093990 sec
L4                    23
L5                    26
P32                90.00 usec
TD0                    1

======== CHANNEL f1 ========
SFO1          75.4878687 MHz
NUC1                 13C
P1                 15.00 usec
PLW1         15.00000000 W

======== CHANNEL f2 ========
SFO2         300.1812007 MHz
NUC2                  1H
CPDPRG[2         waltz16
PCPD2              90.00 usec
PLW2         10.00000000 W
PLW12         0.20863999 W
PLW13         0.10495000 W

F2 - Processing parameters
SI                 32768
SF            75.4803210 MHz
WDW                   EM
SSB      0
LB                  1.00 Hz
GB       0
PC                  1.40
```

Data File: C:\LabSolutions\Data\Analiz\uac\TU-34_48.lcd

Elmt	Val.	Min	Max	Elmt	Val.	Min	Max	Elmt	Val.	Min	Max	Elmt	Val.	Min	Max	Use Adduct
H	1	9	40	O	2	1	1	S	2	0	2	Ru	2	0	0	H
C	4	11	26	F	1	0	0	Cl	1	0	1	I	3	0	0	
N	3	0	6	P	3	0	0	Br	1	0	1					

Error Margin (ppm):	5	DBE Range:	17.0 - 20.0	Electron Ions:	both
HC Ratio:	unlimited	Apply N Rule:	yes	Use MSn Info:	no
Max Isotopes:	3	Isotope RI (%):	1.00	Isotope Res:	10000
MSn Iso RI (%):	10.00	MSn Logic Mode:	AND	Max Results:	500

Event#: 1 MS(E+) Ret. Time : 7.480 -> 7.680 - 8.040 -> 9.784 Scan# : 1123 -> 1153 - 1207 -> 1469

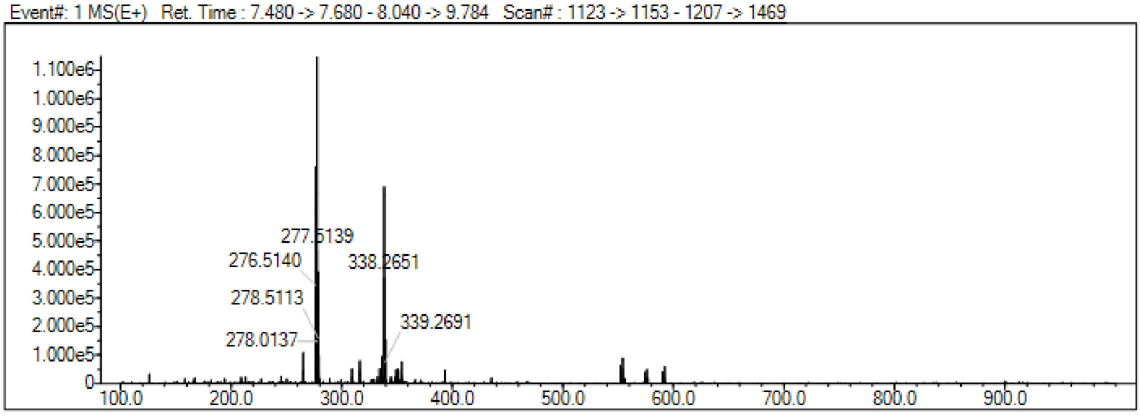

Measured region for 552.0249 m/z

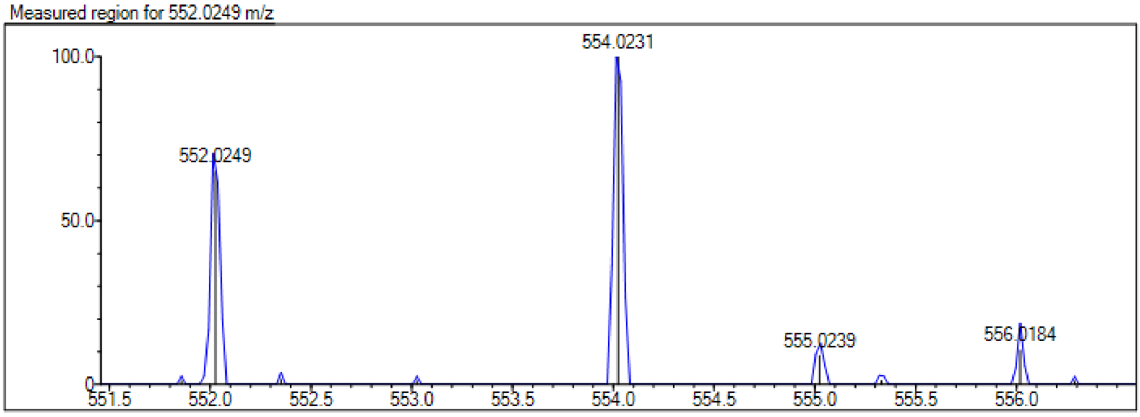

C25 H19 N5 O S Cl Br [M+H]+ : Predicted region for 552.0255 m/z

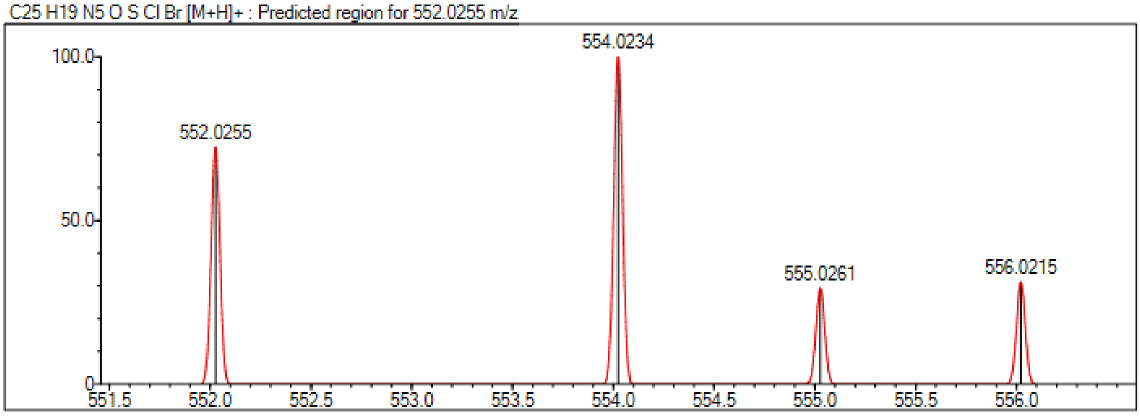

Rank	Score	Formula (M)	Ion	Meas. m/z	Pred. m/z	Df. (mDa)	Df. (ppm)	Iso	DBE
1	54.11	C25 H19 N5 O S Cl Br	[M+H]+	552.0249	552.0255	-0.6	-1.09	54.23	18.0

2-(4-(4-Ethyl-5-(2-(4-methylphenyl)-2-oxo-ethylthio)-4H-1,2,4-triazol-3yl)-phenyl)-5(6)-chloro-1H-benzimidazole (3g)

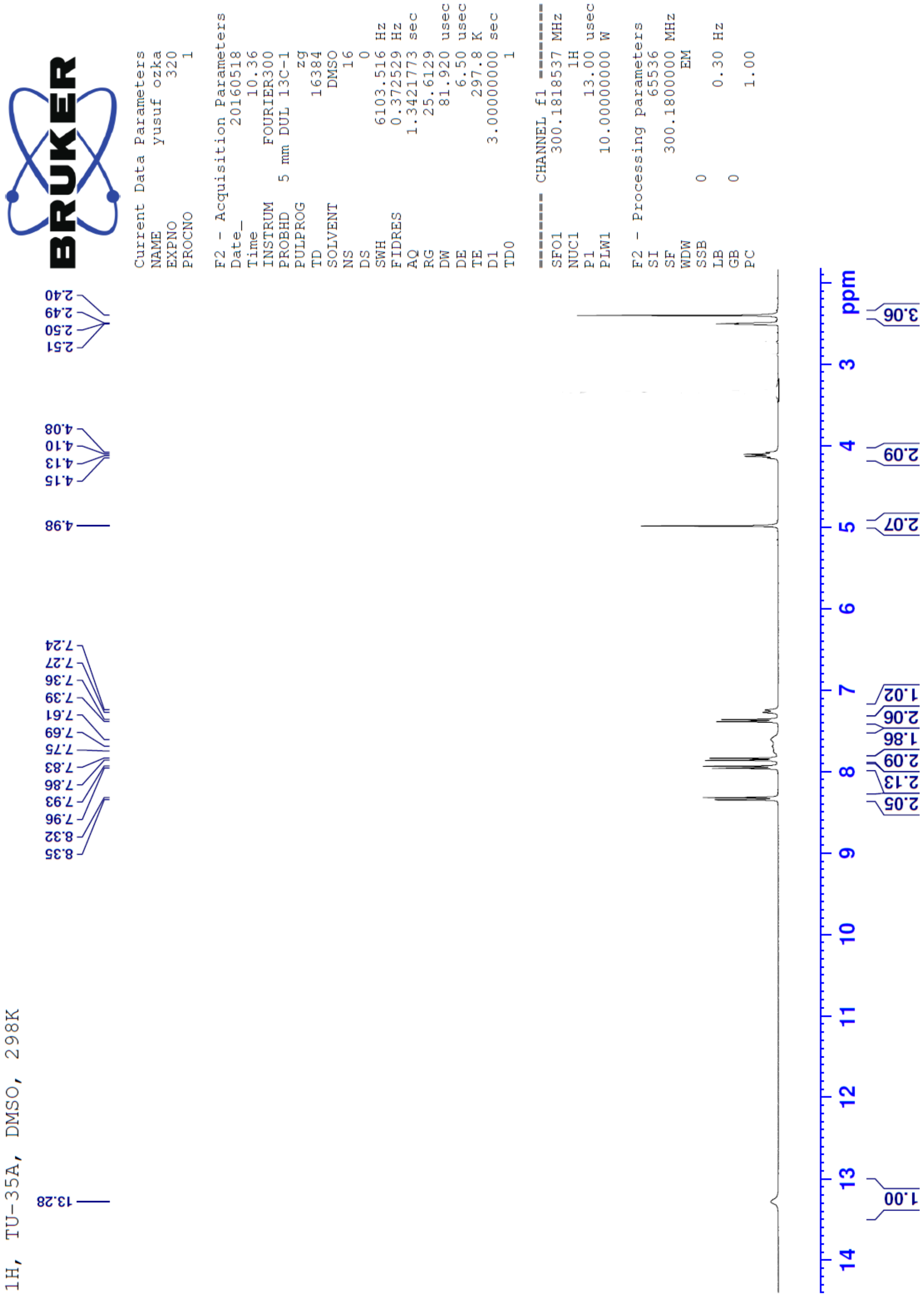

13C, TU-35A, DMSO, 298K

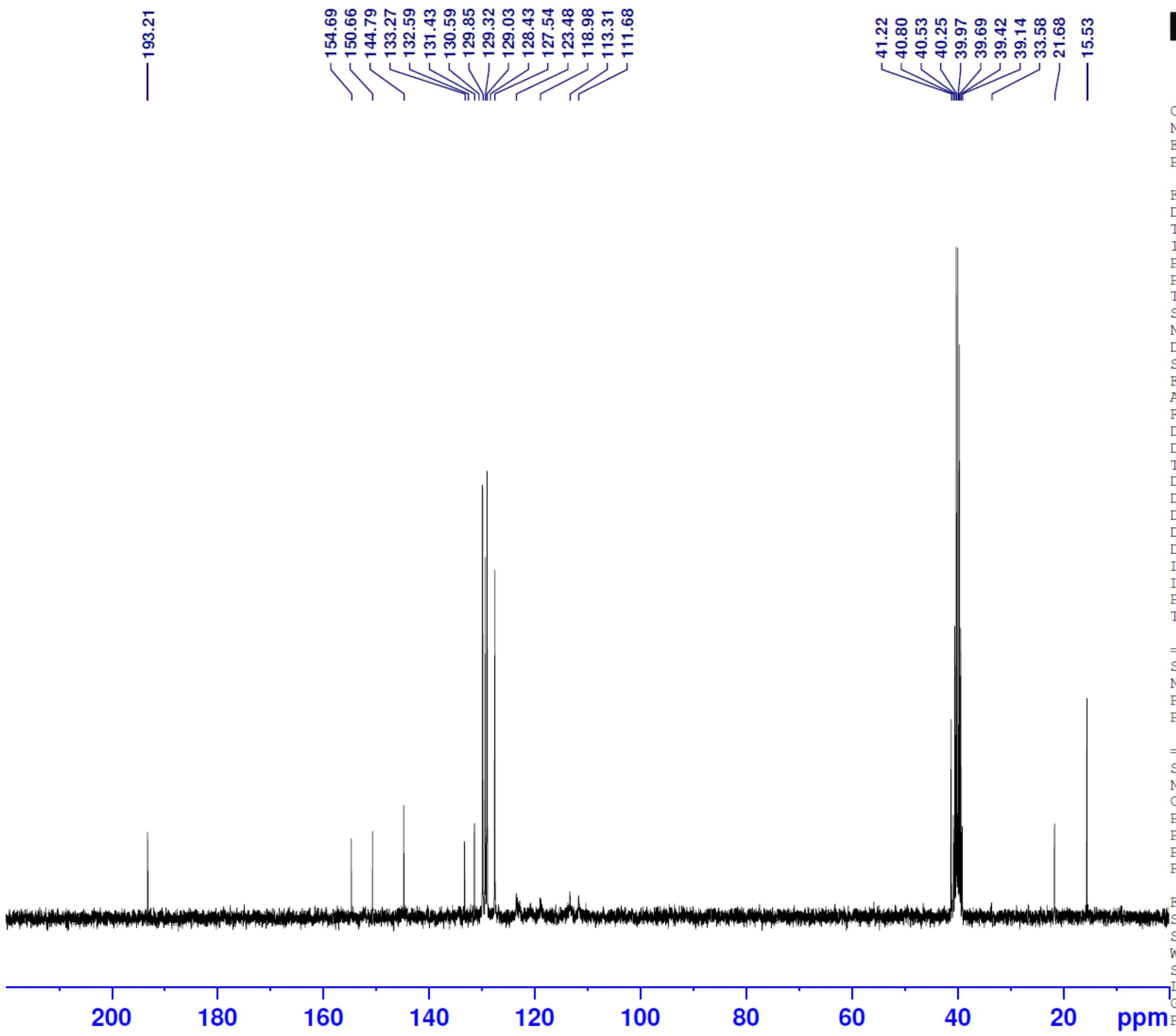

BRUKER

Current Data Parameters

NAME	yusuf ozka
EXPNO	321
PROCNO	1

F2 - Acquisition Parameters

Date_	20160518
Time	10.38
INSTRUM	FOURIER300
PROBHD	5 mm DUL 13C-1
PULPROG	zgpg
TD	32768
SOLVENT	DMSO
NS	2048
DS	4
SWH	24414.063 Hz
FIDRES	0.745058 Hz
AQ	0.6710886 sec
RG	501.187
DW	20.480 usec
DE	6.50 usec
TE	297.9 K
D1	1.00000000 sec
D11	0.03000000 sec
D31	0.00001500 sec
D32	0.89999998 sec
D40	0.00093990 sec
L4	23
L5	26
P32	90.00 usec
TD0	1

======== CHANNEL f1 ========

SFO1	75.4878687 MHz
NUC1	13C
P1	15.00 usec
PLW1	15.00000000 W

======== CHANNEL f2 ========

SFO2	300.1812007 MHz
NUC2	1H
CPDPRG[2	waltz16
PCPD2	90.00 usec
PLW2	10.00000000 W
PLW12	0.20863999 W
PLW13	0.10495000 W

F2 - Processing parameters

SI		32768
SF		75.4803210 MHz
WDW		EM
SSB	0	
LB		1.00 Hz
GB	0	
PC		1.40

Data File: C:\LabSolutions\Data\Analiz\uac\TU-35A_49.lcd

Elmt	Val.	Min	Max	Elmt	Val.	Min	Max	Elmt	Val.	Min	Max	Elmt	Val.	Min	Max	Use Adduct
H	1	9	40	O	2	1	1	S	2	0	2	Ru	2	0	0	H
C	4	11	26	F	1	0	0	Cl	1	0	1	I	3	0	0	
N	3	0	6	P	3	0	0	Br	1	0	1					

Error Margin (ppm): 5
HC Ratio: unlimited
Max Isotopes: 3
MSn Iso RI (%): 10.00

DBE Range: 17.0 - 20.0
Apply N Rule: yes
Isotope RI (%): 1.00
MSn Logic Mode: AND

Electron Ions: both
Use MSn Info: no
Isotope Res: 10000
Max Results: 500

Event#: 1 MS(E+) Ret. Time : 8.707 -> 8.787 - 8.947 -> 10.345 Scan# : 1307 -> 1319 - 1343 -> 1553

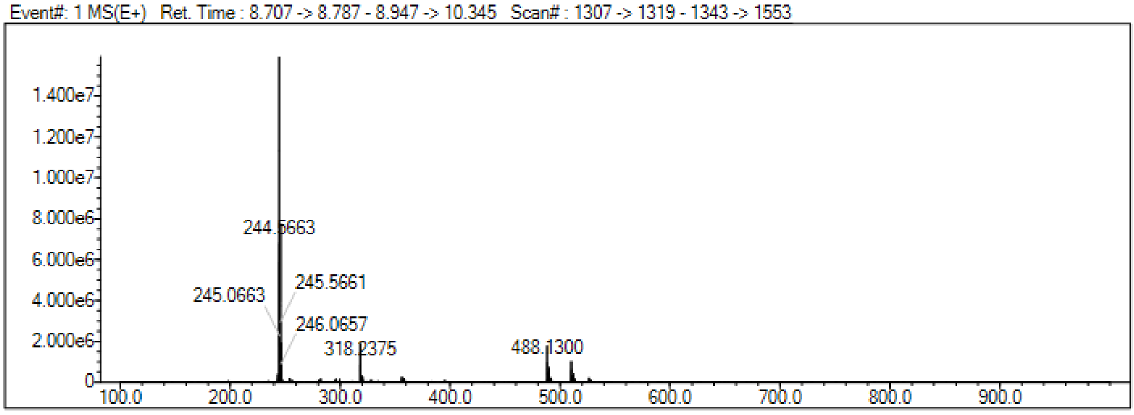

Measured region for 488.1300 m/z

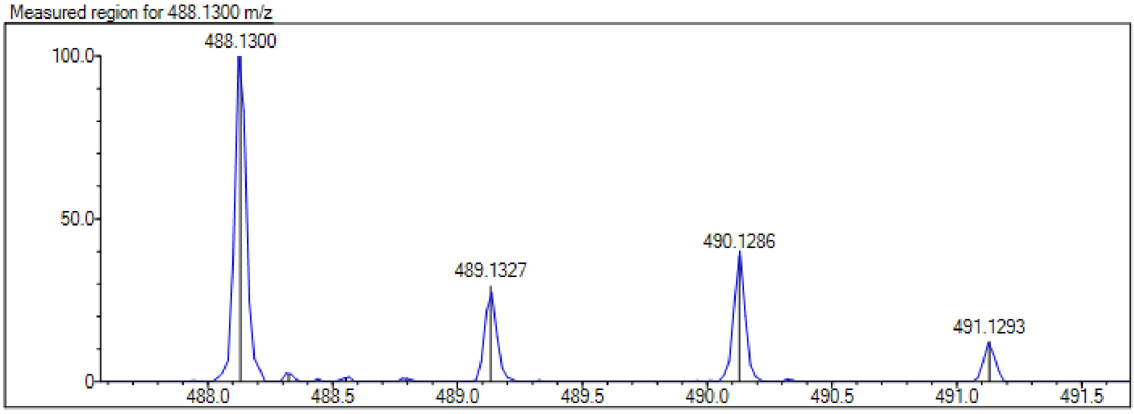

C26 H22 N5 O S Cl [M+H]+ : Predicted region for 488.1306 m/z

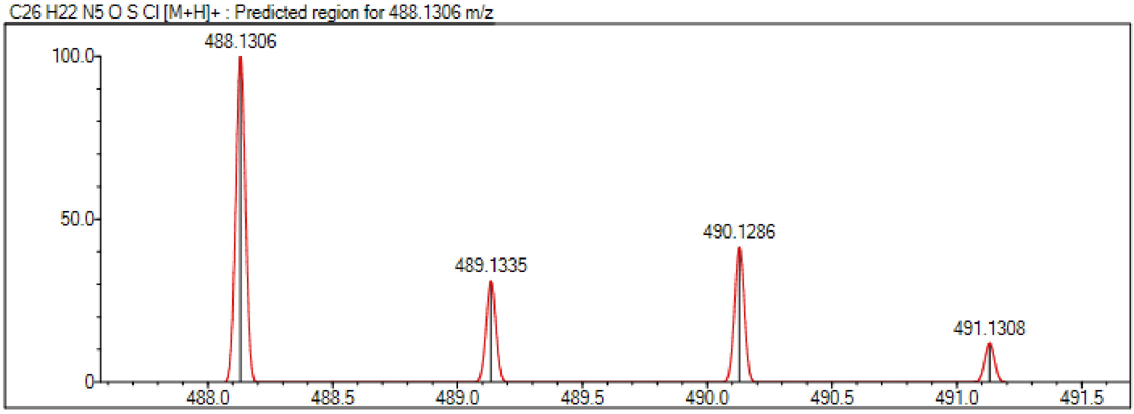

Rank	Score	Formula (M)	Ion	Meas. m/z	Pred. m/z	Df. (mDa)	Df. (ppm)	Iso	DBE
1	84.40	C26 H22 N5 O S Cl	[M+H]+	488.1300	488.1306	-0.6	-1.23	84.89	18.0

2-(4-(4-Ethyl-5-(2-(3,4-dihydroxyphenyl)-2-oxo-ethylthio)-4H-1,2,4-triazol-3yl)-phenyl)-5(6)-chloro-1H- benzimidazole (3h)

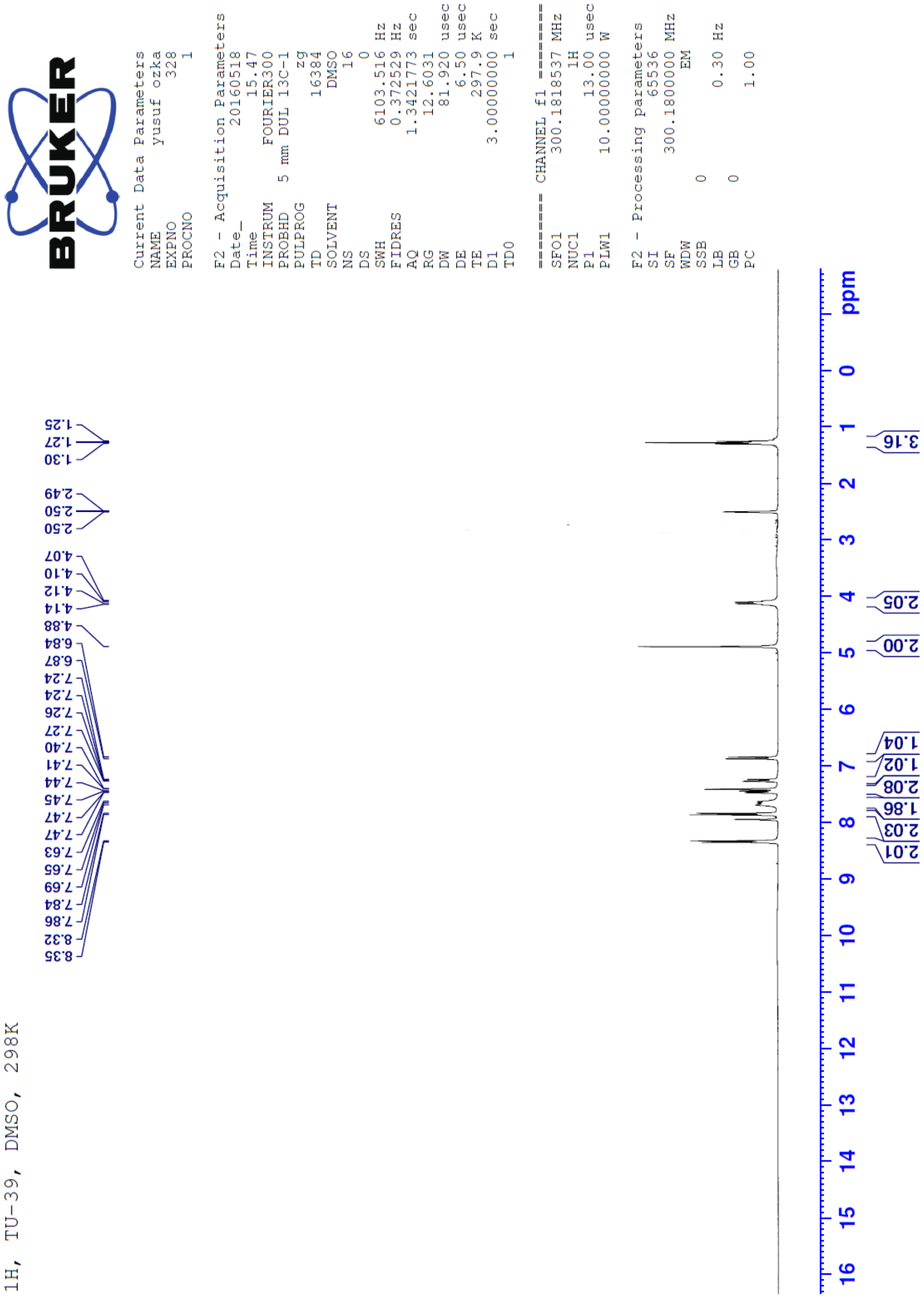

13C, TU-39, DMSO, 298K

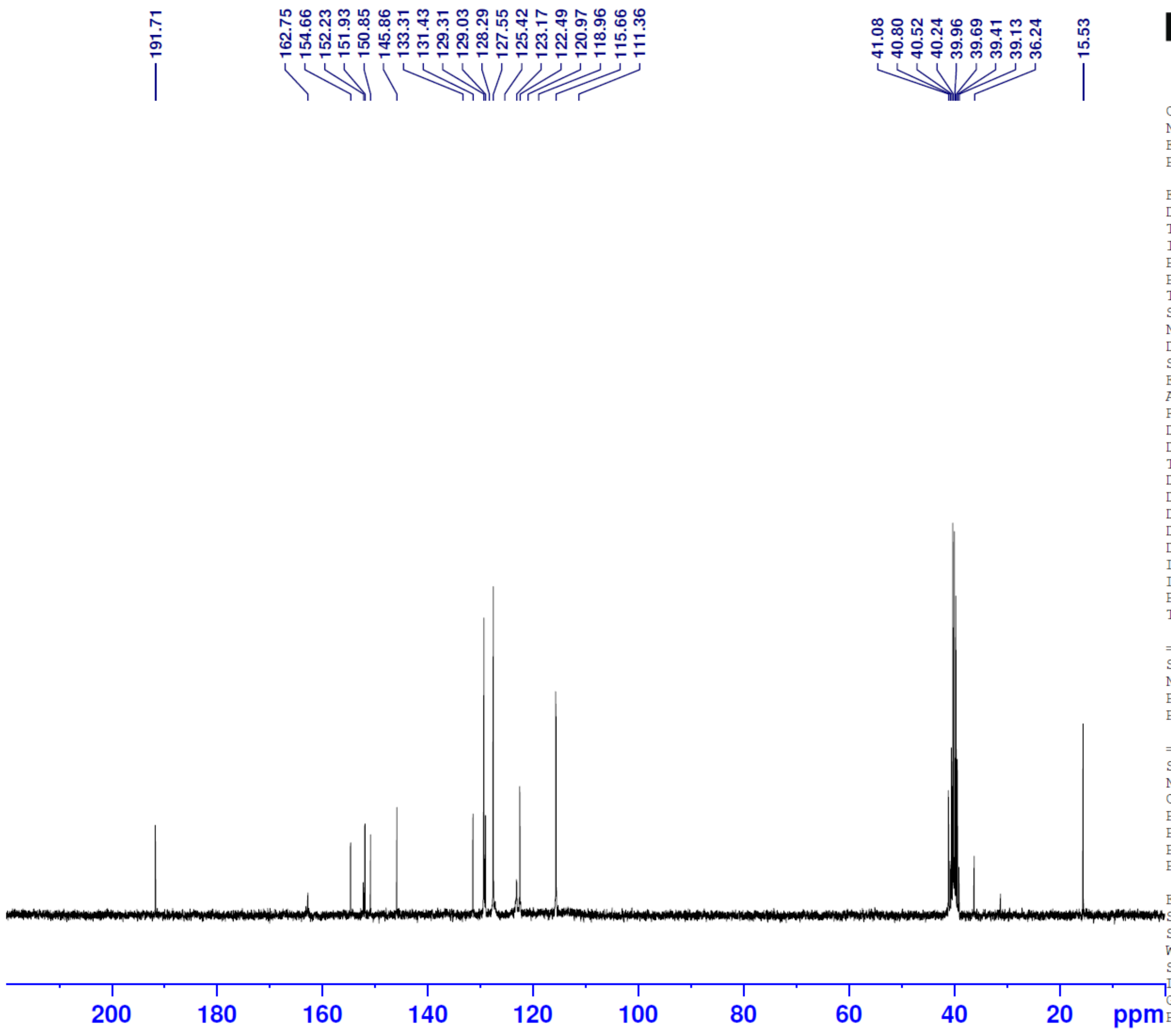

Current Data Parameters
NAME yusuf ozka
EXPNO 329
PROCNO 1

F2 - Acquisition Parameters
Date_ 20160518
Time 15.49
INSTRUM FOURIER300
PROBHD 5 mm DUL 13C-1
PULPROG zgpg
TD 32768
SOLVENT DMSO
NS 2048
DS 4
SWH 24414.063 Hz
FIDRES 0.745058 Hz
AQ 0.6710886 sec
RG 501.187
DW 20.480 usec
DE 6.50 usec
TE 297.9 K
D1 1.00000000 sec
D11 0.03000000 sec
D31 0.00001500 sec
D32 0.89999998 sec
D40 0.00093990 sec
L4 23
L5 26
P32 90.00 usec
TD0 1

======== CHANNEL f1 ========
SFO1 75.4878687 MHz
NUC1 13C
P1 15.00 usec
PLW1 15.00000000 W

======== CHANNEL f2 ========
SFO2 300.1812007 MHz
NUC2 1H
CPDPRG[2 waltz16
PCPD2 90.00 usec
PLW2 10.00000000 W
PLW12 0.20863999 W
PLW13 0.10495000 W

F2 - Processing parameters
SI 32768
SF 75.4803210 MHz
WDW EM
SSB 0
LB 1.00 Hz
GB 0
PC 1.40

Data File: C:\LabSolutions\Data\Analiz\uac\TU-39_56.lcd

Elmt	Val.	Min	Max	Elmt	Val.	Min	Max	Elmt	Val.	Min	Max	Elmt	Val.	Min	Max	Use Adduct
H	1	9	40	O	2	1	3	S	2	0	2	Ru	2	0	0	H
C	4	11	26	F	1	0	0	Cl	1	0	1	I	3	0	0	
N	3	0	6	P	3	0	0	Br	1	0	1					

Error Margin (ppm): 5
HC Ratio: unlimited
Max Isotopes: 3
MSn Iso RI (%): 10.00

DBE Range: 17.0 - 20.0
Apply N Rule: yes
Isotope RI (%): 1.00
MSn Logic Mode: AND

Electron Ions: both
Use MSn Info: no
Isotope Res: 10000
Max Results: 500

Event#: 1 MS(E+) Ret. Time : 6.333 -> 6.867 - 7.680 -> 9.066 Scan# : 951 -> 1031 - 1153 -> 1361

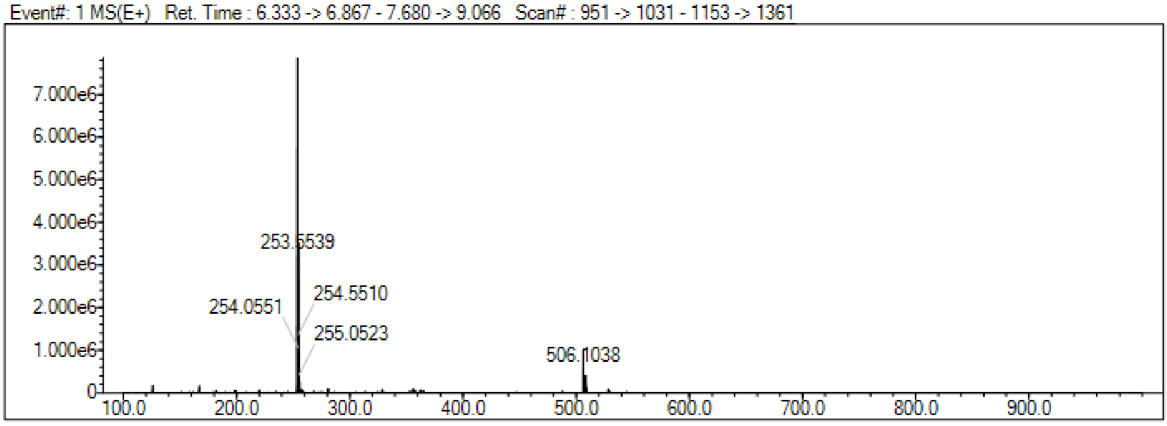

Measured region for 506.1038 m/z

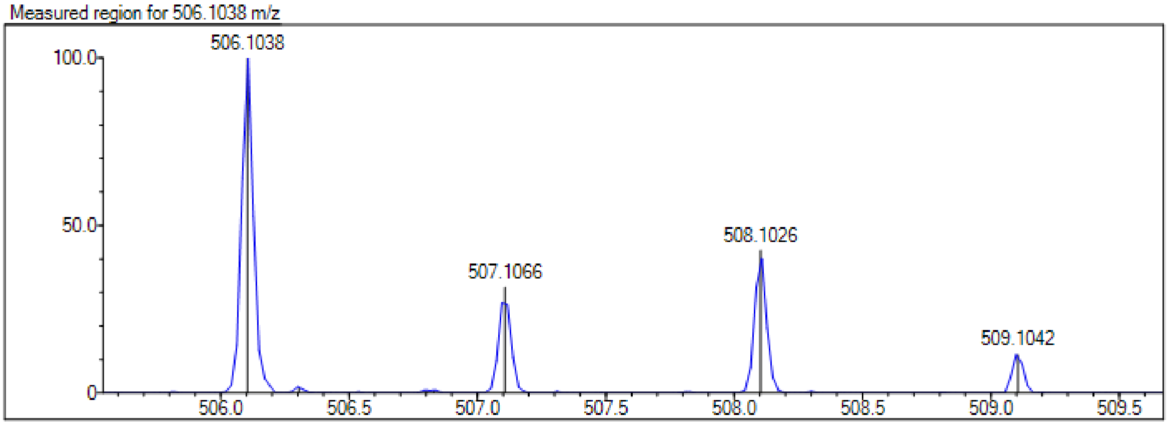

C25 H20 N5 O3 S Cl [M+H]+ : Predicted region for 506.1048 m/z

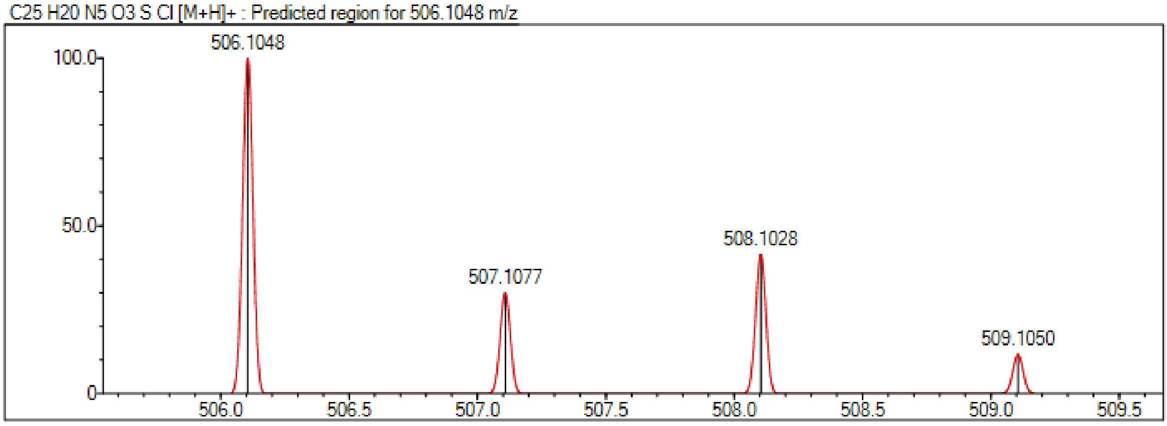

Rank	Score	Formula (M)	Ion	Meas. m/z	Pred. m/z	Df. (mDa)	Df. (ppm)	Iso	DBE
1	93.72	C25 H20 N5 O3 S Cl	[M+H]+	506.1038	506.1048	-1.0	-1.98	96.08	18.0

2-(4-(4-Methyl-5-(2-(4-cyanophenyl)-2-oxo-ethylthio)-4H-1,2,4-triazol-3yl)-phenyl)-5(6)-fluoro-1H-benzoimidazole (3i)

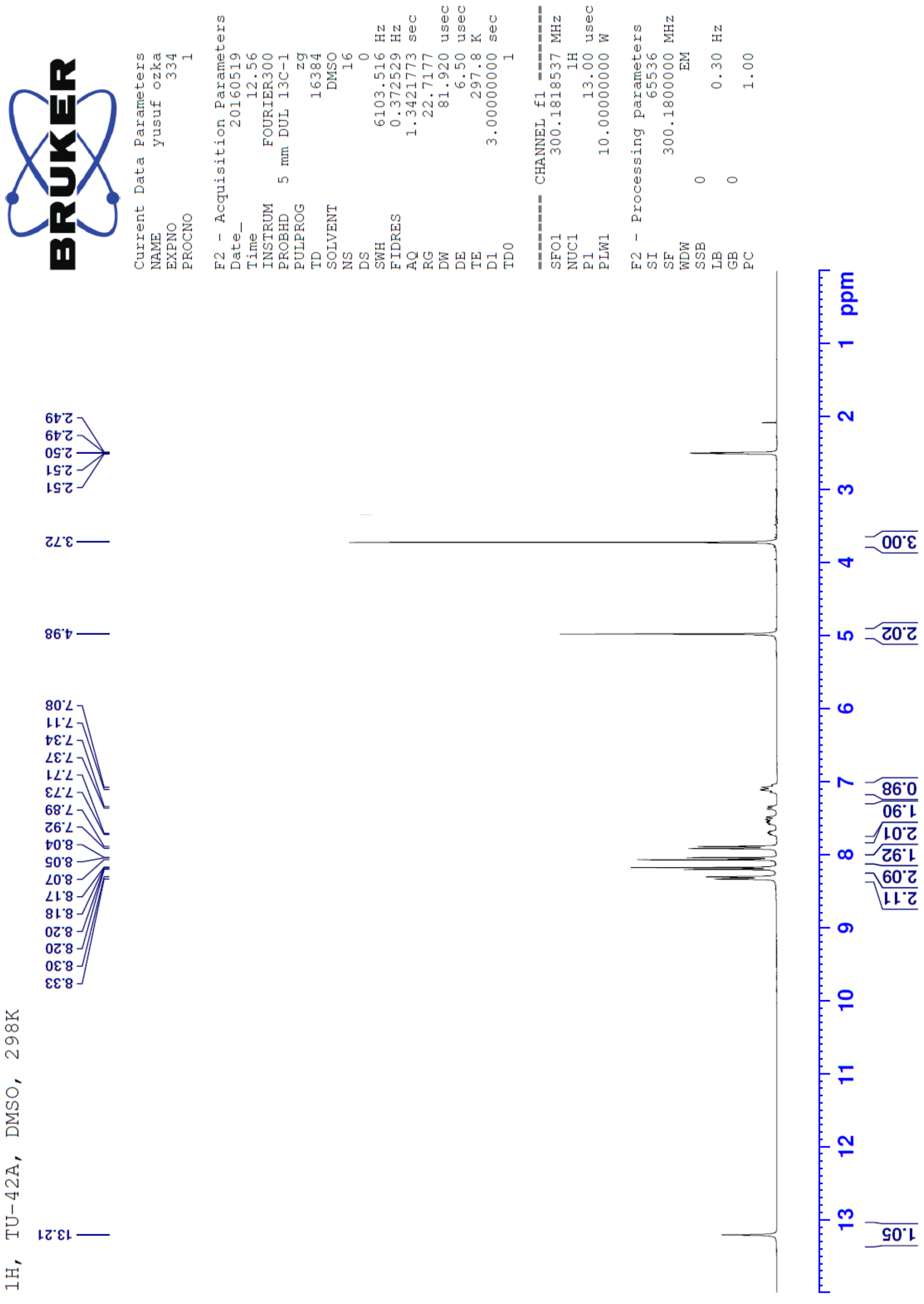

13C, TU-42A, DMSO, 298K

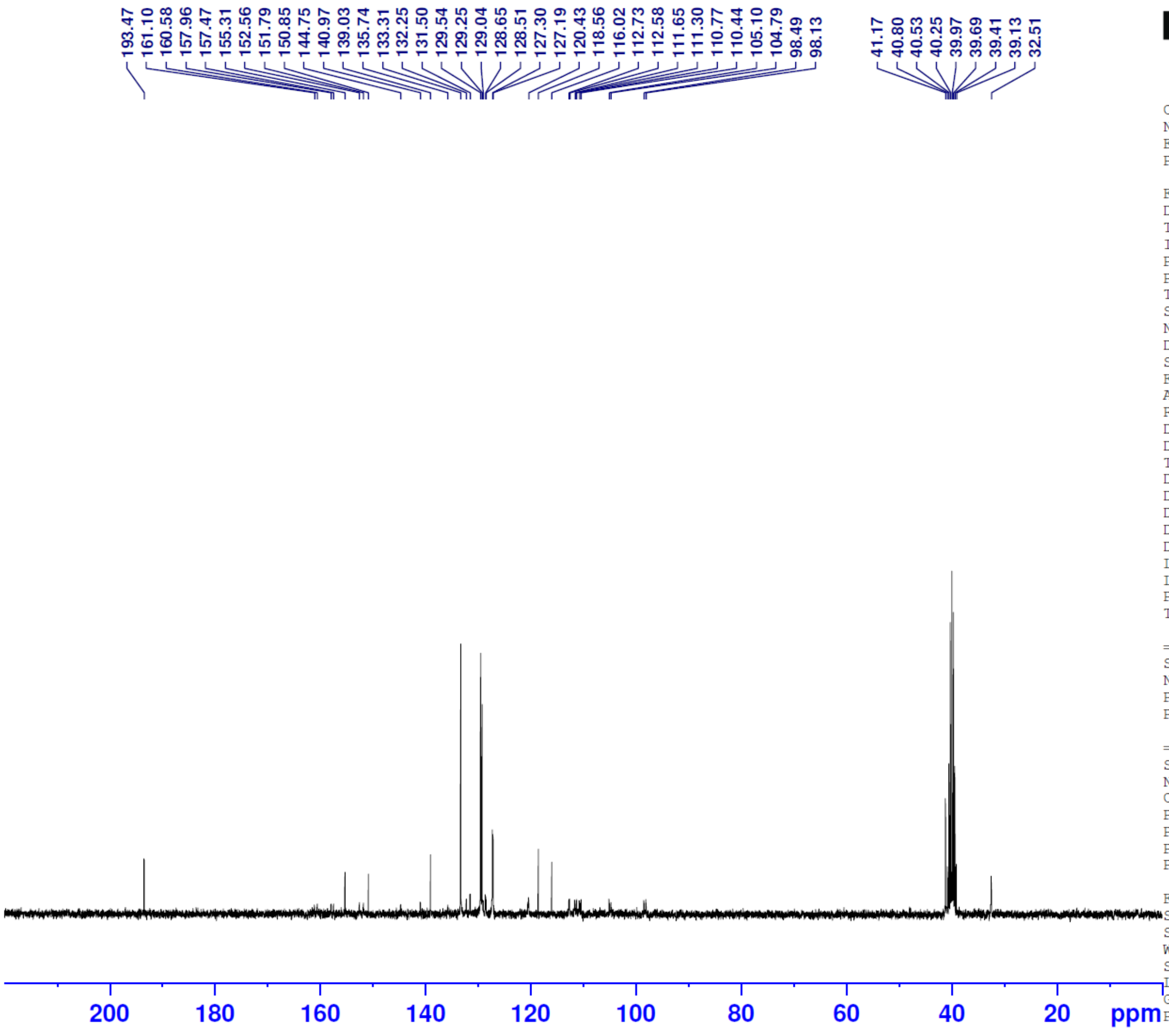

BRUKER

```
Current Data Parameters
NAME            yusuf ozka
EXPNO                  335
PROCNO                   1

F2 - Acquisition Parameters
Date_             20160519
Time                 12.58
INSTRUM         FOURIER300
PROBHD    5 mm DUL 13C-1
PULPROG               zgpg
TD                   32768
SOLVENT               DMSO
NS                    2048
DS                       4
SWH              24414.063 Hz
FIDRES            0.745058 Hz
AQ               0.6710886 sec
RG                 501.187
DW                  20.480 usec
DE                    6.50 usec
TE                   298.0 K
D1              1.00000000 sec
D11             0.03000000 sec
D31             0.00001500 sec
D32             0.89999998 sec
D40             0.00093990 sec
L4                      23
L5                      26
P32                  90.00 usec
TD0                      1

======== CHANNEL f1 ========
SFO1            75.4878687 MHz
NUC1                   13C
P1                   15.00 usec
PLW1           15.00000000 W

======== CHANNEL f2 ========
SFO2           300.1812007 MHz
NUC2                    1H
CPDPRG[2           waltz16
PCPD2                90.00 usec
PLW2           10.00000000 W
PLW12           0.20863999 W
PLW13           0.10495000 W

F2 - Processing parameters
SI                   32768
SF              75.4803210 MHz
WDW                     EM
SSB      0
LB                    1.00 Hz
GB       0
PC                    1.40
```

Data File: C:\LabSolutions\Data\Analiz\uac\TU-42A_59.lcd

Elmt	Val.	Min	Max	Elmt	Val.	Min	Max	Elmt	Val.	Min	Max	Elmt	Val.	Min	Max	Use Adduct
H	1	9	40	O	2	1	3	S	2	0	2	Ru	2	0	0	H
C	4	11	26	F	1	0	1	Cl	1	0	1	I	3	0	0	
N	3	0	6	P	3	0	0	Br	1	0	1					

Error Margin (ppm): 5
HC Ratio: unlimited
Max Isotopes: 3
MSn Iso RI (%): 10.00

DBE Range: 17.0 - 20.0
Apply N Rule: yes
Isotope RI (%): 1.00
MSn Logic Mode: AND

Electron Ions: both
Use MSn Info: no
Isotope Res: 10000
Max Results: 500

Event#: 1 MS(E+) Ret. Time : 6.400 -> 6.840 - 7.720 -> 9.087 Scan# : 961 -> 1027 - 1159 -> 1365

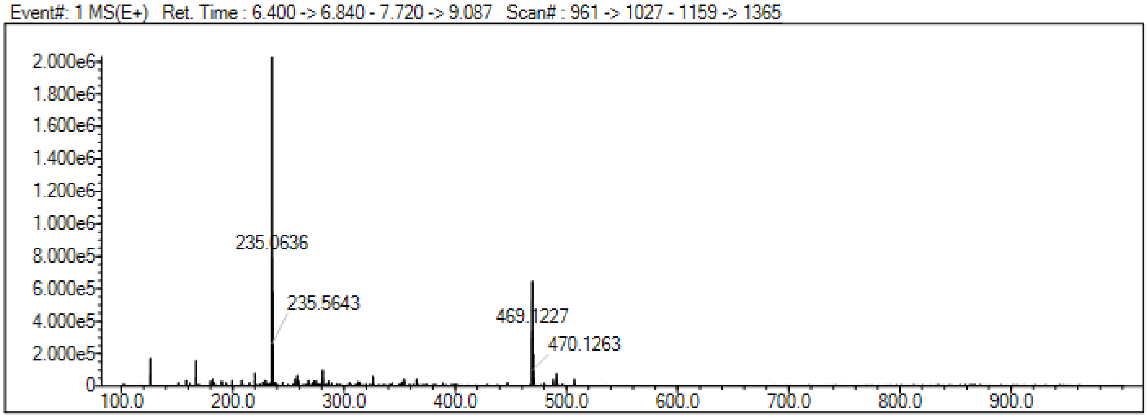

Measured region for 469.1227 m/z

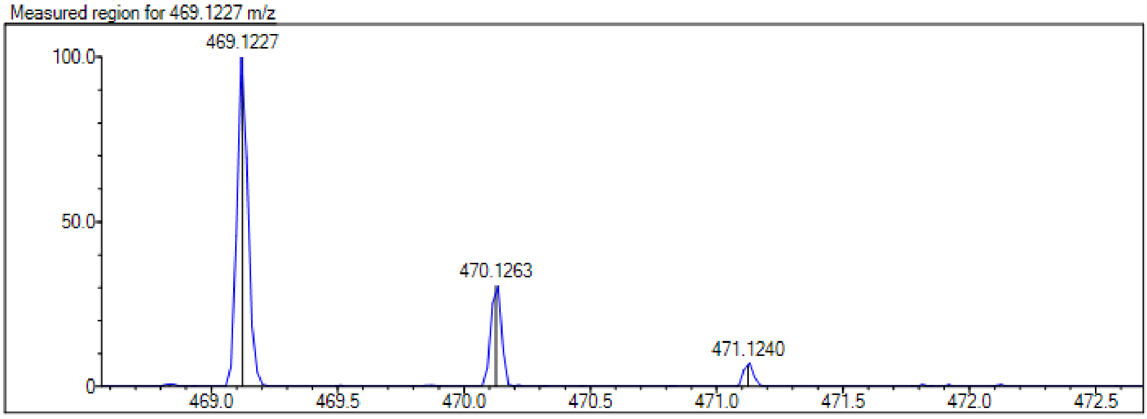

C25 H17 N6 O F S [M+H]+ : Predicted region for 469.1241 m/z

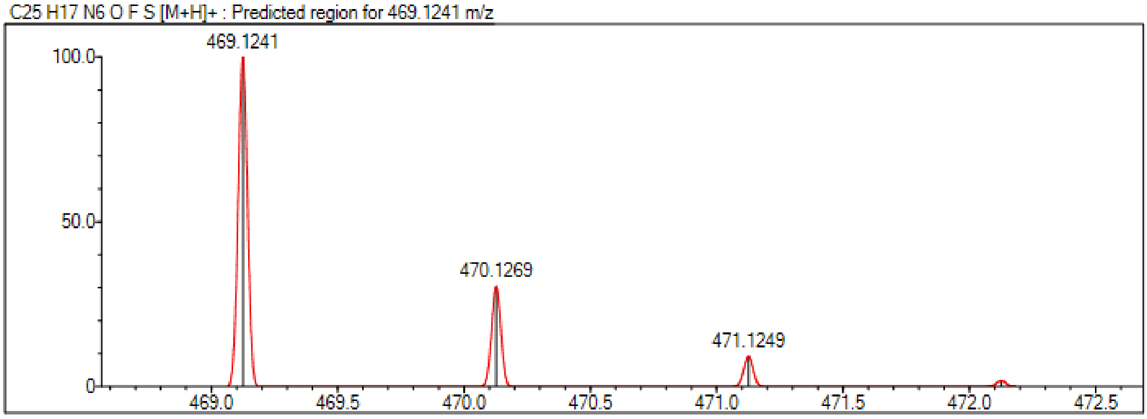

Rank	Score	Formula (M)	Ion	Meas. m/z	Pred. m/z	Df. (mDa)	Df. (ppm)	Iso	DBE
1	73.31	C25 H17 N6 O F S	[M+H]+	469.1227	469.1241	-1.4	-2.98	77.13	20.0

2-(4-(4-Methyl-5-(2-(4-bromophenyl)-2-oxo-ethylthio)-4H-1,2,4-triazol-3yl)-phenyl)-5(6)-fluoro-1H-benzimidazole (3j)

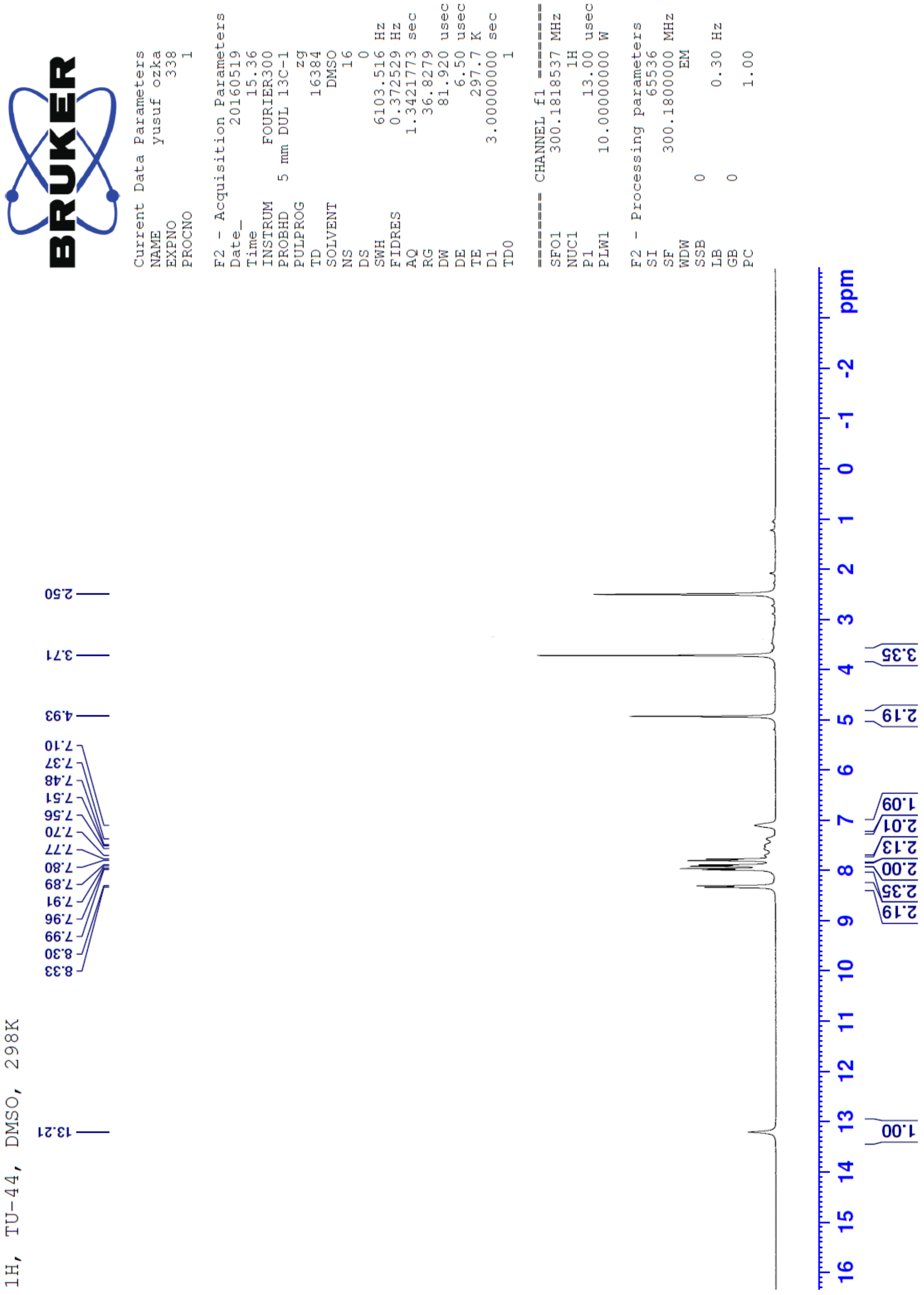

13C, TU-44, DMSO, 298K

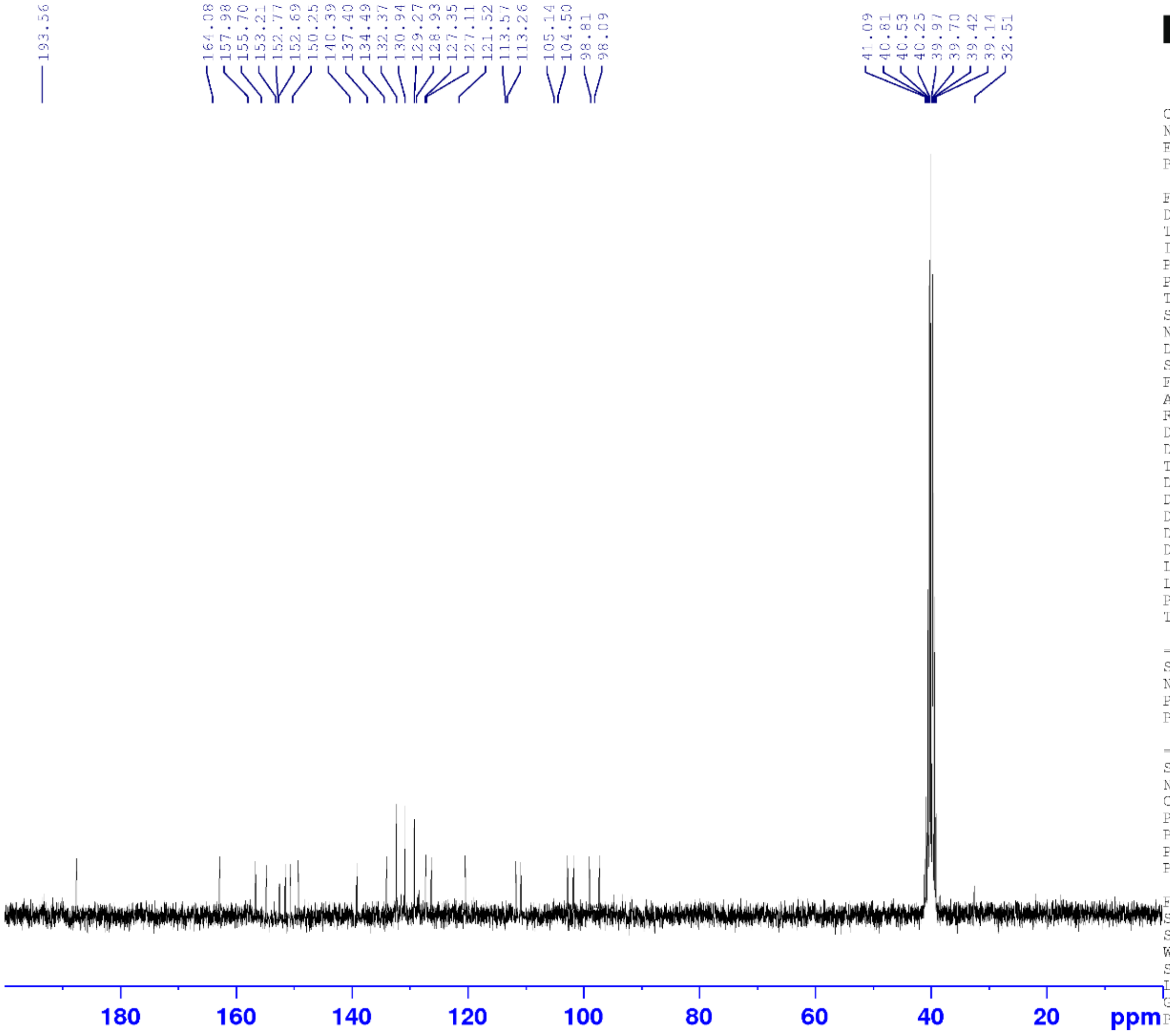

Current Data Parameters
NAME Yusuf Ozkay
EXPNO 339
PROCNO 1

F2 - Acquisition Parameters
Date_ 20160519
Time 15.38
INSTRUM FOURIER300
PROBHD 5 mm DUL 13C-1
PULPROG zgpg
TD 32768
SOLVENT DMSO
NS 2048
DS 4
SWH 24414.063 Hz
FIDRES 0.745058 Hz
AQ 0.6710886 sec
RG 501.187
DW 20.480 usec
DE 6.50 usec
TE 298.0 K
D1 1.00000000 sec
D11 0.03000000 sec
D31 0.00001500 sec
D32 0.89999998 sec
D40 0.00093990 sec
L4 23
L5 26
P32 90.00 usec
TD0 1

======== CHANNEL f1 ========
SFO1 75.4878687 MHz
NUC1 13C
P1 15.00 usec
PLW1 15.00000000 W

======== CHANNEL f2 ========
SFO2 300.1812007 MHz
NUC2 1H
CPDPRG[2 waltz16
PCPD2 90.00 usec
PLW2 10.00000000 W
PLW12 0.20863999 W
PLW13 0.10495000 W

F2 - Processing parameters
SI 32768
SF 75.4803210 MHz
WDW EM
SSB 0
LB 1.00 Hz
GB 0
PC 1.40

Data File: C:\LabSolutions\Data\Analiz\uac\TU-44A_62.lcd

Elmt	Val.	Min	Max	Elmt	Val.	Min	Max	Elmt	Val.	Min	Max	Elmt	Val.	Min	Max	Use Adduct
H	1	9	40	O	2	1	3	S	2	0	2	Ru	2	0	0	H
C	4	11	26	F	1	0	1	Cl	1	0	0	I	3	0	0	
N	3	0	6	P	3	0	0	Br	1	0	1					

Error Margin (ppm): 5
HC Ratio: unlimited
Max Isotopes: 3
MSn Iso RI (%): 10.00

DBE Range: 17.0 - 20.0
Apply N Rule: yes
Isotope RI (%): 1.00
MSn Logic Mode: AND

Electron Ions: both
Use MSn Info: no
Isotope Res: 10000
Max Results: 500

Event#: 1 MS(E+) Ret. Time : 7.067 -> 7.440 - 9.747 -> 10.615 Scan# : 1061 -> 1117 - 1463 -> 1593

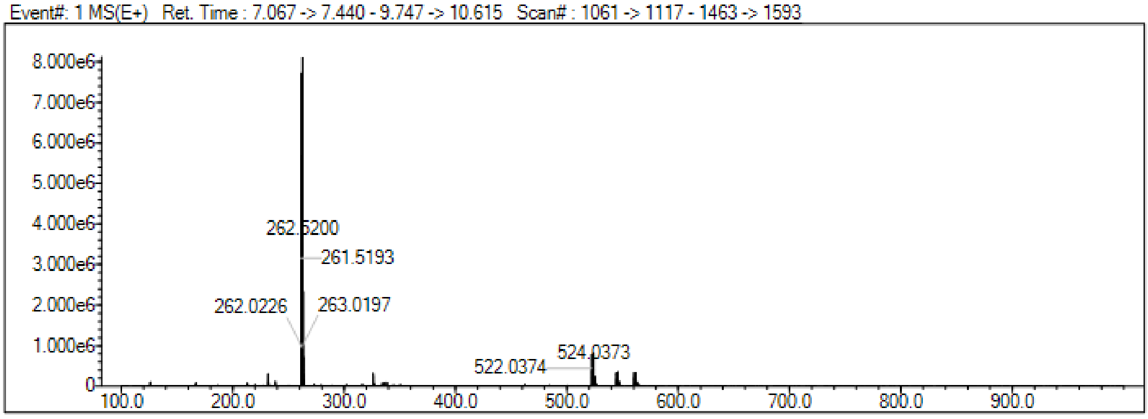

Measured region for 522.0374 m/z

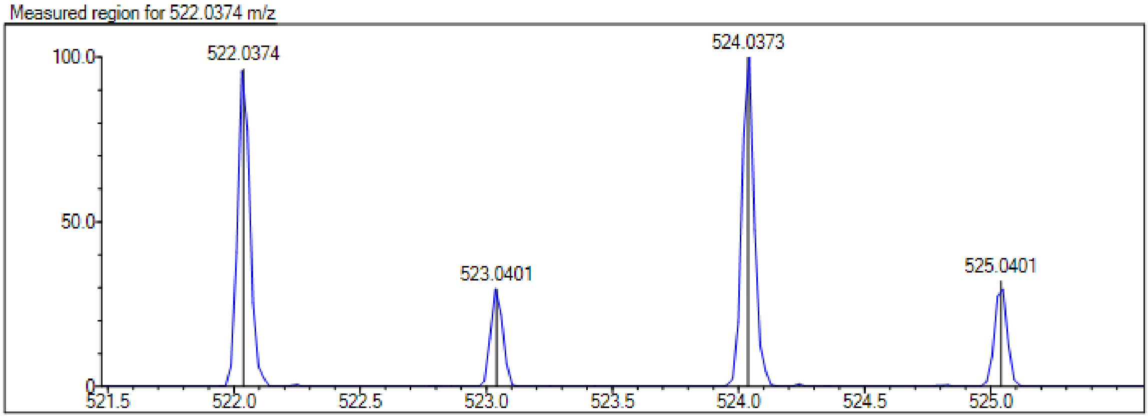

C24 H17 N5 O F S Br [M+H]+ : Predicted region for 522.0394 m/z

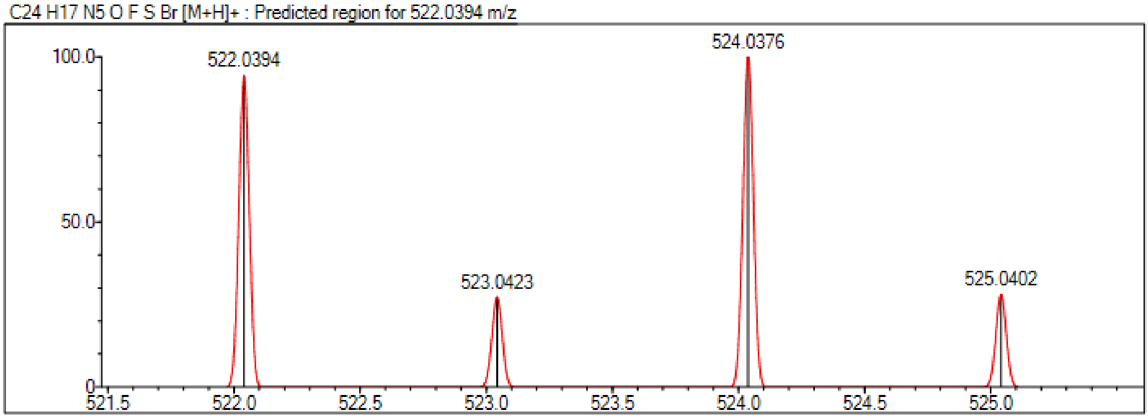

Rank	Score	Formula (M)	Ion	Meas. m/z	Pred. m/z	Df. (mDa)	Df. (ppm)	Iso	DBE
1	82.13	C24 H17 N5 O F S Br	[M+H]+	522.0374	522.0394	-2.0	-3.83	88.38	18.0

2-(4-(4-Methyl-5-(2-(4-methylphenyl)-2-oxo-ethylthio)-4H-1,2,4-triazol-3yl)-phenyl)-5(6)-fluoro-1H-benzimidazole (3k)

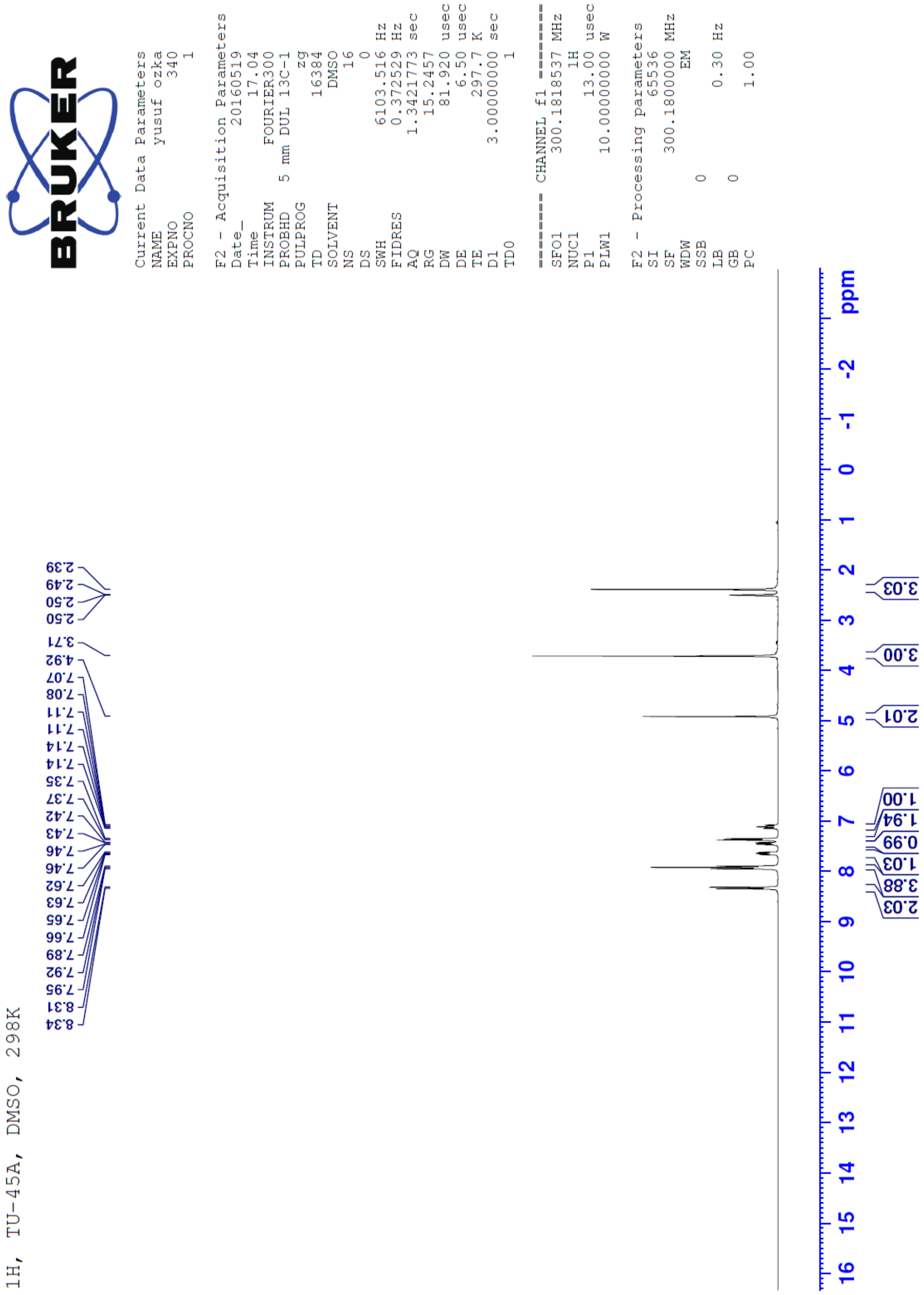

13C, TU-45A, DMSO, 298K

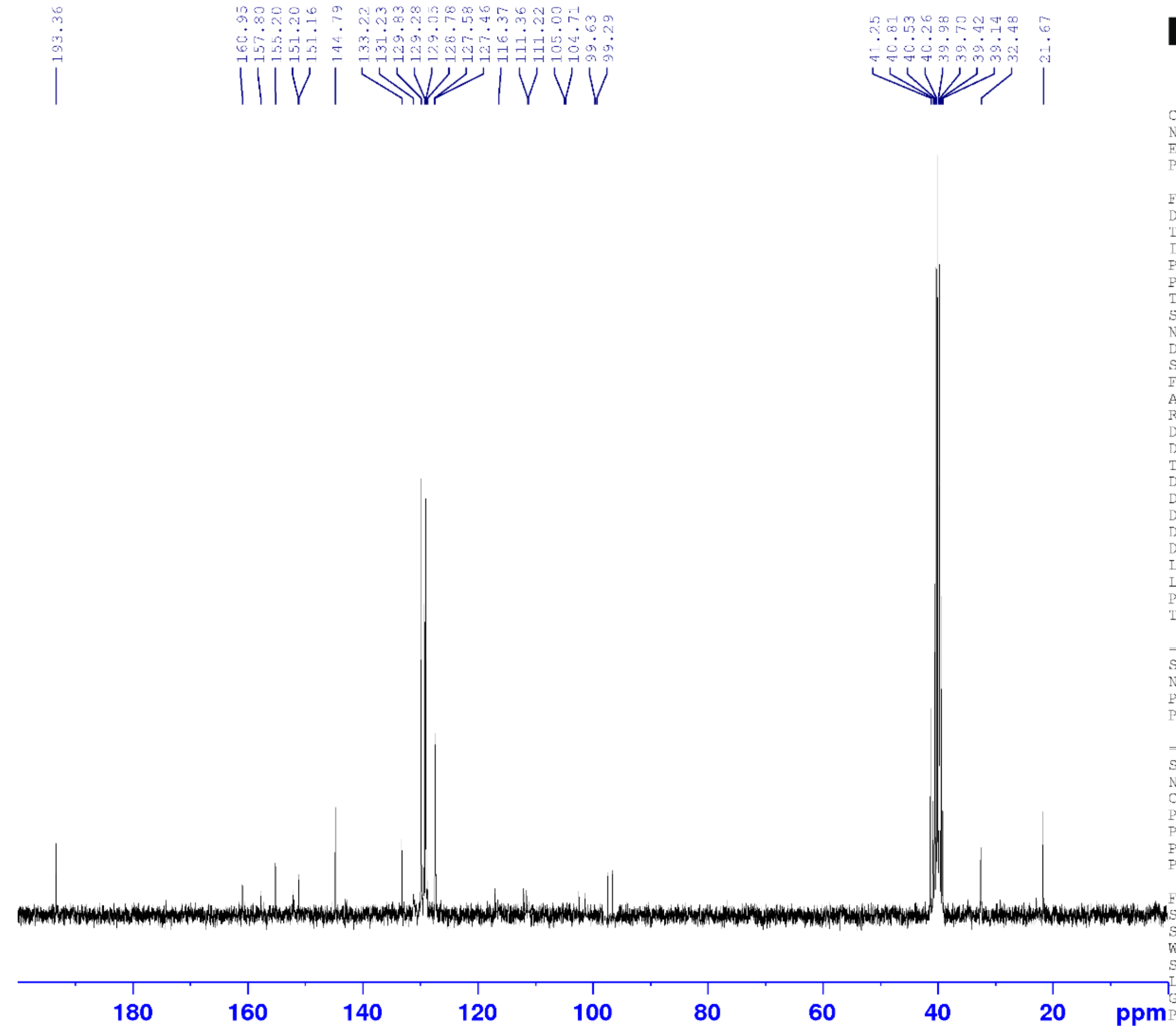

Current Data Parameters	
NAME	Yusuf Ozkay
EXPNO	341
PROCNO	1
**F2 - Acquisition Parameters**	
Date_	20160519
Time	17.05
INSTRUM	FOURIER300
PROBHD	5 mm DUL 13C-1
PULPROG	zgpg
TD	32768
SOLVENT	DMSO
NS	2048
DS	4
SWH	24414.063 Hz
FIDRES	0.745058 Hz
AQ	0.6710886 sec
RG	501.187
DW	20.480 usec
DE	6.50 usec
TE	297.8 K
D1	1.00000000 sec
D11	0.03000000 sec
D31	0.00001500 sec
D32	0.89999998 sec
D40	0.00093990 sec
L4	23
L5	26
P32	90.00 usec
TD0	1
======== CHANNEL f1 ========	
SFO1	75.4878687 MHz
NUC1	13C
P1	15.00 usec
PLW1	15.00000000 W
======== CHANNEL f2 ========	
SFO2	300.1812007 MHz
NUC2	1H
CPDPRG[2	waltz16
PCPD2	90.00 usec
PLW2	10.00000000 W
PLW12	0.20863999 W
PLW13	0.10495000 W
**F2 - Processing parameters**	
SI	32768
SF	75.4803210 MHz
WDW	EM
SSB	0
LB	1.00 Hz
GB	0
PC	1.40

Data File: C:\LabSolutions\Data\Analiz\uac\TU-45A_64.lcd

Elmt	Val.	Min	Max	Elmt	Val.	Min	Max	Elmt	Val.	Min	Max	Elmt	Val.	Min	Max	Use Adduct
H	1	9	40	O	2	1	3	S	2	0	2	Ru	2	0	0	H
C	4	11	26	F	1	0	1	Cl	1	0	0	I	3	0	0	
N	3	0	6	P	3	0	0	Br	1	0	1					

Error Margin (ppm):	5	DBE Range:	17.0 - 20.0	Electron Ions:	both
HC Ratio:	unlimited	Apply N Rule:	yes	Use MSn Info:	no
Max Isotopes:	3	Isotope RI (%):	1.00	Isotope Res:	10000
MSn Iso RI (%):	10.00	MSn Logic Mode:	AND	Max Results:	500

Event#: 1 MS(E+) Ret. Time : 6.573 -> 6.800 - 7.253 -> 8.931 Scan# : 987 -> 1021 - 1089 -> 1341

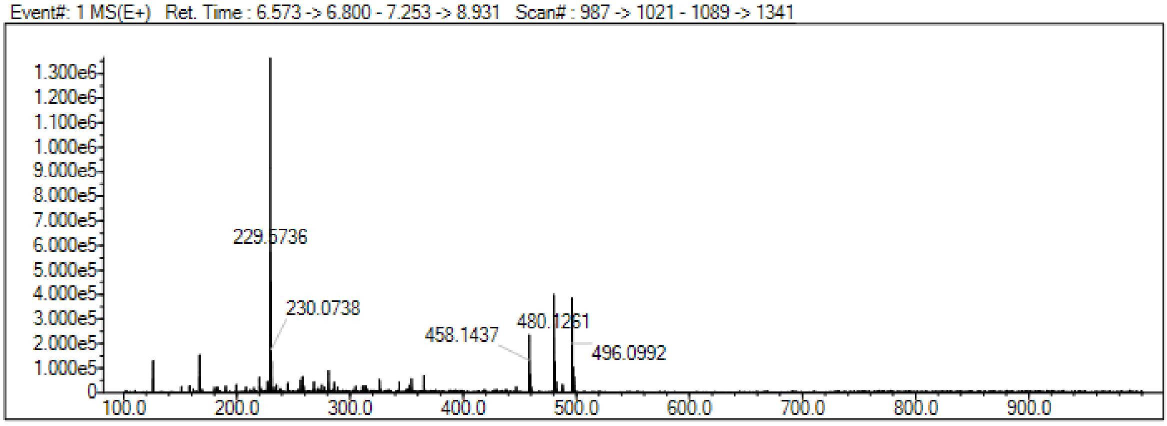

Measured region for 458.1437 m/z

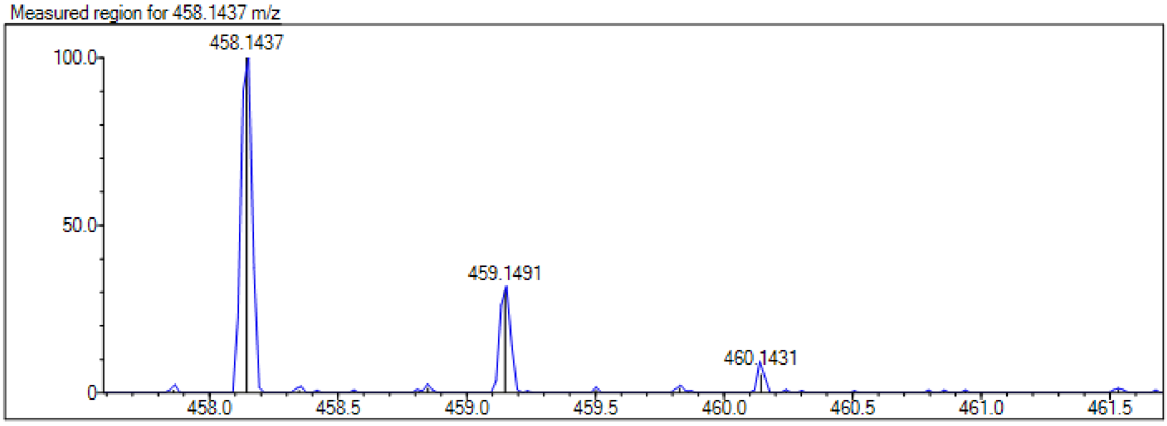

C25 H20 N5 O F S [M+H]+ : Predicted region for 458.1445 m/z

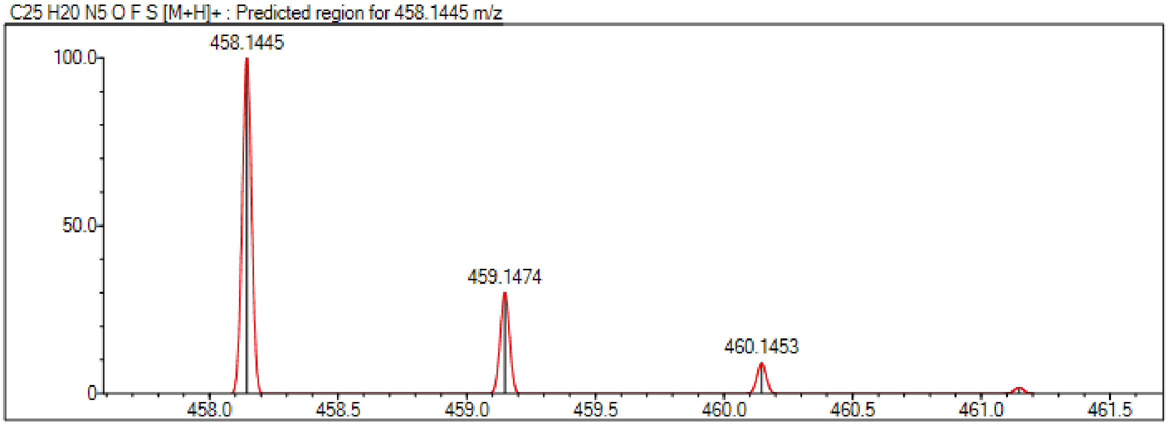

Rank	Score	Formula (M)	Ion	Meas. m/z	Pred. m/z	Df. (mDa)	Df. (ppm)	Iso	DBE
1	87.11	C25 H20 N5 O F S	[M+H]+	458.1437	458.1445	-0.8	-1.75	88.77	18.0

2-(4-(4-Methyl-5-(2-(3,4-dihydroxyphenyl)-2-oxo-ethylthio)-4H-1,2,4-triazol-3yl)-phenyl)-5(6)-fluoro-1H- benzimidazole (3l)

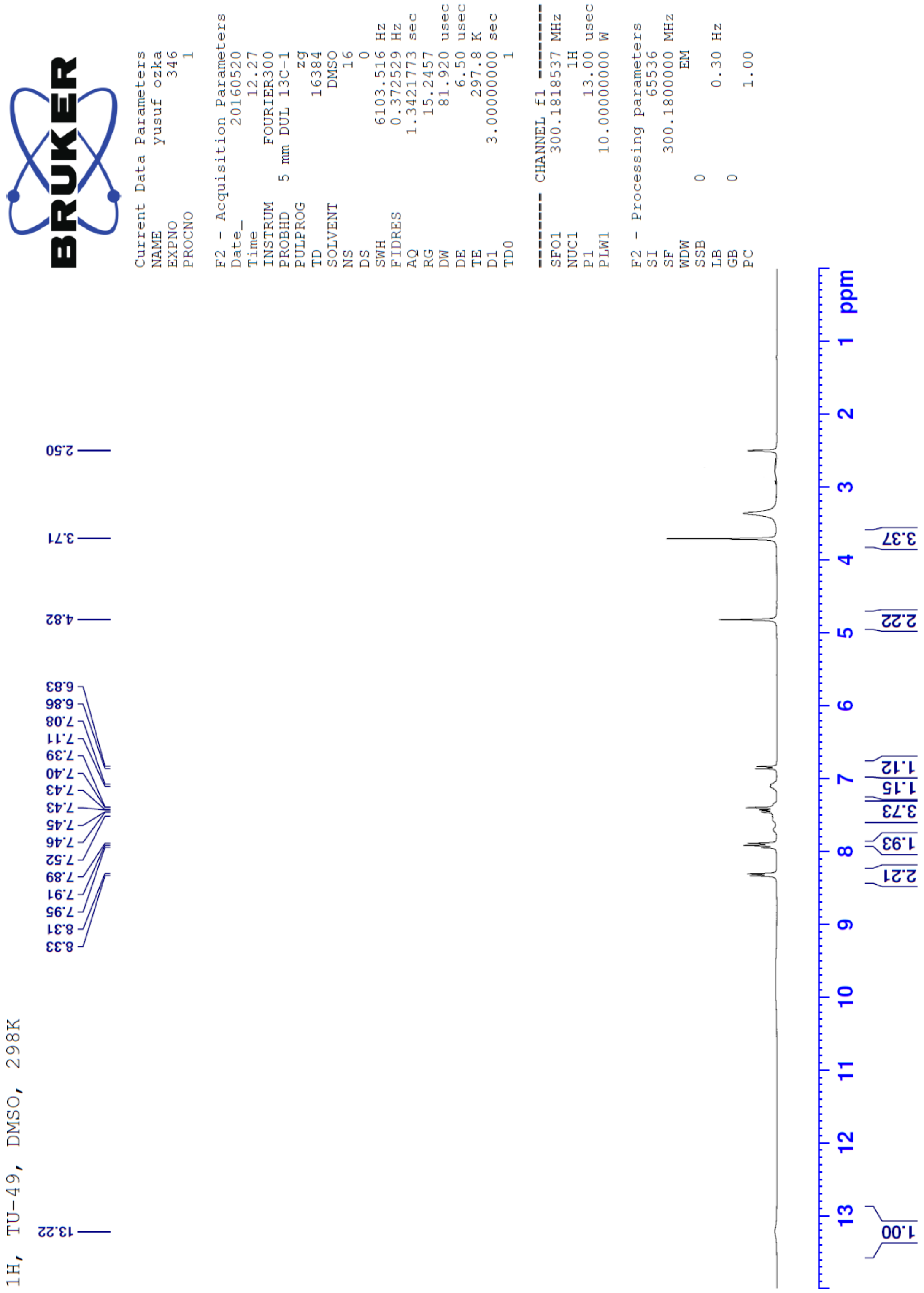

13C, TU-49, DMSO, 298K

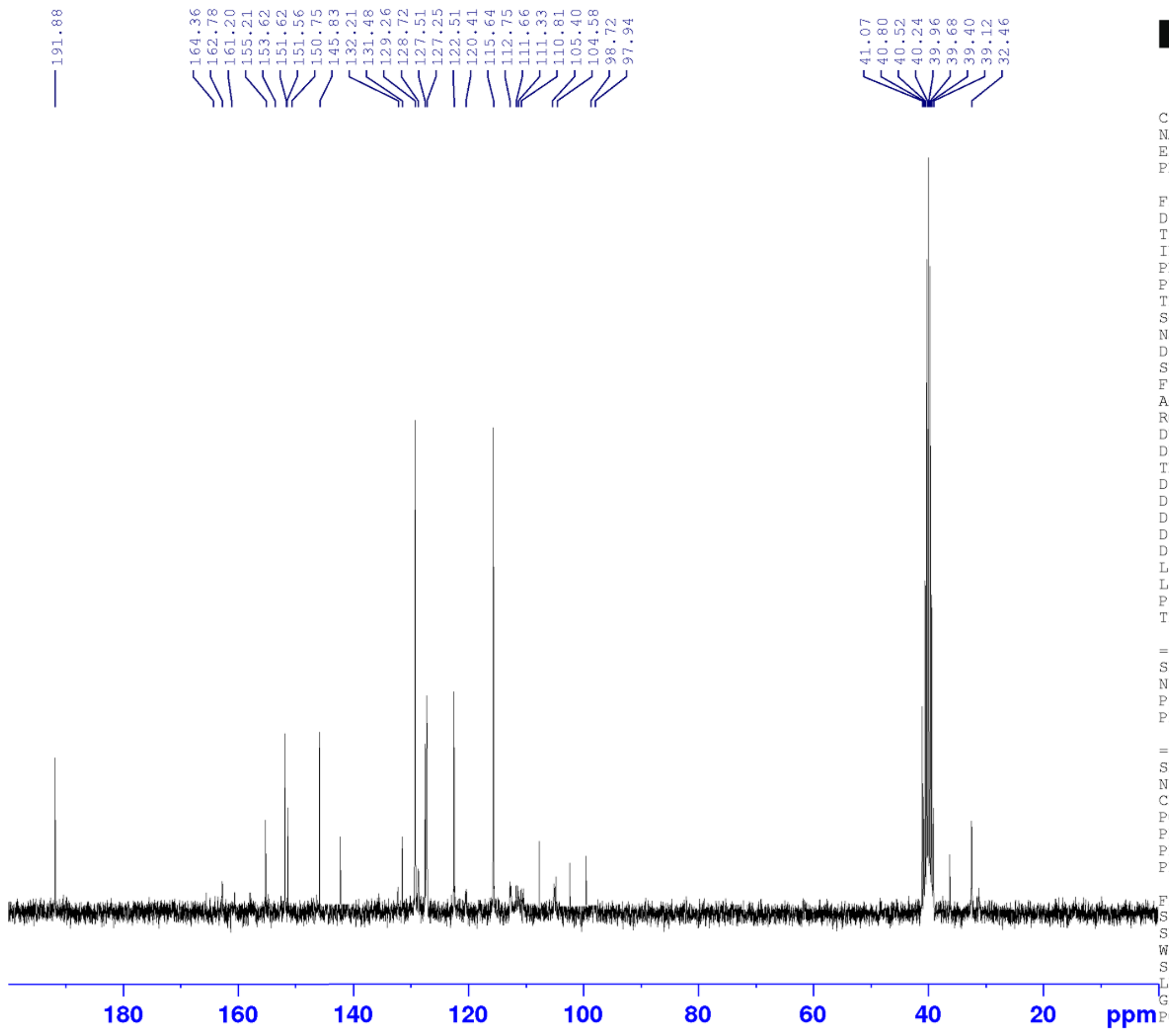

Current Data Parameters	
NAME	Yusuf Ozkay
EXPNO	347
PROCNO	1
F2 - Acquisition Parameters	
Date_	20160520
Time	12.29
INSTRUM	FOURIER300
PROBHD	5 mm DUL 13C-1
PULPROG	zgpg
TD	32768
SOLVENT	DMSO
NS	2048
DS	4
SWH	24414.063 Hz
FIDRES	0.745058 Hz
AQ	0.6710886 sec
RG	501.187
DW	20.480 usec
DE	6.50 usec
TE	297.9 K
D1	1.00000000 sec
D11	0.03000000 sec
D31	0.00001500 sec
D32	0.89999998 sec
D40	0.00093990 sec
L4	23
L5	26
P32	90.00 usec
TD0	1
======== CHANNEL f1 ========	
SFO1	75.4878687 MHz
NUC1	13C
P1	15.00 usec
PLW1	15.00000000 W
======== CHANNEL f2 ========	
SFO2	300.1812007 MHz
NUC2	1H
CPDPRG[2	waltz16
PCPD2	90.00 usec
PLW2	10.00000000 W
PLW12	0.20863999 W
PLW13	0.10495000 W
F2 - Processing parameters	
SI	32768
SF	75.4803210 MHz
WDW	EM
SSB	0
LB	1.00 Hz
GB	0
PC	1.40

Data File: C:\LabSolutions\Data\Analiz\uac\TU-49_70.lcd

Elmt	Val.	Min	Max	Elmt	Val.	Min	Max	Elmt	Val.	Min	Max	Elmt	Val.	Min	Max	Use Adduct
H	1	9	40	O	2	1	3	S	2	0	2	Ru	2	0	0	H
C	4	11	26	F	1	0	1	Cl	1	0	0	I	3	0	0	
N	3	0	6	P	3	0	0	Br	1	0	1					

Error Margin (ppm): 5
HC Ratio: unlimited
Max Isotopes: 3
MSn Iso RI (%): 10.00

DBE Range: 17.0 - 20.0
Apply N Rule: yes
Isotope RI (%): 1.00
MSn Logic Mode: AND

Electron Ions: both
Use MSn Info: no
Isotope Res: 10000
Max Results: 500

Event#: 1 MS(E+) Ret. Time : 5.867 -> 6.253 - 8.347 -> 9.160 Scan# : 881 -> 939 - 1253 -> 1375

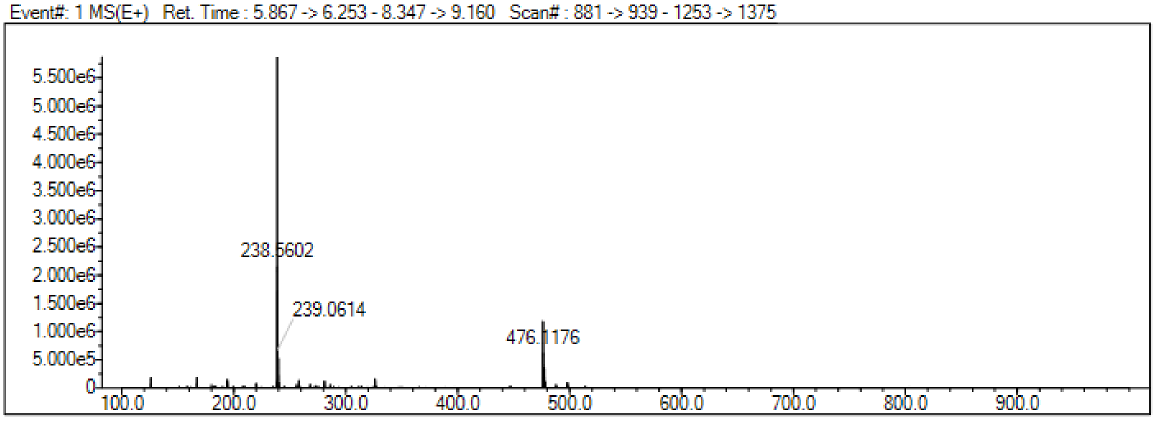

Measured region for 476.1176 m/z

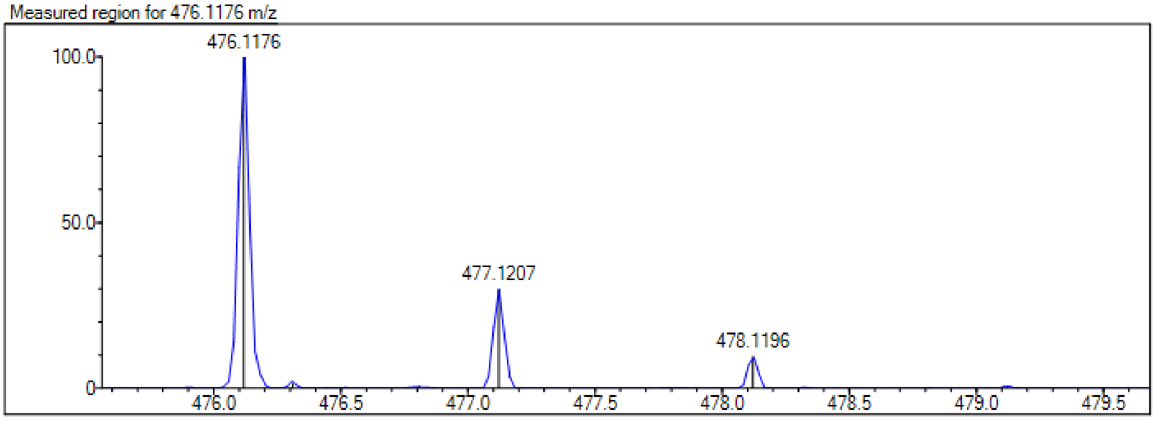

C24 H18 N5 O3 F S [M+H]+ : Predicted region for 476.1187 m/z

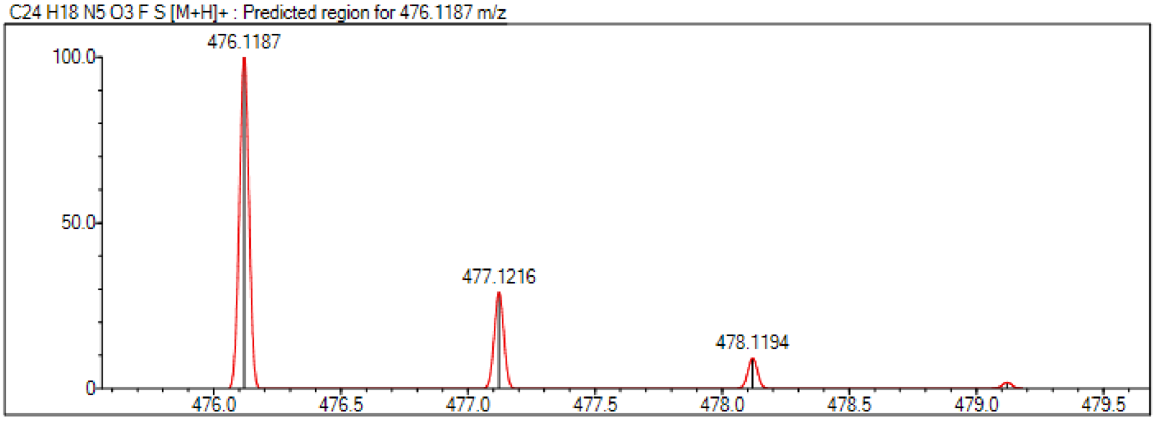

Rank	Score	Formula (M)	Ion	Meas. m/z	Pred. m/z	Df. (mDa)	Df. (ppm)	Iso	DBE
1	95.49	C24 H18 N5 O3 F S	[M+H]+	476.1176	476.1187	-1.1	-2.31	98.72	18.0

2-(4-(4-Ethyl-5-(2-(4-cyanophenyl)-2-oxo-ethylthio)-4H-1,2,4-triazol-3yl)-phenyl)-5(6)-fluoro-1H-benzoimidazole (3m)

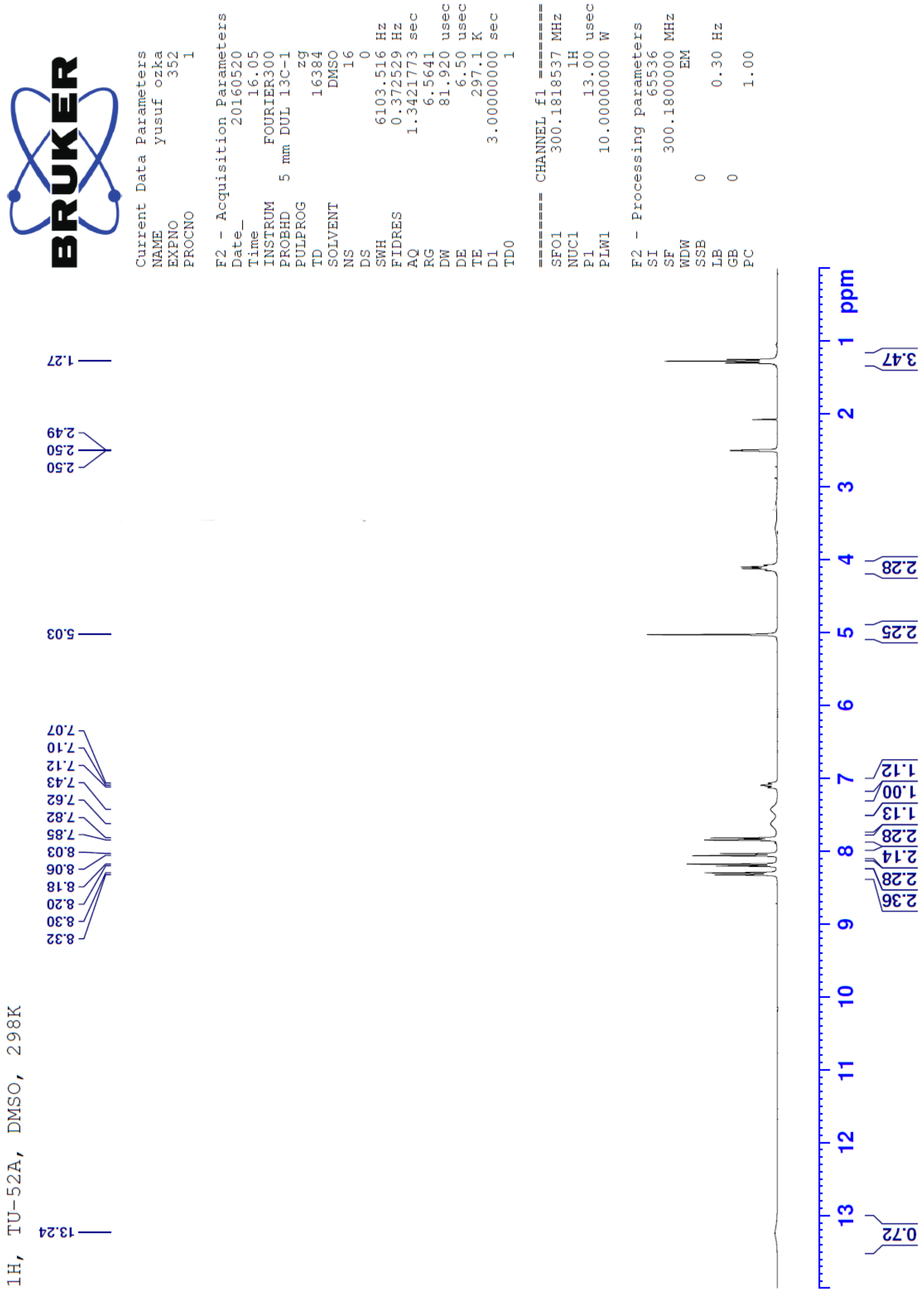

13C, TU-52A, DMSO, 298K

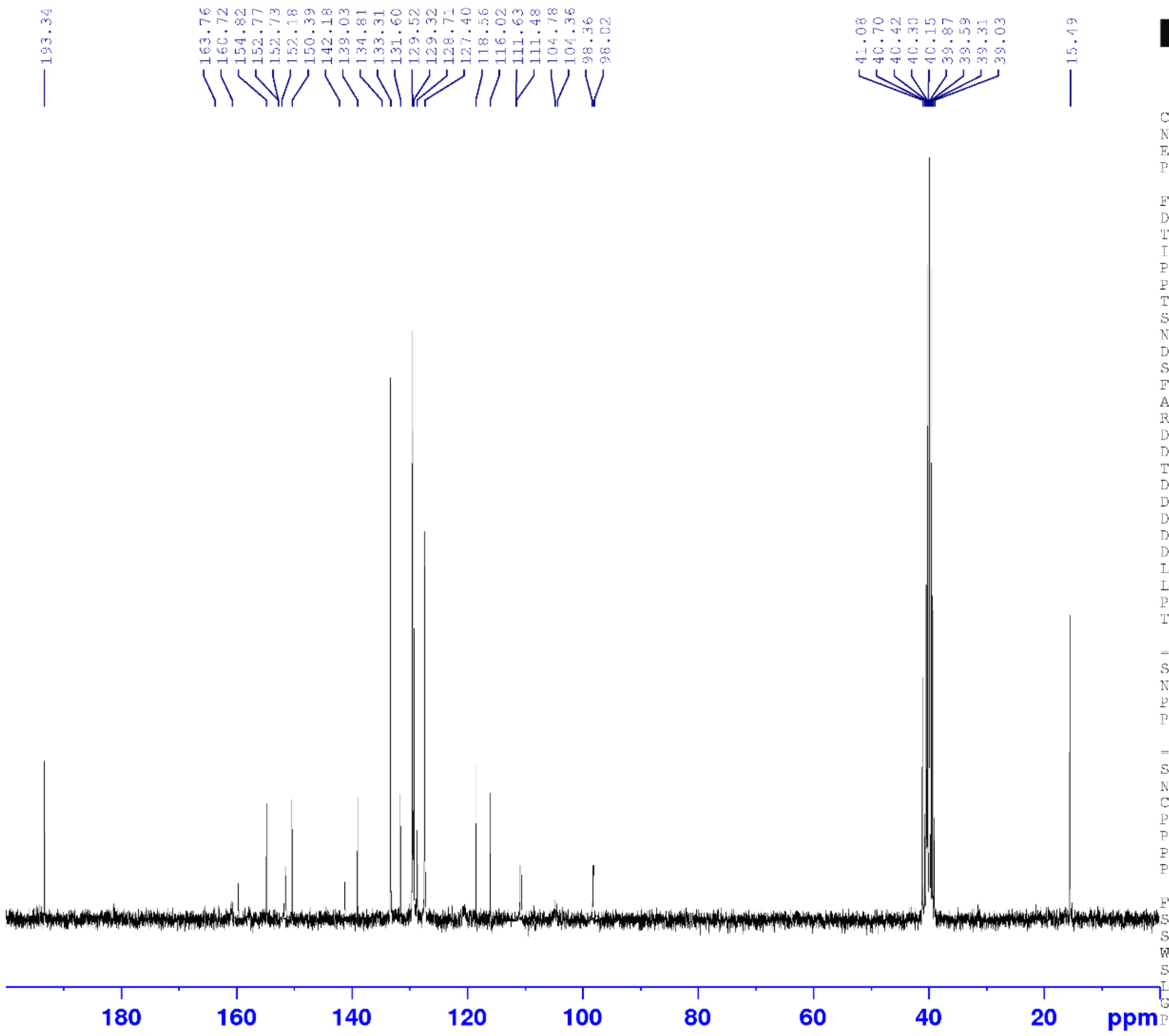

Current Data Parameters
NAME Yusuf Ozkay
EXPNO 353
PROCNO 1

F2 - Acquisition Parameters
Date_ 20160520
Time 16.06
INSTRUM FOURIER300
PROBHD 5 mm DUL 13C-1
PULPROG zgpg
TD 32768
SOLVENT DMSO
NS 2048
DS 4
SWH 24414.063 Hz
FIDRES 0.745058 Hz
AQ 0.6710886 sec
RG 501.187
DW 20.480 usec
DE 6.50 usec
TE 297.5 K
D1 1.00000000 sec
D11 0.03000000 sec
D31 0.00001500 sec
D32 0.89999998 sec
D40 0.00093990 sec
L4 23
L5 26
P32 90.00 usec
TD0 1

======== CHANNEL f1 ========
SFO1 75.4878687 MHz
NUC1 13C
P1 15.00 usec
PLW1 15.00000000 W

======== CHANNEL f2 ========
SFO2 300.1812007 MHz
NUC2 1H
CPDPRG[2 waltz16
PCPD2 90.00 usec
PLW2 10.00000000 W
PLW12 0.20863999 W
PLW13 0.10495000 W

F2 - Processing parameters
SI 32768
SF 75.4803210 MHz
WDW EM
SSB 0
LB 1.00 Hz
GB 0
PC 1.40

Data File: C:\LabSolutions\Data\Analiz\uac\TU-52A_73.lcd

Elmt	Val.	Min	Max	Elmt	Val.	Min	Max	Elmt	Val.	Min	Max	Elmt	Val.	Min	Max	Use Adduct
H	1	9	40	O	2	1	3	S	2	0	2	Ru	2	0	0	H
C	4	11	26	F	1	0	1	Cl	1	0	0	I	3	0	0	
N	3	0	6	P	3	0	0	Br	1	0	1					

Error Margin (ppm): 5
HC Ratio: unlimited
Max Isotopes: 3
MSn Iso RI (%): 10.00

DBE Range: 17.0 - 20.0
Apply N Rule: yes
Isotope RI (%): 1.00
MSn Logic Mode: AND

Electron Ions: both
Use MSn Info: no
Isotope Res: 10000
Max Results: 500

Event#: 1 MS(E+) Ret. Time : 6.653 -> 6.973 - 8.427 -> 9.295 Scan# : 999 -> 1047 - 1265 -> 1395

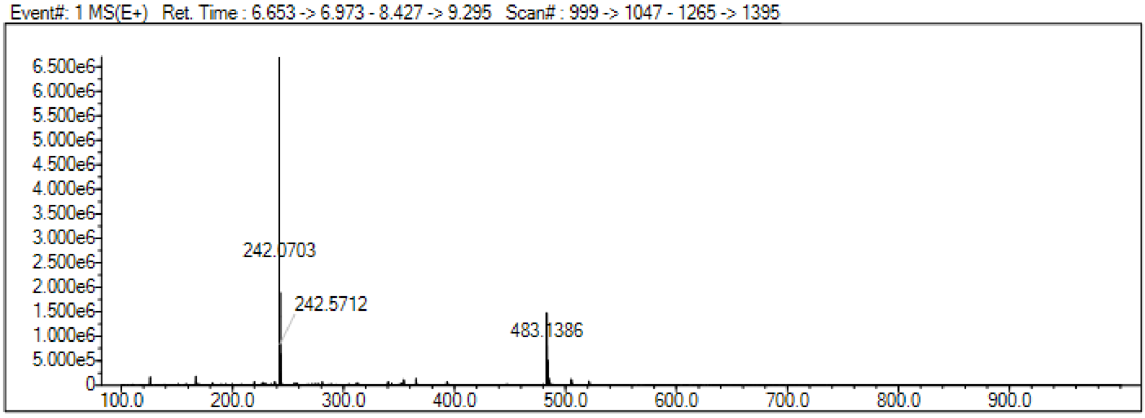

Measured region for 483.1386 m/z

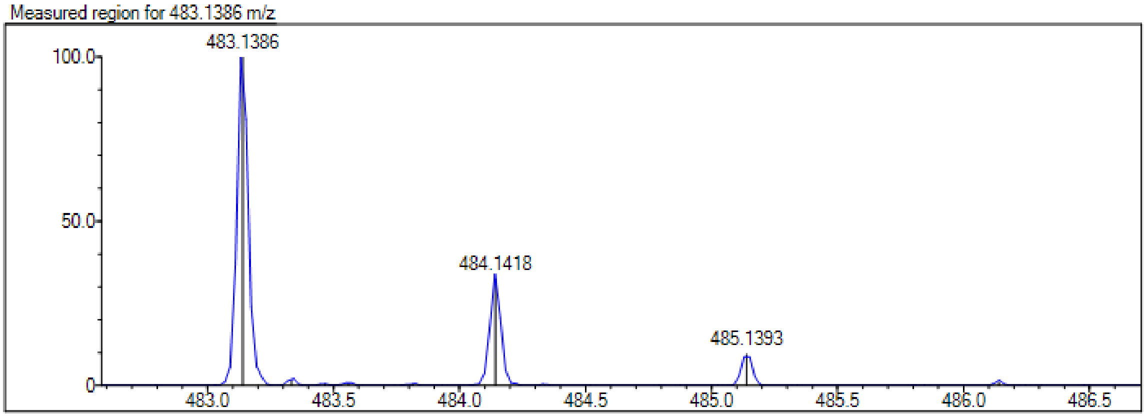

C26 H19 N6 O F S [M+H]+ : Predicted region for 483.1398 m/z

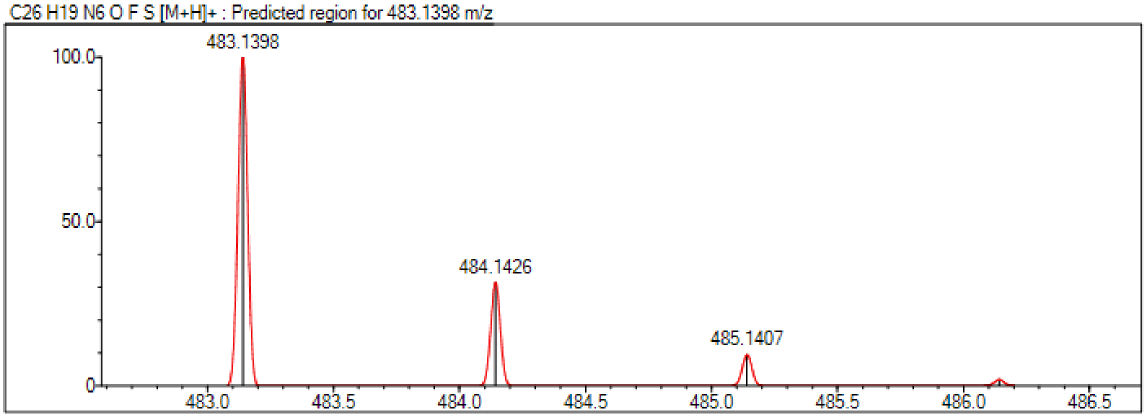

Rank	Score	Formula (M)	Ion	Meas. m/z	Pred. m/z	Df. (mDa)	Df. (ppm)	Iso	DBE
1	83.59	C26 H19 N6 O F S	[M+H]+	483.1386	483.1398	-1.2	-2.48	86.80	20.0

2-(4-(4-Ethyl-5-(2-(4-bromophenyl)-2-oxo-ethylthio)-4H-1,2,4-triazol-3yl)-phenyl)-5(6)-fluoro-1H-benzimidazole (3n)

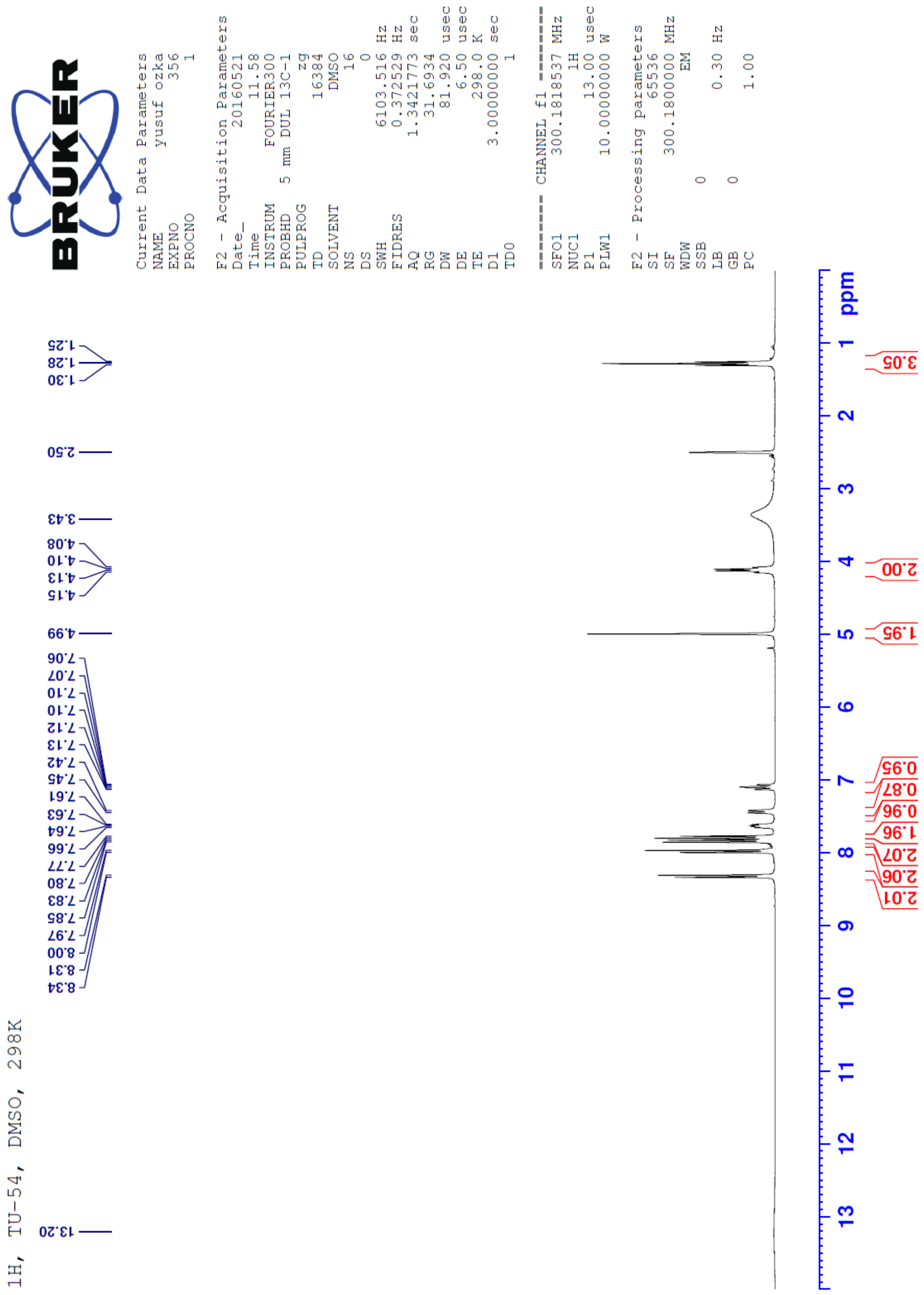

13C, TU-54, DMSO, 298K

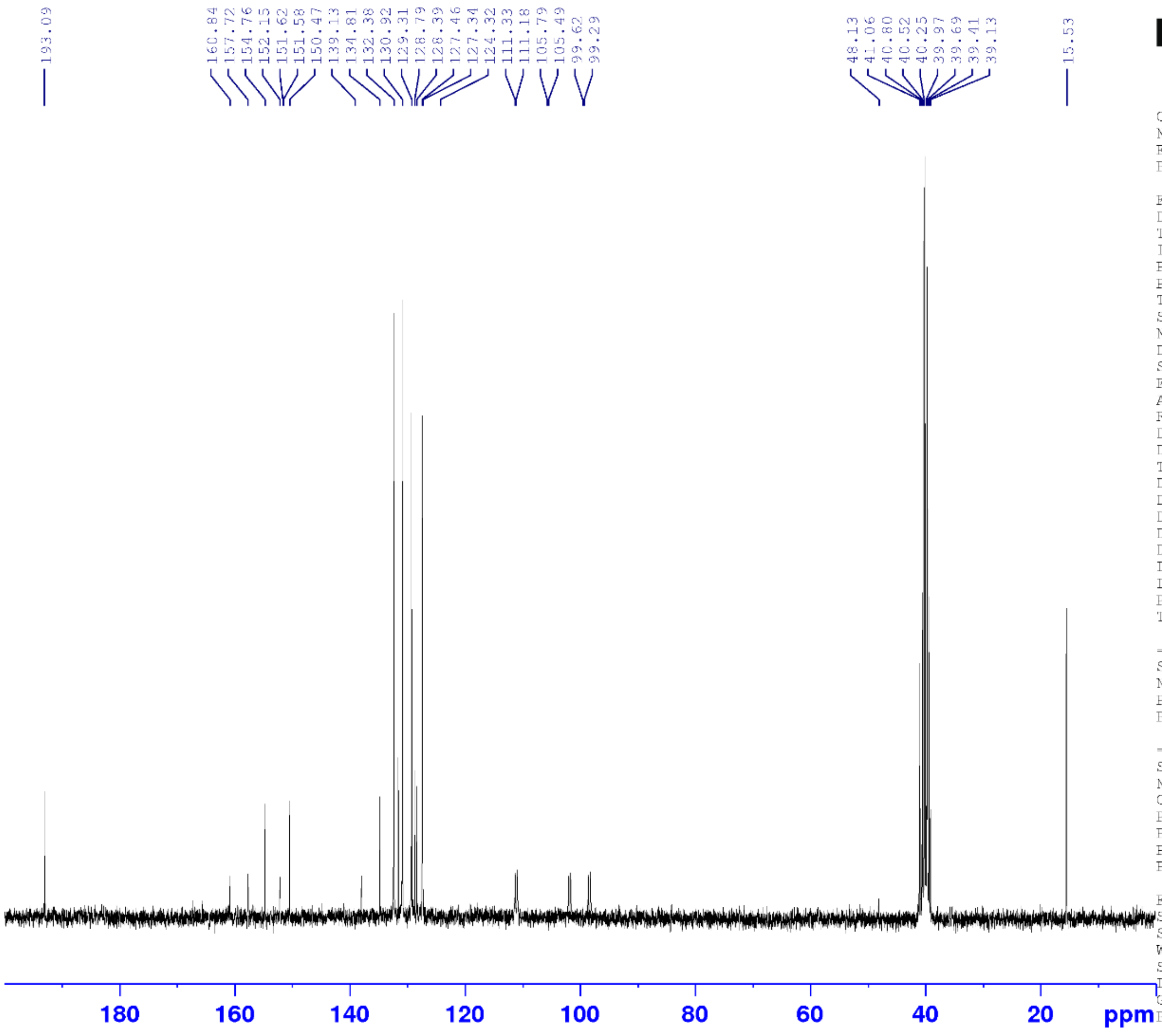

Current Data Parameters
NAME Yusuf Ozkay
EXPNO 357
PROCNO 1

F2 - Acquisition Parameters
Date_ 20160521
Time 12.00
INSTRUM FOURIER300
PROBHD 5 mm DUL 13C-1
PULPROG zgpg
TD 32768
SOLVENT DMSO
NS 2048
DS 4
SWH 24414.063 Hz
FIDRES 0.745058 Hz
AQ 0.6710886 sec
RG 501.187
DW 20.480 usec
DE 6.50 usec
TE 297.3 K
D1 1.00000000 sec
D11 0.03000000 sec
D31 0.00001500 sec
D32 0.89999998 sec
D40 0.00093990 sec
L4 23
L5 26
P32 90.00 usec
TD0 1

======== CHANNEL f1 ========
SFO1 75.4878687 MHz
NUC1 13C
P1 15.00 usec
PLW1 15.00000000 W

======== CHANNEL f2 ========
SFO2 300.1812007 MHz
NUC2 1H
CPDPRG[2 waltz16
PCPD2 90.00 usec
PLW2 10.00000000 W
PLW12 0.20863999 W
PLW13 0.10495000 W

F2 - Processing parameters
SI 32768
SF 75.4803210 MHz
WDW EM
SSB 0
LB 1.00 Hz
GB 0
PC 1.40

Data File: C:\LabSolutions\Data\Analiz\uac\TU-54_76.lcd

Elmt	Val.	Min	Max	Elmt	Val.	Min	Max	Elmt	Val.	Min	Max	Elmt	Val.	Min	Max	Use Adduct
H	1	9	40	O	2	1	3	S	2	0	2	Ru	2	0	0	H
C	4	11	26	F	1	0	1	Cl	1	0	0	I	3	0	0	
N	3	0	6	P	3	0	0	Br	1	0	1					

Error Margin (ppm): 5
HC Ratio: unlimited
Max Isotopes: 3
MSn Iso RI (%): 10.00

DBE Range: 17.0 - 20.0
Apply N Rule: yes
Isotope RI (%): 1.00
MSn Logic Mode: AND

Electron Ions: both
Use MSn Info: no
Isotope Res: 10000
Max Results: 500

Event#: 1 MS(E+) Ret. Time : 7.107 -> 7.253 - 7.907 -> 9.212 Scan# : 1067 -> 1089 - 1187 -> 1383

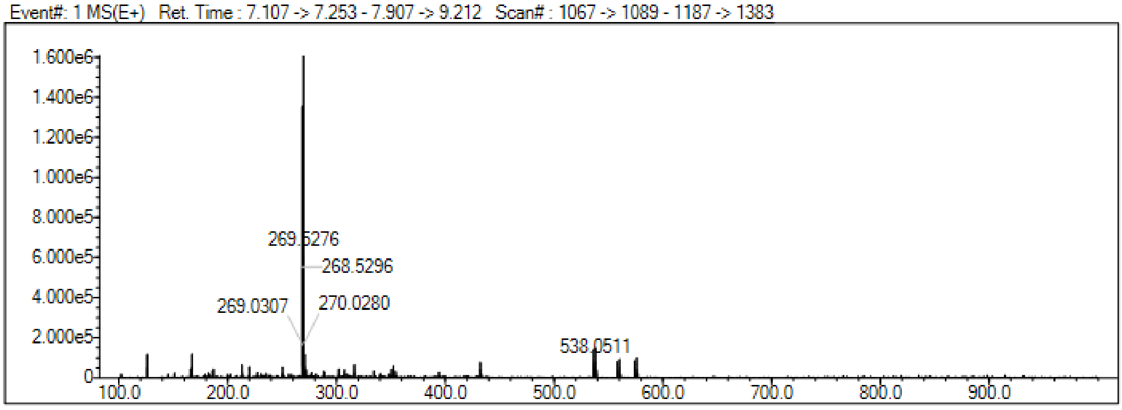

Measured region for 536.0537 m/z

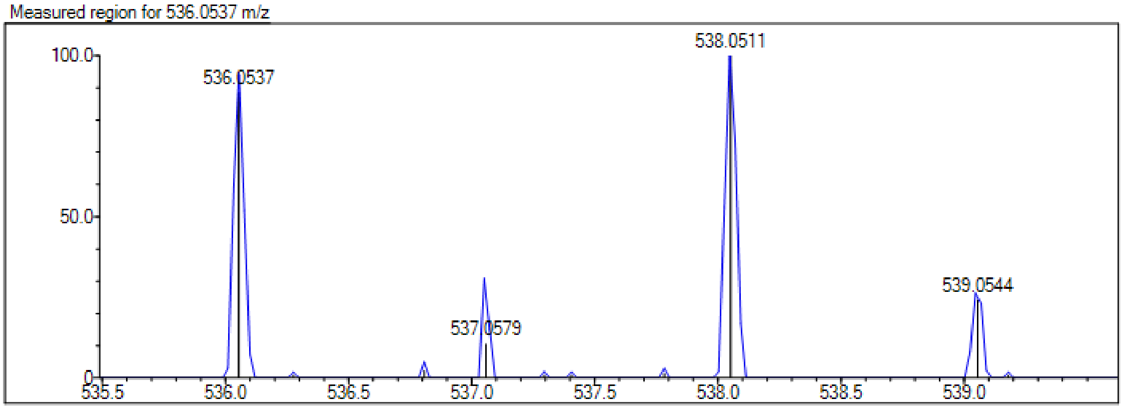

C25 H19 N5 O F S Br [M+H]+ : Predicted region for 536.0550 m/z

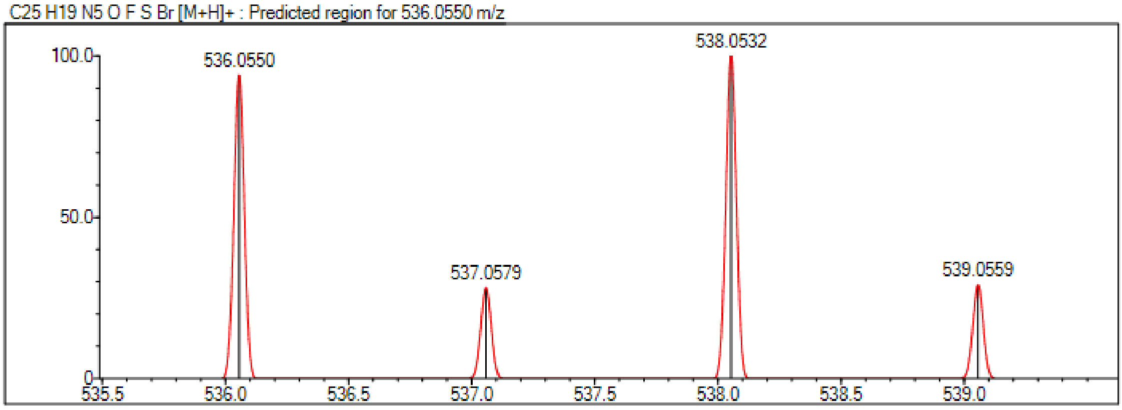

Rank	Score	Formula (M)	Ion	Meas. m/z	Pred. m/z	Df. (mDa)	Df. (ppm)	Iso	DBE
1	71.14	C25 H19 N5 O F S Br	[M+H]+	536.0537	536.0550	-1.3	-2.43	73.78	18.0

2-(4-(4-Ethyl-5-(2-(4-methylphenyl)-2-oxo-ethylthio)-4H-1,2,4-triazol-3yl)-phenyl)-5(6)-fluoro-1H-benzimidazole (3o)

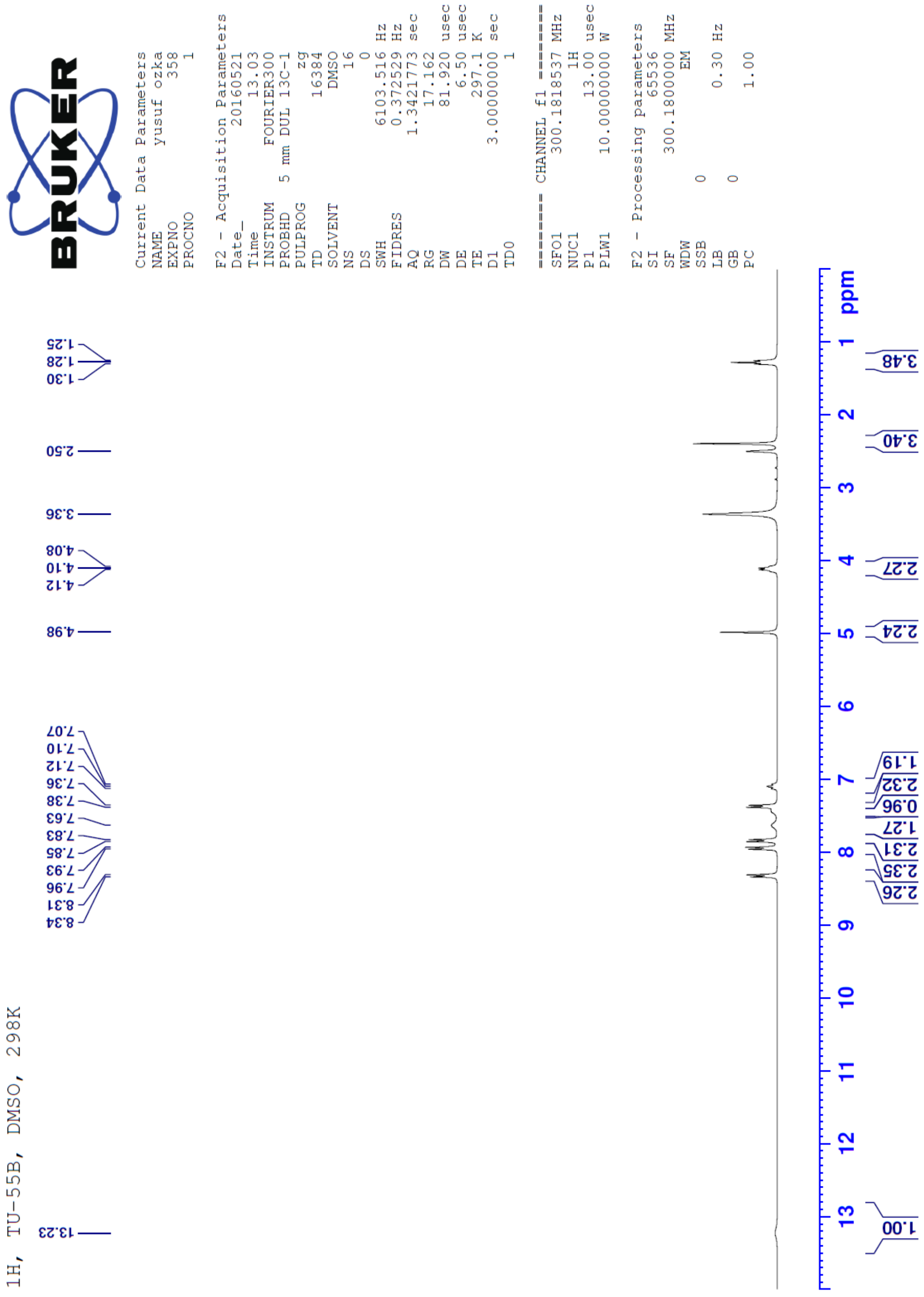

13C, TU-55B, DMSO, 298K

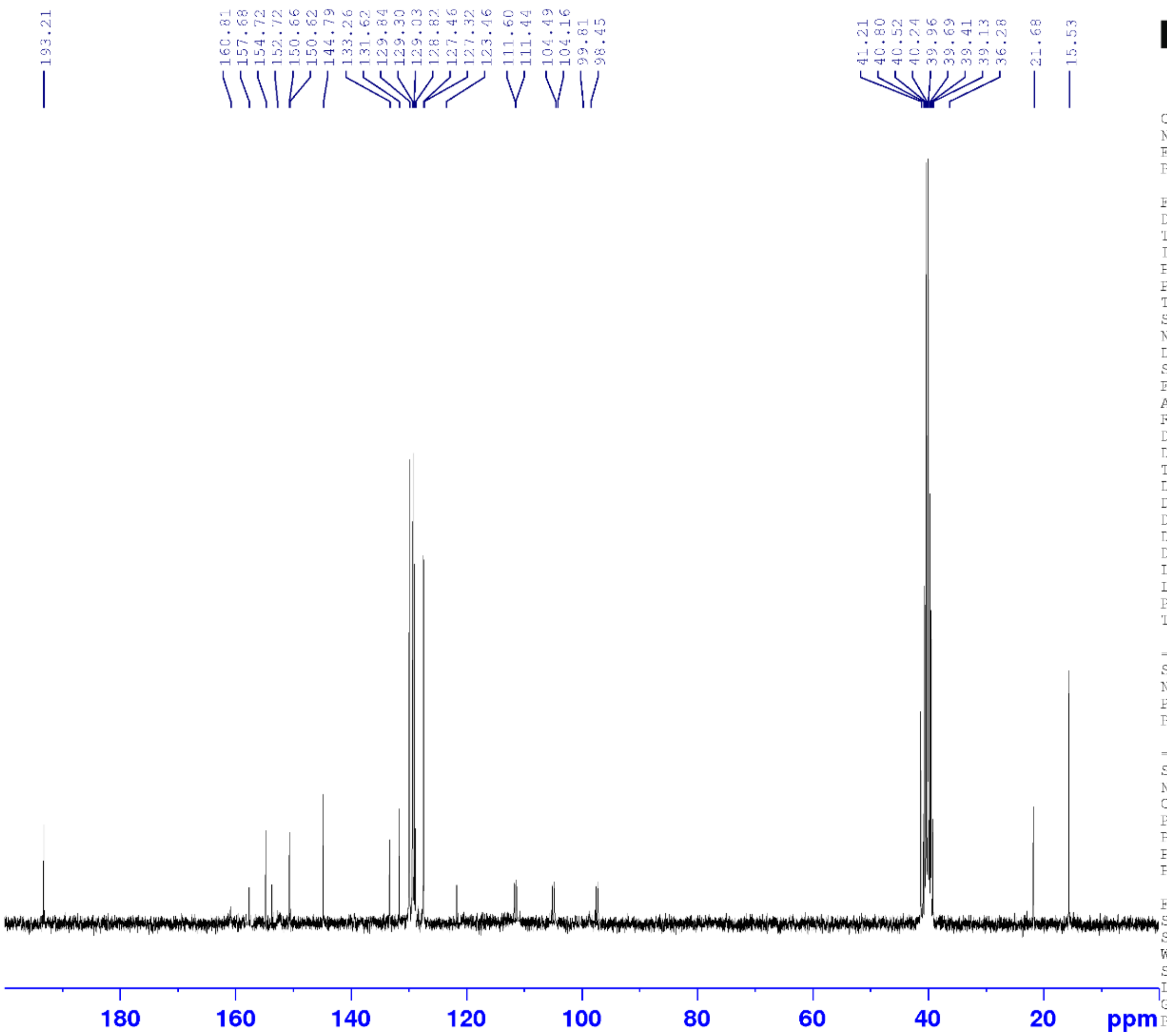

Current Data Parameters

NAME	Yusuf Ozkay
EXPNO	359
PROCNO	1

F2 - Acquisition Parameters

Date_	20160521
Time	13.05
INSTRUM	FOURIER300
PROBHD	5 mm DUL 13C-1
PULPROG	zgpg
TD	32768
SOLVENT	DMSO
NS	2048
DS	4
SWH	24414.063 Hz
FIDRES	0.745058 Hz
AQ	0.6710886 sec
RG	501.187
DW	20.480 usec
DE	6.50 usec
TE	297.6 K
D1	1.00000000 sec
D11	0.03000000 sec
D31	0.00001500 sec
D32	0.89999998 sec
D40	0.00093990 sec
L4	23
L5	26
P32	90.00 usec
TD0	1

======== CHANNEL f1 ========

SFO1	75.4878687 MHz
NUC1	13C
P1	15.00 usec
PLW1	15.00000000 W

======== CHANNEL f2 ========

SFO2	300.1812007 MHz
NUC2	1H
CPDPRG[2	waltz16
PCPD2	90.00 usec
PLW2	10.00000000 W
PLW12	0.20863999 W
PLW13	0.10495000 W

F2 - Processing parameters

SI	32768
SF	75.4803210 MHz
WDW	EM
SSB	0
LB	1.00 Hz
GB	0
PC	1.40

Data File: C:\LabSolutions\Data\Analiz\uac\TU-73_11.lcd

Elmt	Val.	Min	Max	Elmt	Val.	Min	Max	Elmt	Val.	Min	Max	Elmt	Val.	Min	Max	Use Adduct
H	1	15	40	O	2	1	2	S	2	1	1	Ru	2	0	0	H
C	4	12	40	F	1	0	1	Cl	1	0	0	I	3	0	0	
N	3	1	6	P	3	0	0	Br	1	0	0					

Error Margin (ppm):	15	DBE Range:	5.0 - 25.0	Electron Ions:	both
HC Ratio:	unlimited	Apply N Rule:	yes	Use MSn Info:	no
Max Isotopes:	3	Isotope RI (%):	1.00	Isotope Res:	10000
MSn Iso RI (%):	10.00	MSn Logic Mode:	AND	Max Results:	500

Event#: 1 MS(E+) Ret. Time : 6.067 -> 6.947 Scan# : 911 -> 1043

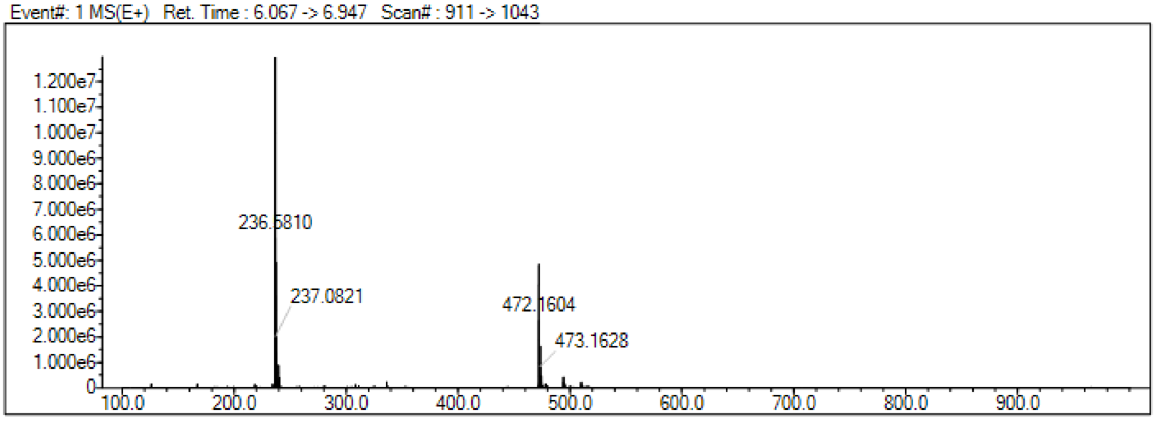

Measured region for 472.1604 m/z

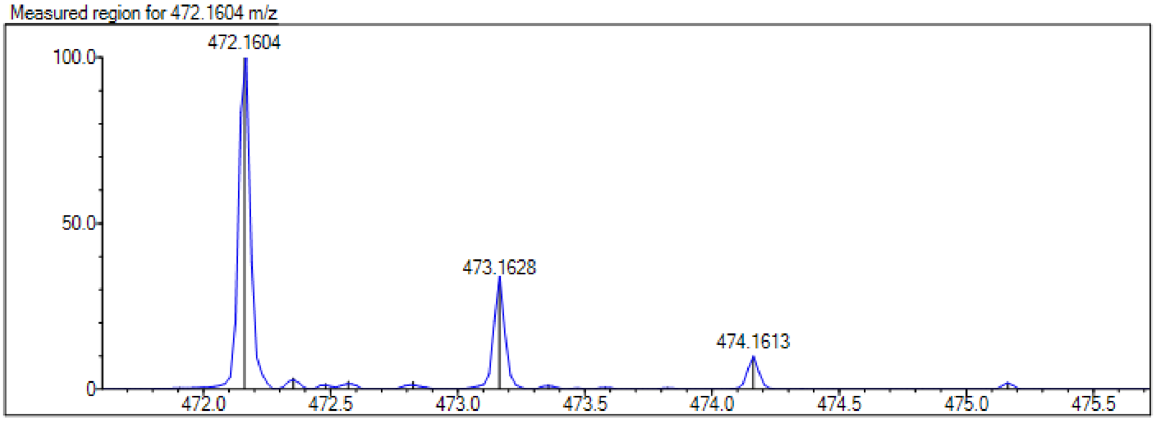

C26 H22 N5 O F S [M+H]+ : Predicted region for 472.1602 m/z

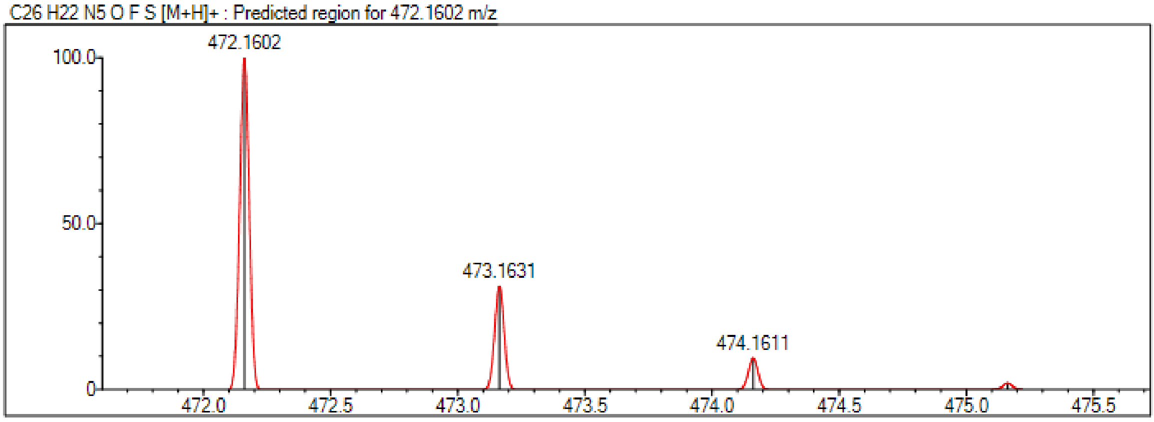

Rank	Score	Formula (M)	Ion	Meas. m/z	Pred. m/z	Df. (mDa)	Df. (ppm)	Iso	DBE
1	92.81	C26 H22 N5 O F S	[M+H]+	472.1604	472.1602	0.2	0.42	92.81	18.0

2-(4-(4-Ethyl-5-(2-(3,4-dihydroxyphenyl)-2-oxo-ethylthio)-4H-1,2,4-triazol-3yl)-phenyl)-5(6)-fluoro-1H- benzimidazole (3p)

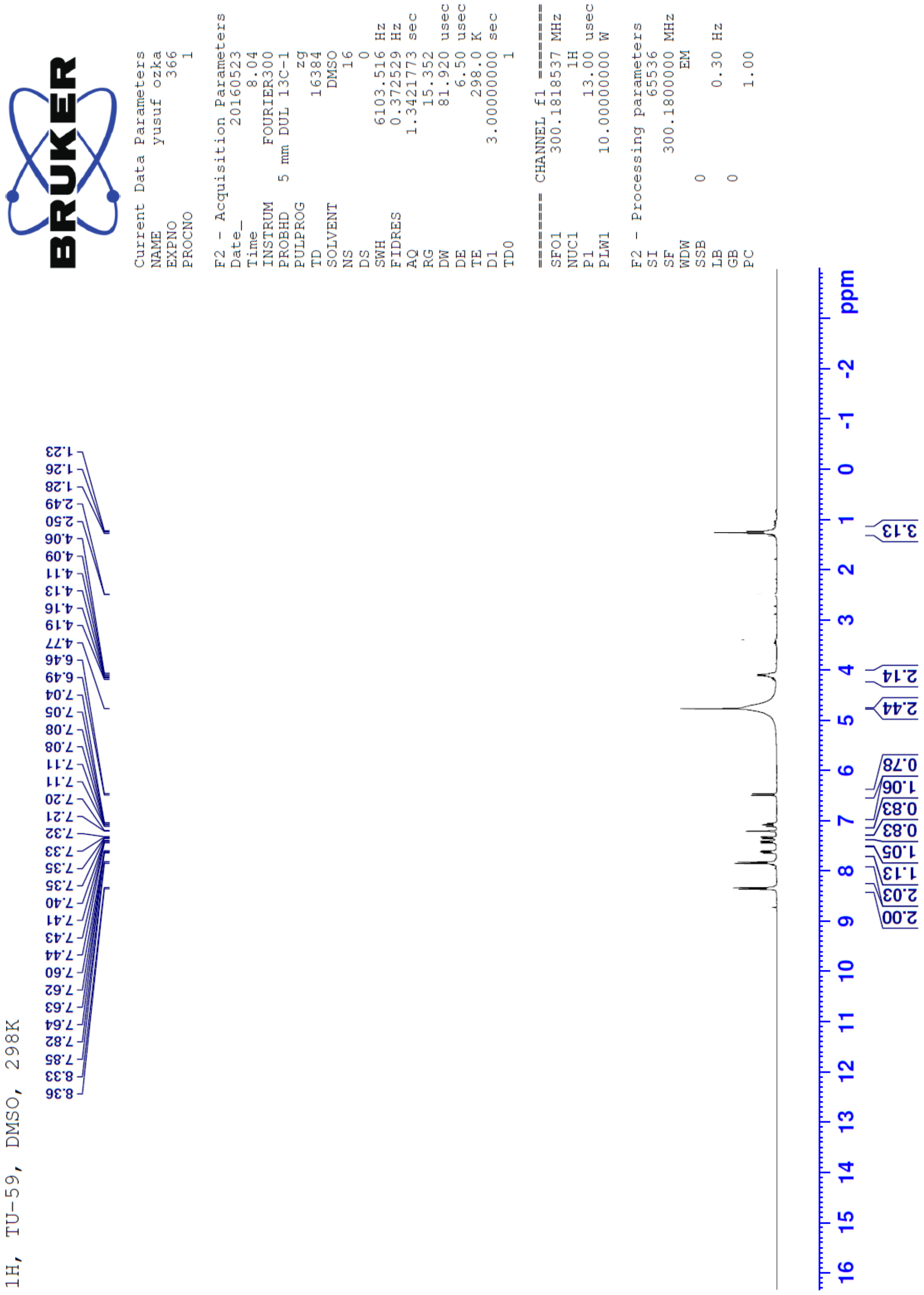

13C, TU-59, DMSO, 298K

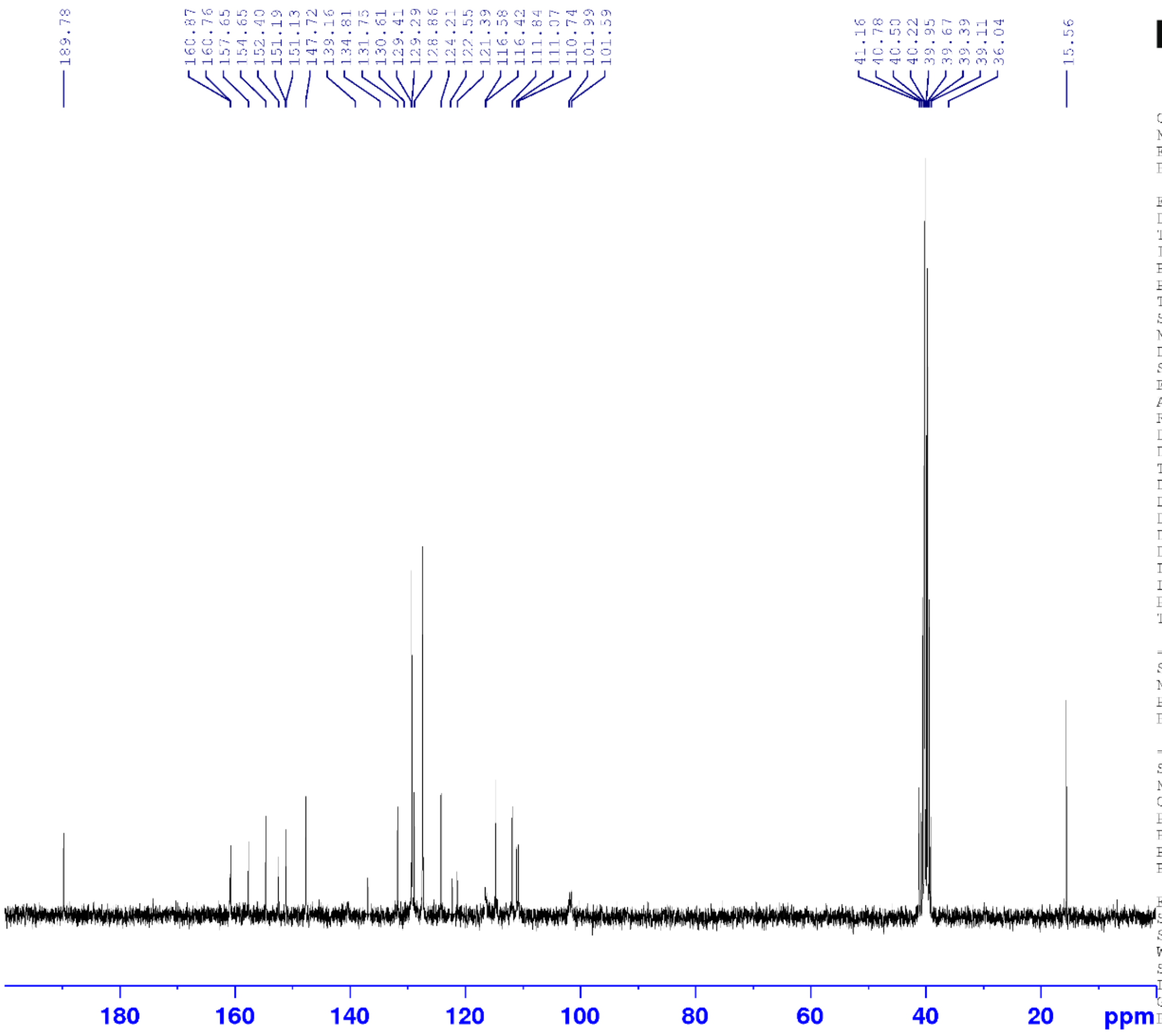

Current Data Parameters
NAME Yusuf Ozkay
EXPNO 367
PROCNO 1

F2 - Acquisition Parameters
Date_ 20160523
Time 8.06
INSTRUM FOURIER300
PROBHD 5 mm DUL 13C-1
PULPROG zgpg
TD 32768
SOLVENT DMSO
NS 2048
DS 4
SWH 24414.063 Hz
FIDRES 0.745058 Hz
AQ 0.6710886 sec
RG 501.187
DW 20.480 usec
DE 6.50 usec
TE 298.0 K
D1 1.00000000 sec
D11 0.03000000 sec
D31 0.00001500 sec
D32 0.89999998 sec
D40 0.00093990 sec
L4 23
L5 26
P32 90.00 usec
TD0 1

======== CHANNEL f1 ========
SFO1 75.4878687 MHz
NUC1 13C
P1 15.00 usec
PLW1 15.00000000 W

======== CHANNEL f2 ========
SFO2 300.1812007 MHz
NUC2 1H
CPDPRG[2 waltz16
PCPD2 90.00 usec
PLW2 10.00000000 W
PLW12 0.20863999 W
PLW13 0.10495000 W

F2 - Processing parameters
SI 32768
SF 75.4803210 MHz
WDW EM
SSB 0
LB 1.00 Hz
GB 0
PC 1.40

Data File: C:\LabSolutions\Data\Analiz\uac\TU-59_79.lcd

Elmt	Val.	Min	Max	Elmt	Val.	Min	Max	Elmt	Val.	Min	Max	Elmt	Val.	Min	Max	Use Adduct
H	1	9	40	O	2	1	3	S	2	0	2	Ru	2	0	0	H
C	4	11	26	F	1	0	1	Cl	1	0	0	I	3	0	0	
N	3	0	6	P	3	0	0	Br	1	0	1					

Error Margin (ppm): 5
HC Ratio: unlimited
Max Isotopes: 3
MSn Iso RI (%): 10.00

DBE Range: 17.0 - 20.0
Apply N Rule: yes
Isotope RI (%): 1.00
MSn Logic Mode: AND

Electron Ions: both
Use MSn Info: no
Isotope Res: 10000
Max Results: 500

Event#: 1 MS(E+) Ret. Time : 6.040 -> 6.307 - 7.453 -> 8.733 Scan# : 907 -> 947 - 1119 -> 1311

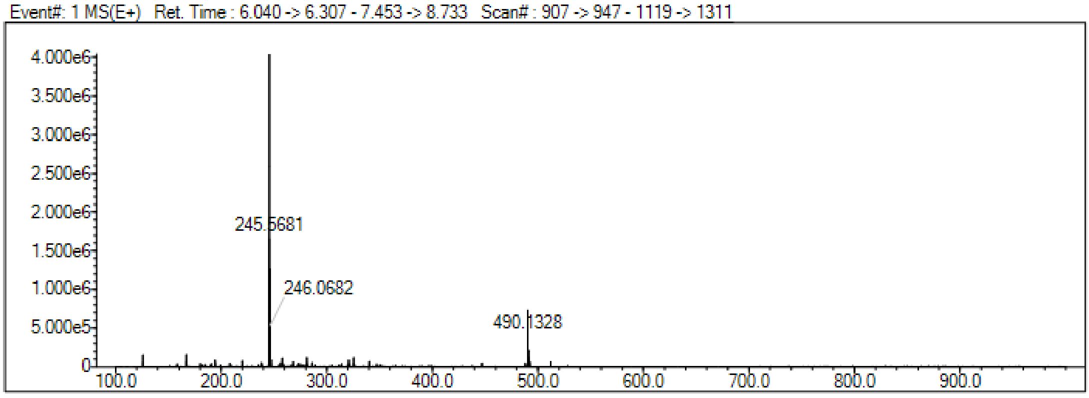

Measured region for 490.1328 m/z

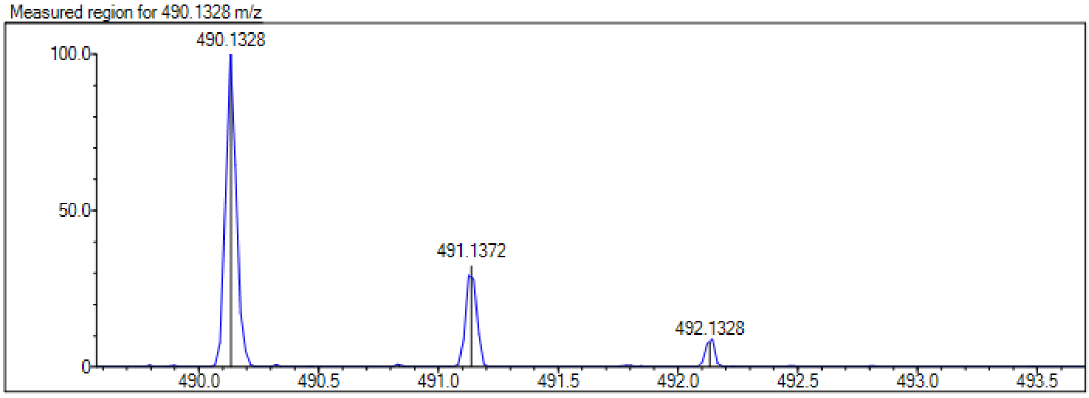

C25 H20 N5 O3 F S [M+H]+ : Predicted region for 490.1344 m/z

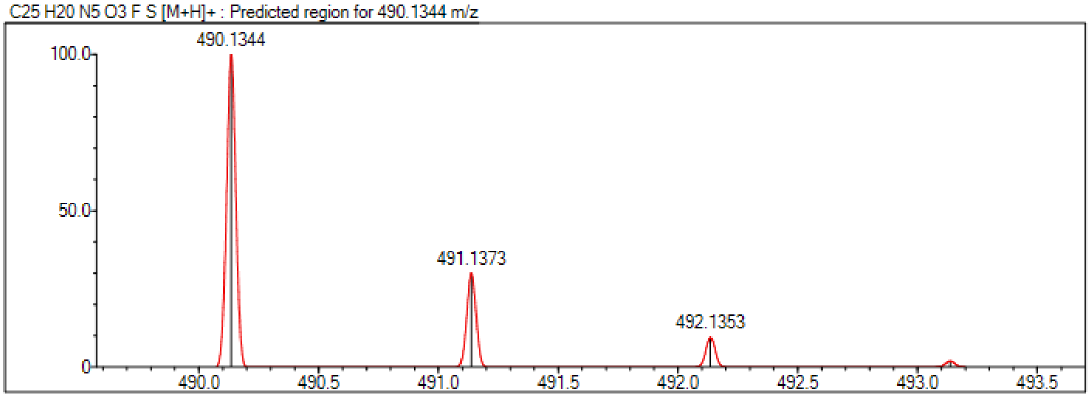

Rank	Score	Formula (M)	Ion	Meas. m/z	Pred. m/z	Df. (mDa)	Df. (ppm)	Iso	DBE
1	76.02	C25 H20 N5 O3 F S	[M+H]+	490.1328	490.1344	-1.6	-3.26	80.57	18.0

Structure of the synthesized compounds were elucidated by IR, ^{1}H NMR, ^{13}C NMR and HRMS analysis. In ^{1}H-NMR spectra, protons belonging to methylene bridge were recorded between 4.80 ppm and 2.64 ppm. Methyl protons in the methyl substituted derivatives (**5a-5d** and **5i-5l**) had singlet peak at 3.71 ppm or 3.72 ppm. Ethyl protons in the ethyl substituted derivatives (**5e-5h** and **5m-5p**) were observed as triplet and quartet peaks at 1.26 ppm- 1.28 ppm and 4.10 ppm- 4.12 ppm, respectively. Protons of aromatic rings were recorded between 6.81 ppm and 8.34 ppm and benzimidazole NH had singlet peak over 13.20 ppm. In ^{13}C-NMR, aliphatic carbons recorded between 15 ppm and 49 ppm and aromatic carbons gave peak from 97 ppm to 163 ppm. Carbonyl carbon were recorded around 190 ppm. Carbon-fluorine splitting in the fluorinated compounds were observed as expected. In high resolution mass spectrum, all masses were found as expected with maximum 5 ppm difference. In the mass spectrum, all masses were matched with the expected M+H values

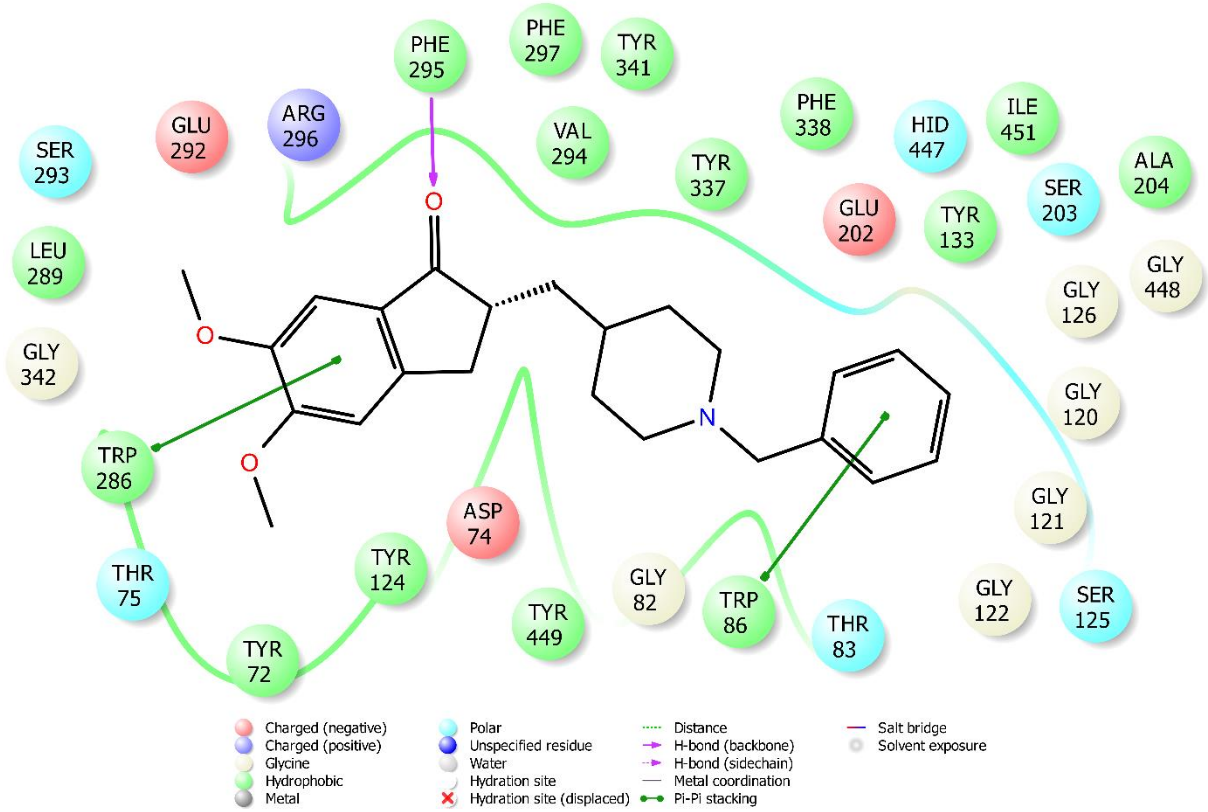

Figure S1. Two-dimensional interaction mode of donepezil in the enzyme active site of AChE.

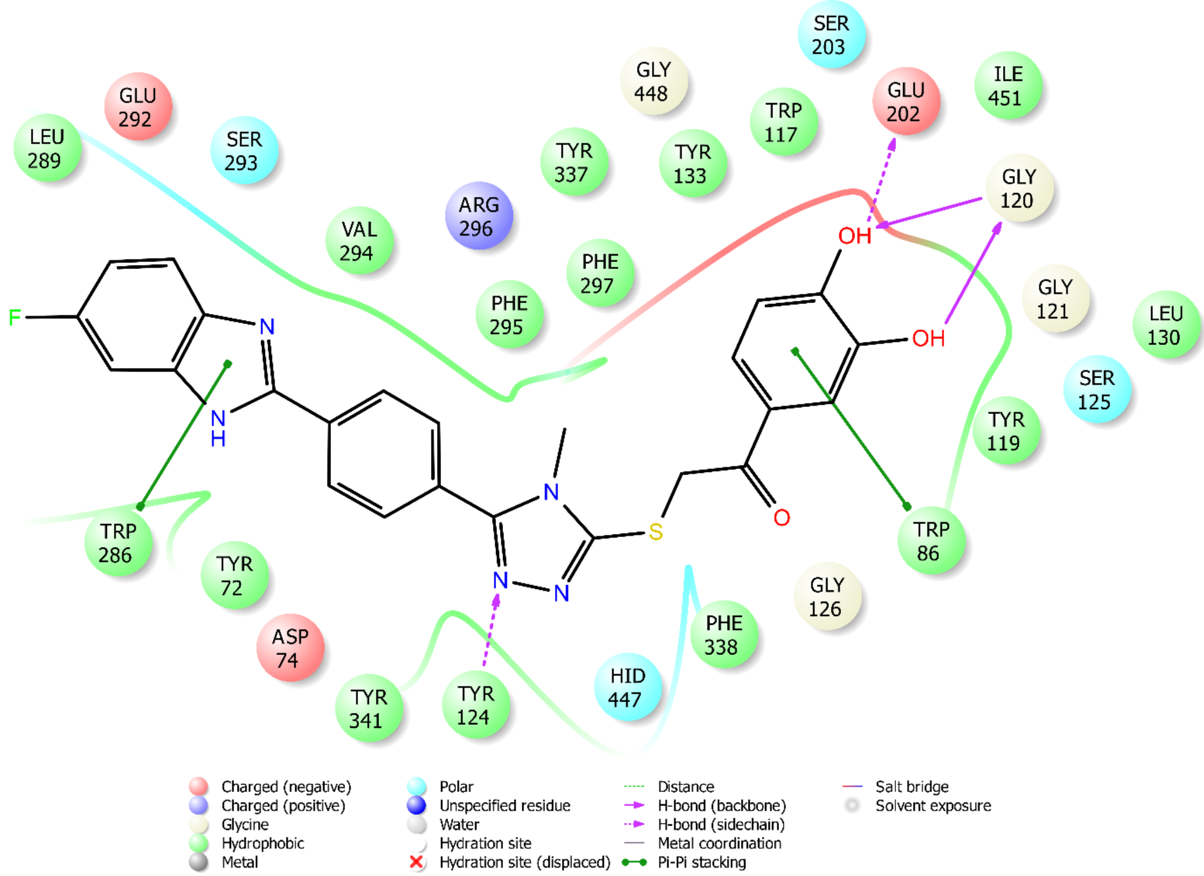

Figure S2. Two-dimensional interaction mode of compound **5l** in the enzyme active site of AChE.

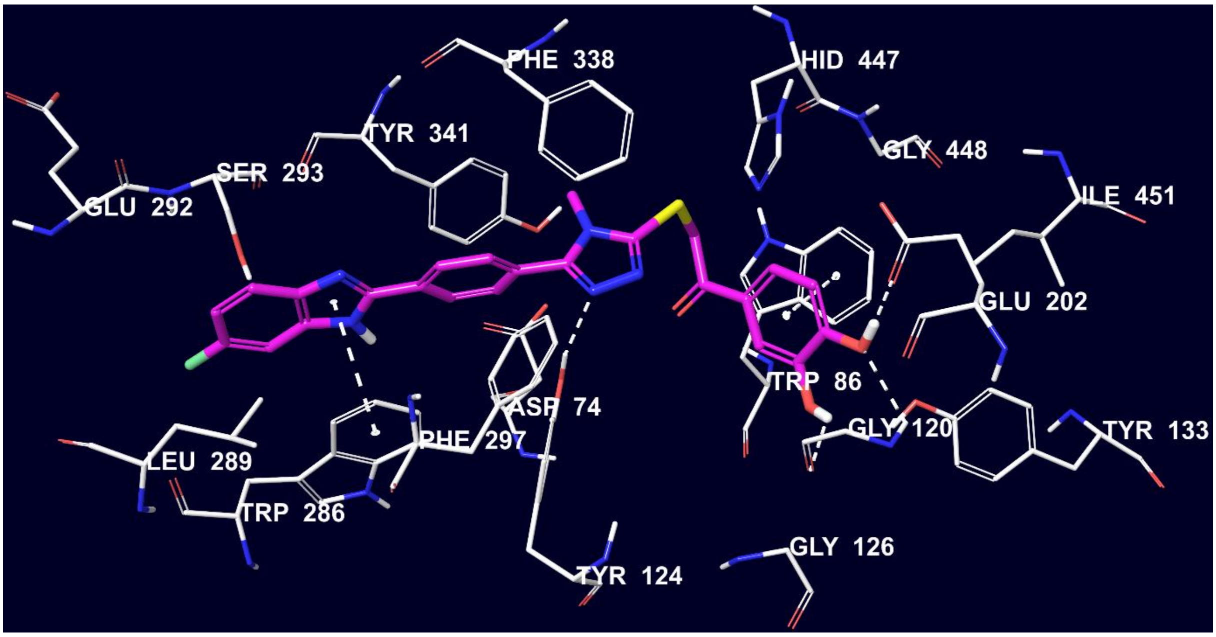

Figure S3. The interacting mode of compound **5l** in the active region of AChE. The inhibitor, colored with pink, and the important residues, colored with white, in the active site of the enzyme are presented by tube model.

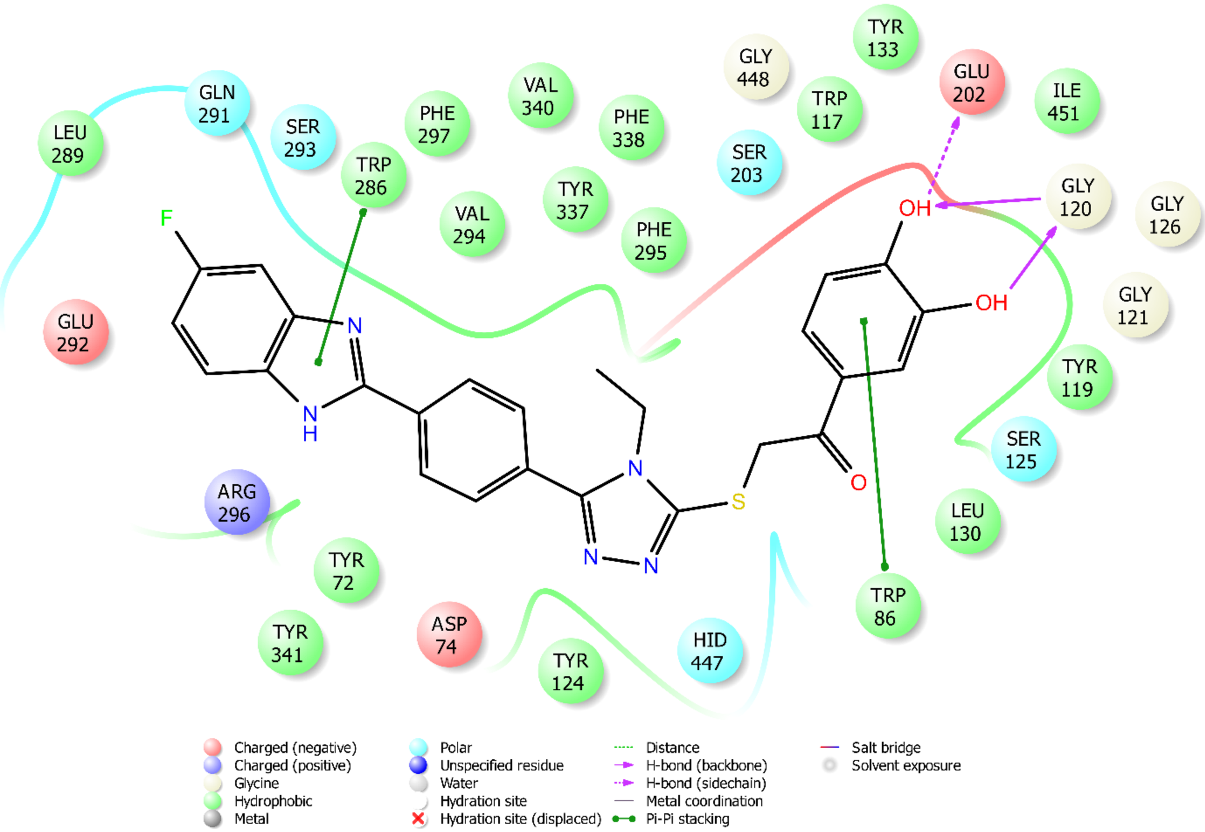

Figure S4. Two-dimensional interaction mode of compound **5p** in the enzyme active site of AChE.

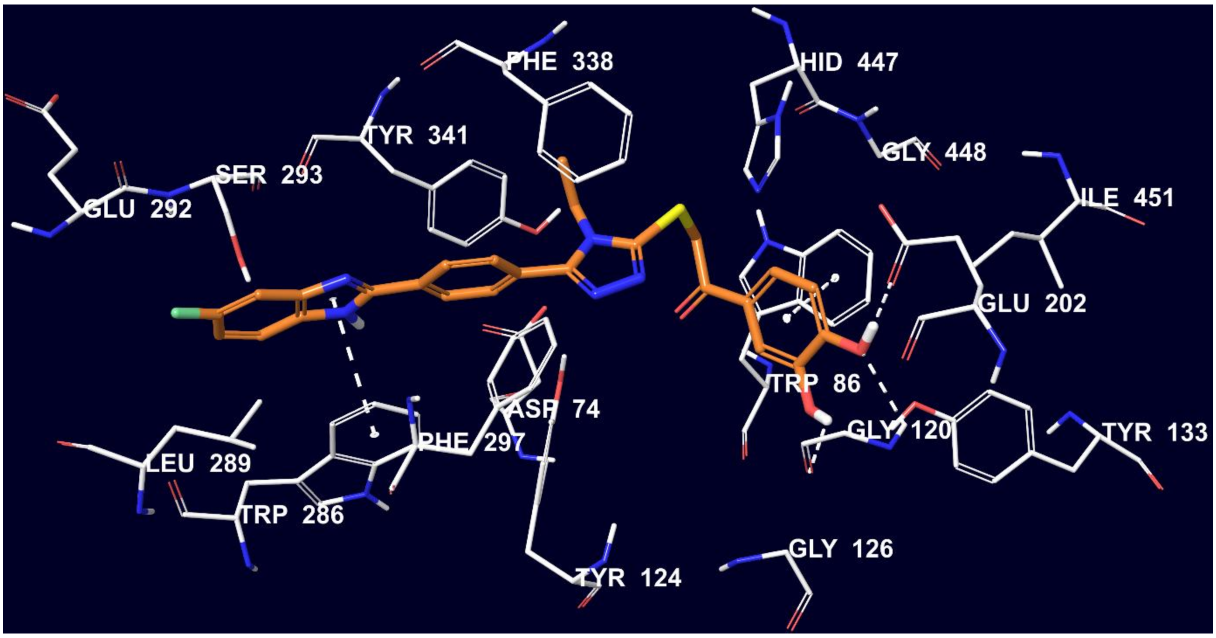

Figure S5. The interacting mode of compound **5p** in the active region of AChE. The inhibitor, colored with orange, and the important residues, colored with white, in the active site of the enzyme are presented by tube model.

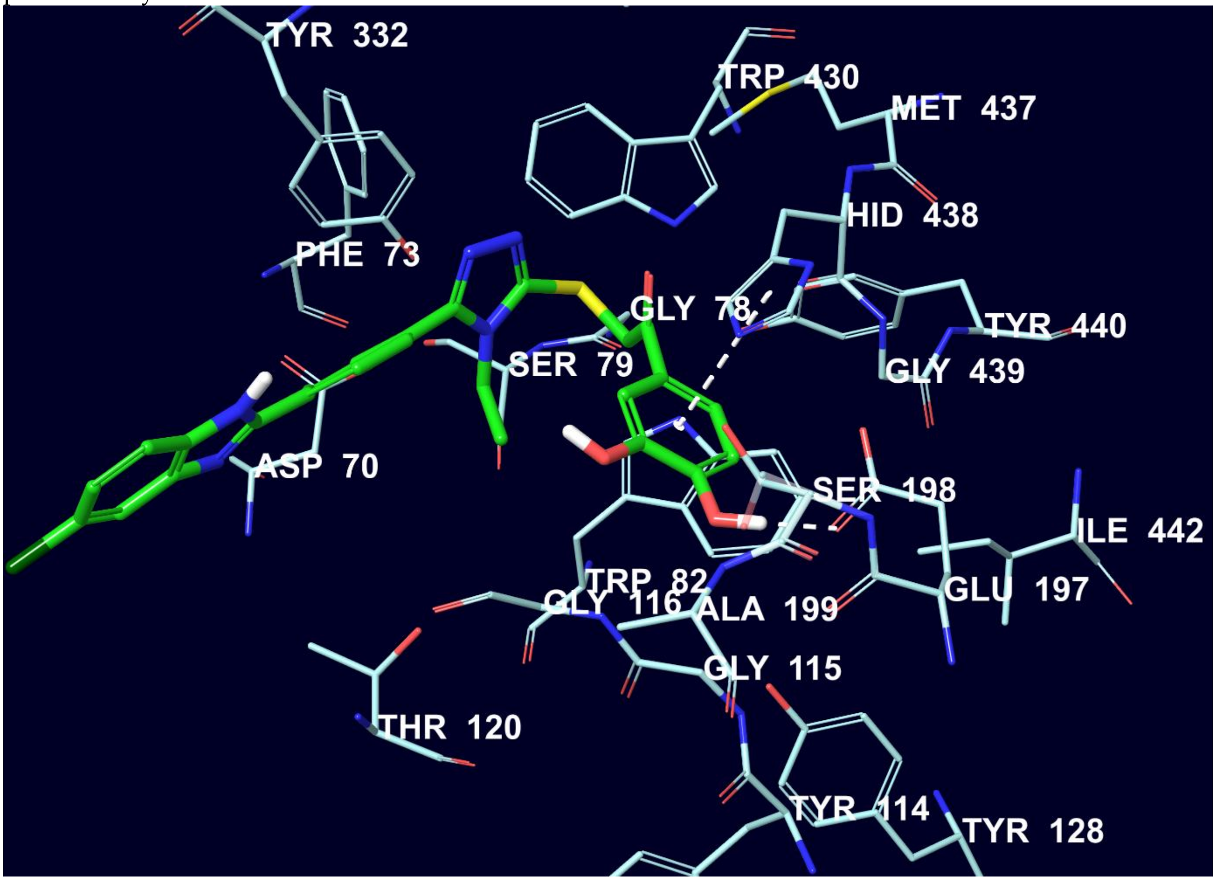

Figure S6. The interacting mode of compound **5h** in the active region of BChE. The inhibitor, colored with green, and the important residues, colored with turquoise, in the active site of the enzyme are presented by tube model.
